# Dimensions of perceived algorithmic control and endorsement of minimum-only execution in platform delivery work

**DOI:** 10.3389/fpsyg.2026.1937810

**Published:** 2026-10-07

**Authors:** Haixin Zhang, Yingji Li, Qingxia Li

**Affiliations:** 1 School of Accounting, Tongling University, Tongling, Anhui, China; 2 School of Humanities and Management, Yunnan University of Chinese Medicine, Kunming, China; 3 School of Government Management, Henan University of Economics and Law, Zhengzhou, China

**Keywords:** algorithmic management, emotional exhaustion, emotional feigning, human-AI symbiosis, minimum-only execution, perceived algorithmic control, platform work

## Abstract

An overall perception of algorithmic control may obscure how specific work experiences relate to emotional reports and minimum-only execution. Drawing on affective events theory, conservation of resources theory, and emotional labor theory, this study examines how control-related work experiences and emotions relate to endorsement of minimum-only execution. Single-wave survey data from 650 platform delivery workers in China were analyzed using ordinary partial least squares structural equation modeling. The primary model jointly includes temporal demands and pace adjustment, response demands and reported responses, and trajectory-recording awareness as separate reflective constructs. Conditional on the other dimensions and both emotional variables, temporal and response dimensions were positively associated with minimum-only execution (*β* = 0.339 and 0.242), whereas the trajectory dimension was negatively associated (*β* = −0.183). Trajectory-recording awareness was positively associated with exhaustion and feigning; its positive total indirect association accompanied a total association with minimum-only execution that was not statistically distinguishable from zero. Exhaustion and feigning were each positively associated with minimum-only execution, but their conditional association and the indirect associations involving both were nonsignificant. The common-PAC diagnostic further indicated that a single control representation obscured differences in the three dimensions’ external associations. These findings highlight the value of assessing deadlines and pacing, response expectations, and recording practices separately, while considering both workers’ resource depletion and emotional display regulation in platform work design.

## 1 Introduction

Digital platforms increasingly govern work through algorithmic management systems that allocate orders, set delivery deadlines, issue prompts, track routes, evaluate performance, and apply rewards or penalties. This creates an asymmetrical managerial relationship: workers coordinate continuously through a platform system while that system structures the pace, visibility, and evaluation of their labor. Recent studies show that algorithmic management can create both beneficial and harmful conditions for gig-worker wellbeing depending on autonomy and security preferences (Felix et al., 2023), and that its associations with delivery-worker engagement depend on challenge and threat appraisals (Li et al., 2025). A related question is whether an overall perception of control adequately summarizes how specific work experiences relate to emotional reports and endorsement of minimum-only execution.

Delivery workers encounter algorithmic management through concrete demands concerning time, response, and movement: deadlines, countdowns, order notifications, customer messages, location records, and route tracking. These experiences need not carry the same meaning for workers or share the same associations with their behavior. Existing research describes algorithmic management through task allocation, monitoring, evaluation, and discipline, while recent work links perceived control to voice through fairness judgments (Liang et al., 2026). By examining temporal, response, and trajectory-related experiences together, this study asks whether an overall perception of control conceals differences in their relationships with emotional experiences and minimum-only execution.

Research on platform labor has examined visible resistance, coping strategies, noncompliance, turnover intentions, voice, and changes in service behavior (Wu et al., 2023; Yu et al., 2022; Liang et al., 2026). A related question concerns workers’ endorsement of statements about doing only what platform requirements prescribe. This focus connects platform work to organizational research separating required role performance from discretionary contribution (Williams and Anderson, 1991; Organ, 1997; Macey and Schneider, 2008), and to evidence that outward rule following varies in depth and meaning (Hu et al., 2020). We use minimum-only execution to describe self-reported endorsement of statements about performing only prescribed or basic actions within platform requirements.

The emotional experiences accompanying control-related demands also warrant examination. Studies connect algorithmic management to technostress, work-intensity appraisals, autonomy, and gig-worker wellbeing (Cram et al., 2022; Parent-Rocheleau and Parker, 2022; Felix et al., 2023; Li et al., 2025). Resource depletion and outward emotion regulation offer two relevant perspectives: workers may feel drained while also needing to suppress dissatisfaction during service encounters (Hobfoll, 1989; Grandey, 2000; Grandey and Sayre, 2019). Emotional exhaustion describes a depleted state, whereas emotional feigning concerns masking feelings while maintaining an acceptable display. We examine how these two experiences relate to each control-related dimension and to minimum-only execution when included in the same model.

Using survey responses from 650 platform delivery workers, this study examines temporal, response, and trajectory-related experiences jointly in relation to emotional exhaustion, emotional feigning, and minimum-only execution. A complementary diagnostic assesses whether a common perceived algorithmic control (PAC) factor adequately summarizes these dimensions’ external relations. Together, these analyses connect research on platform control and worker responses with the scope of rule following (Rosenblat and Stark, 2016; Wood et al., 2019; Kellogg et al., 2020; Cram et al., 2022; Duggan et al., 2023). The distinction matters because a common representation of control may conceal how time-related demands and responses, prompt responsiveness, and awareness of tracking relate to workers’ emotional experiences and their endorsement of doing only what platform requirements prescribe.

## 2 Theoretical background and research questions

### 2.1 Algorithmic control in platform delivery work

Algorithmic control has become a central mechanism through which digital platforms organize and govern labor. It relies on data-driven systems to allocate tasks, monitor work processes, evaluate performance, and align workers’ behavior with organizational objectives. Kellogg et al. (2020) describe algorithms as reshaping the terrain of organizational control, while Wood et al. (2019) show that platform-based algorithmic control can coexist with apparent flexibility while producing intensive work demands. In app-based gig work, platform systems coordinate task allocation, performance evaluation, rewards, and behavioral alignment (Duggan et al., 2020, 2023). Accordingly, algorithmic control denotes the platform systems and managerial rules through which delivery workers encounter task allocation, monitoring, evaluation, and discipline.

Platform delivery work is particularly suitable for examining this relationship because the labor process is mobile, time-sensitive, customer-facing, and continuously mediated by platform applications. Food delivery platforms use algorithmic systems to assign and evaluate work while constraining schedules and activities (Griesbach et al., 2019). In China, delivery workers encounter algorithms through temporality, affect, and gamification rather than as distant technical infrastructures (Sun, 2019). Related studies show that algorithmic management shapes service behavior and coping responses (Wu et al., 2023), working-time regimes and spatial coordination (Heiland, 2021, 2022), worker wellbeing under different autonomy and security preferences (Felix et al., 2023), work engagement through stress appraisals (Li et al., 2025), and voice through fairness judgments (Liang et al., 2026). Together, this research shows that human-AI interaction in platform work is experienced through how quickly workers must move and respond, how continuously their routes are made visible, and how much recourse they have when system rules conflict with local conditions.

Studies of scheduling, monitoring, spatial coordination, and worker visibility identify recurring control-related experiences in delivery work (Griesbach et al., 2019; Heiland, 2021, 2022; Duggan et al., 2023). Work-design and scale-development research also distinguishes the functions of algorithmic management (Parent-Rocheleau and Parker, 2022; Parent-Rocheleau et al., 2024). The analysis distinguishes three dimensions of workers’ reported experience. Temporal algorithmic control (TAC) covers time-related requirements and pace adjustment. Response algorithmic control (RAC) covers prompt-related response requirements and reported checking, replying, or immediate action, with conceptual links to workplace telepressure (Barber and Santuzzi, 2015). Trajectory algorithmic control (TRC) primarily captures awareness that location, routes, and delivery movements are recorded. TAC and RAC thus combine demands with reported responses, whereas TRC emphasizes recording awareness.

### 2.2 Minimum-only execution in platform delivery work

Organizational research distinguishes required role performance from contributions beyond formal obligations (Williams and Anderson, 1991; Organ, 1997; Macey and Schneider, 2008). Here, minimum-only execution denotes self-reported endorsement of statements about performing only prescribed or basic actions within platform requirements. Work-to-rule and surface-compliance research informs this focus on the scope and meaning of rule following (Hale and Borys, 2013; Hu et al., 2020).

Minimum-only execution is closest to work-to-rule, which combines adherence to formal requirements with withholding activity beyond them (Hale and Borys, 2013; Alcadipani et al., 2018). It focuses on the scope of reported action: surface compliance distinguishes outward rule following from internalization (Hu et al., 2020), and organizational citizenship behavior covers a wider range of discretionary contributions (Organ, 1997). Withdrawal, deviance, and everyday resistance additionally encompass avoidance, norm violation, harm, or opposition (Hanisch and Hulin, 1990; Robinson and Bennett, 1995; Bennett and Robinson, 2000; Scott, 1985; Alcadipani et al., 2018). Supplementary Appendix S3 compares these constructs with the administered items.

Quiet quitting also includes minimal work contribution, but Patel et al. (2025) additionally assess workers’ emotional evaluations of contributing minimally or contributing more. The present PC measure shares the minimal-contribution content and focuses on endorsement of action-related statements.

Platform delivery provides a useful setting for studying this pattern because formal requirements are visible while aspects of service remain discretionary. Deadlines, ratings, route records, and automated evaluations structure required performance (Griesbach et al., 2019; Sun, 2019; Heiland, 2021, 2022; Duggan et al., 2023), yet workers can vary proactive communication, additional assistance, and attention to customer needs. Platform research documents consent, coping, workarounds, and low-visibility resistance within these conditions (Galière, 2020; Wu et al., 2023; Yu et al., 2022; Huang, 2025). The present study examines how control-related experiences and emotional states covary with endorsement of minimum-only execution statements.

### 2.3 Emotional experiences in platform delivery work

Affective events theory treats work events as relevant to employees’ feelings, attitudes, and behavior (Weiss and Cropanzano, 1996). In platform delivery, time pressure, response demands, route tracking, customer ratings, and evaluation make managerial requirements immediate and personally consequential. Studies connect these conditions to surveillance experiences, technostress, workload, autonomy, and fairness judgments (Cram et al., 2022; Parent-Rocheleau and Parker, 2022; Wiener et al., 2023; Li et al., 2025). This literature motivates examining emotional experiences alongside reported control-related demands and worker behavior.

Emotional exhaustion captures the resource-depletion side of these emotional responses. It refers to a state in which workers feel emotionally drained and lack the energy needed to sustain work involvement. Prior research has shown that emotional exhaustion is associated with poorer work attitudes, job performance, and organizational citizenship behaviors (Cropanzano et al., 2003). This construct is particularly relevant to platform delivery work because algorithmic management turns time limits, surveillance, automated discipline, and customer feedback into recurring job demands. Recent studies of digital labor platforms and gig work show that algorithmic management, especially surveillance, control, evaluation, and discipline, can contribute to burnout or exhaustion among gig workers (Lang et al., 2023; Gong, 2025). In this study, emotional exhaustion therefore refers to delivery workers’ feeling of being emotionally tired, worn out, and mentally depleted when working under platform rhythms and algorithmic requirements.

Emotional feigning concerns the discrepancy between workers’ feelings and their outward display during service work. Emotional labor theory explains how workers regulate expression to meet role expectations and display rules (Grandey, 2000; Grandey and Sayre, 2019). Surface acting involves modifying outward expression without changing inner feelings and is associated with strain and less favorable work-related outcomes (Hülsheger and Schewe, 2011). In delivery work, workers may suppress irritation and maintain calmness while handling urgent prompts, customer complaints, or service recovery. The EFD items used here cover emotional suppression and calm continuation under work demands; Section 3.2 specifies the content of this proxy.

Exhaustion and feigning have different conceptual meanings. Emotional-labor frameworks distinguish display requirements and regulation strategies from strain outcomes (Grandey, 2000; Brotheridge and Grandey, 2002; Grandey and Gabriel, 2015). Exhaustion concerns depleted resources and is associated with poorer performance and citizenship behavior (Cropanzano et al., 2003); feigning concerns maintaining an outward display despite discrepant feelings (Grandey, 2003; Hülsheger and Schewe, 2011). A worker may report exhaustion without masking emotion in a particular encounter, or mask irritation while retaining energy. Treating them separately allows the analysis to examine their respective associations with minimum-only execution.

### 2.4 Theoretical foundations

Affective events theory, conservation of resources theory, and emotional labor theory guide the selection of associations examined in this study. Affective events theory directs attention to deadlines, prompts, and monitoring as work events with emotional relevance (Weiss and Cropanzano, 1996). Conservation of resources theory motivates examining depletion alongside demands and discretionary effort (Hobfoll, 1989). Emotional labor theory directs attention to outward display regulation during customer-facing work (Grandey, 2000; Grandey and Sayre, 2019). Together, they provide a basis for considering emotional exhaustion and emotional feigning alongside the control-related dimensions.

Conservation of resources theory proposes that people seek to retain valued resources and experience stress when those resources are threatened, lost, or insufficiently replenished (Hobfoll, 1989). Delivery work requires attention to time, rapid responses, changing task demands, and continued visibility. Workers reporting more of these demands may also report greater depletion, while workers reporting depletion may conserve effort beyond formal requirements. This perspective provides a basis for examining exhaustion as a correlate of both control-related experiences and minimum-only execution.

Emotional labor theory provides a complementary perspective on service workers’ outward displays (Grandey, 2000; Grandey and Sayre, 2019). Delivery workers interact with customers while their service is evaluated through ratings and platform metrics. They may report strong control-related demands while suppressing irritation or dissatisfaction to maintain an acceptable interaction. Such masking may also accompany reduced willingness to contribute beyond formal requirements. Emotional feigning therefore adds information about display regulation that is conceptually separate from resource depletion.

The relationship between exhaustion and display regulation can depend on the time scale examined. Resource-based research allows that workers with insufficient energy may rely more heavily on surface acting, with implications for later resource loss (Sayre et al., 2025). Other research links surface acting to subsequent strain, and episode-level studies allow reciprocal relationships (Grandey, 2003; Hülsheger et al., 2010; Hülsheger and Schewe, 2011; Xanthopoulou et al., 2018; Nesher Shoshan et al., 2023). These accounts suggest that exhaustion and display regulation may be related in more than one temporal ordering. Examining them separately allows the study to distinguish their associations with minimum-only execution.

### 2.5 Original aggregate-model hypotheses and dimension-level research questions

H1–H4 are the original hypotheses for the aggregate PAC model; their estimates are retained in Supplementary Appendix S11. H2–H4 refer to coefficient products in the single-wave data. The dimension-level research questions were introduced during peer review and guide a separate exploratory analysis; they do not constitute new tests of H1–H4.

The original H1 drew on the possibility that formal task completion and discretionary cooperation can diverge under intensive control. Platform research describes how monitoring, ratings, and sanction risks may constrain discretion while workers remain dependent on access to assignments (Rosenblat and Stark, 2016; Wood et al., 2019; Duggan et al., 2023; Wiener et al., 2023). Work-to-rule and low-visibility resistance research also shows that adherence to requirements can coexist with withholding additional effort (Hale and Borys, 2013; Galière, 2020; Alcadipani et al., 2018). This reasoning motivated the original positive association hypothesis between aggregate PAC and PC (H1).

*H1*. Perceived algorithmic control is positively associated with rule-bounded reduced discretionary effort (PC) among platform delivery workers.

The original H2 concerned exhaustion as a correlate linking aggregate PAC and PC. Research associates algorithmic control and surveillance with technostress, burnout, and exhaustion (Cram et al., 2022; Lang et al., 2023; Gong, 2025). Resource conservation provides a reason why depleted workers might limit discretionary contribution while maintaining required procedures (Hobfoll, 1989), consistent with observed relationships between exhaustion, performance, and citizenship behavior (Cropanzano et al., 2003). The corresponding statistical hypothesis concerned the product of the PAC–EEX and EEX–PC coefficients.

*H2*. In the specified aggregate PAC model, the product of the PAC–EEX and EEX–PC coefficients is positive.

The original H3 concerned display regulation as another correlate linking aggregate PAC and PC. Service workers may maintain an acceptable outward display while feeling irritated or dissatisfied, and emotional-labor research associates surface regulation with strain and work-related outcomes (Grandey, 2000; Brotheridge and Grandey, 2002; Grandey, 2003; Hülsheger and Schewe, 2011; Grandey and Sayre, 2019). In delivery work, this tension can coexist with continued formal task completion and reduced additional effort. H3 represented that proposition as the product of the PAC–EFD and EFD–PC coefficients.

*H3*. In the specified aggregate PAC model, the product of the PAC–EFD and EFD–PC coefficients is positive.

H4 retained the original exhaustion-to-feigning ordering, motivated by the possibility that depleted workers rely more heavily on surface acting (Sayre et al., 2025). The competing and reciprocal accounts discussed above remained theoretically plausible (Grandey, 2003; Hülsheger et al., 2010; Hülsheger and Schewe, 2011; Nesher Shoshan et al., 2023).

*H4*. In the specified aggregate PAC model, the product of the PAC–EEX, EEX–EFD, and EFD–PC coefficients is positive.

The exploratory dimension-level analysis addresses two research questions. RQ1 asks how TAC, RAC, and TRC are associated with EEX, EFD, and PC when entered jointly, with EEX additionally included in the EFD equation and both emotional variables included in the PC equation. RQ2 asks what direct, model-specific indirect, and total association patterns follow for each dimension. Figure 1 summarizes these three equations and their 12 conditional associations among concurrently measured constructs.

**Figure 1:**
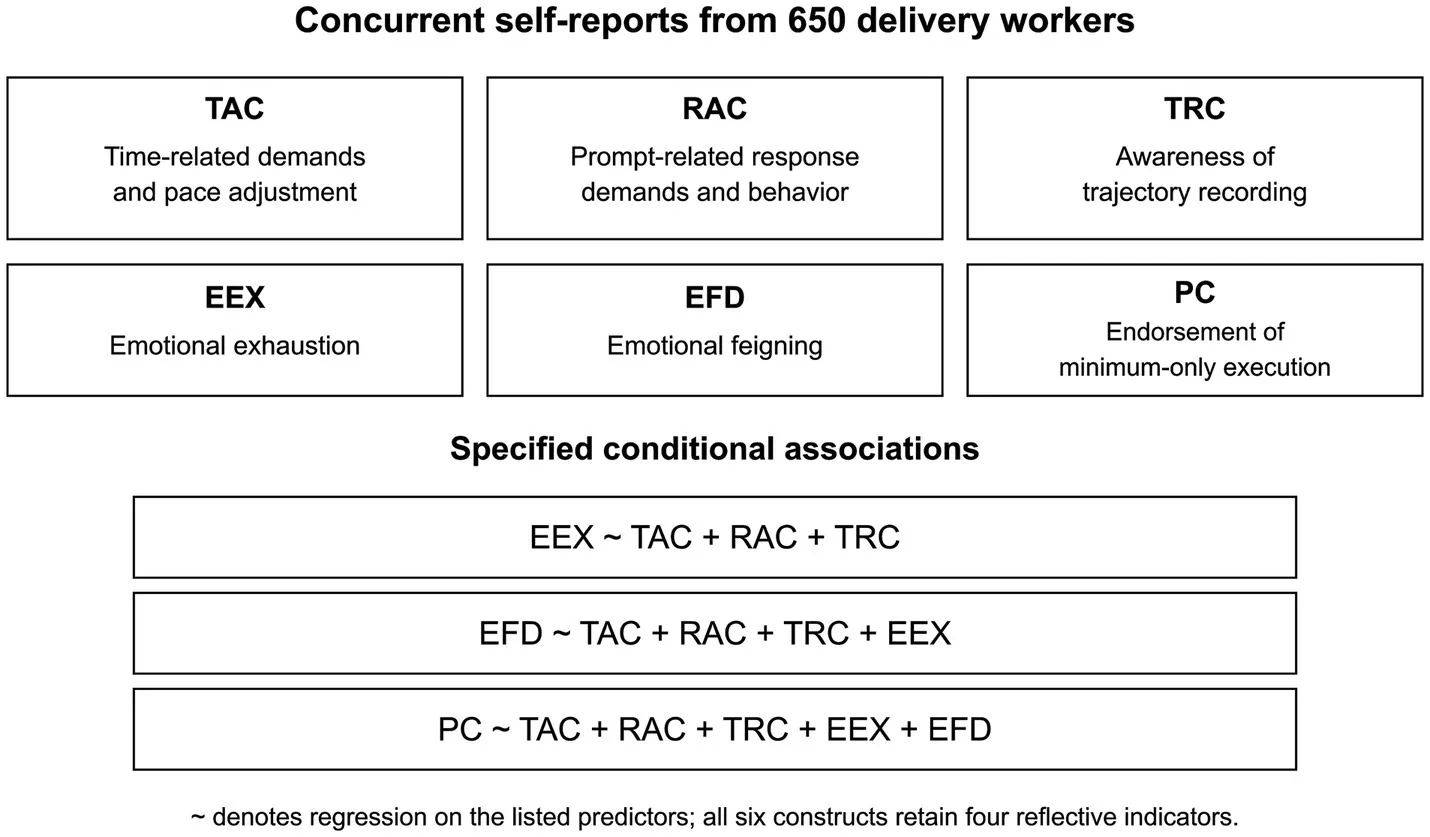
Cross-sectional association framework and specified equations. All six constructs were measured in the same survey wave. The tilde denotes regression on the variables listed to its right; the three equations specify 12 conditional associations. TAC, RAC, and TRC enter jointly, and each construct has four reflective indicators. The layout and coefficient products describe within-wave associations; they do not establish temporal order or causal mediation. PC denotes endorsement of minimum-only execution statements.

## 3 Materials and methods

### 3.1 Participants and procedure

This study used a single-wave, cross-sectional online survey. The questionnaire was open to platform delivery workers in China from May 18 to May 30, 2026. The research team first contacted a manager at a food-delivery station, explained the study, and obtained support for distributing the questionnaire. The manager circulated the survey link through nationwide WeChat networks used by food-delivery and express-delivery workers and asked managers in other regions to forward the invitation within their local work groups. Recruitment therefore followed a network-based group-broadcast process rather than a station-by-station sampling frame. Platform delivery work in China is often overlapping: some workers deliver part-time for more than one platform, and some combine food delivery with express or local instant delivery. The delivery-type and primary-platform categories in this study therefore describe respondents’ predominant self-reported work arrangements rather than mutually exclusive occupational memberships.

The questionnaire targeted workers who had engaged in food delivery, express delivery, or local instant delivery within the previous 3 months and whose work was organized through platform- or system-based dispatching and management. A total of 660 responses were initially obtained. Respondents were screened by three eligibility questions, an attention-check item, response-time diagnostics, and multivariate anomaly checks. After excluding abnormal responses, 650 valid questionnaires were retained for the main analysis. The final sample included delivery workers from food delivery, express delivery, local instant delivery, and mixed delivery backgrounds. Specifically, 244 respondents mainly engaged in food delivery (37.5%), 143 in express delivery (22.0%), 117 in local instant delivery (18.0%), and 146 in multiple delivery types (22.5%). In terms of primary platform, 217 respondents mainly used Meituan (33.4%), 118 Ele.me (18.2%), 75 SF Express (11.5%), 98 JD Daojia/Dada (15.1%), 50 FlashEx (7.7%), 39 STO/YTO/ZTO/Yunda/Cainiao-type express systems (6.0%), and 53 multiple platforms (8.2%). Regarding work mode, 299 respondents were full-time delivery workers (46.0%), 215 were part-time workers (33.1%), and 136 worked occasionally or temporarily (20.9%). Among all respondents, 474 were male (72.9%) and 176 were female (27.1%); 299 were aged 26–35 years (46.0%), and 159 were aged 36–45 years (24.5%).

The group-broadcast recruitment process provided no station identifiers, invitation denominators, or refusal counts and did not allow respondents to be assigned to unique stations. Station-specific and overall recruitment response rates are therefore unavailable. The 650 retained cases represented 98.5% of submitted questionnaires, an analytic retention rate after quality screening.

Eligibility was assessed with three questions: (1) whether the respondent was at least 18 years old; (2) whether the respondent had engaged in food delivery, express delivery, or local instant delivery during the previous 3 months; and (3) whether the respondent’s delivery work was accepted, dispatched, or managed mainly through a platform or system. Respondents were retained if they answered yes to the first and third questions and selected either “yes, every week” or “yes, but only occasionally” to the second. Among the 660 initially obtained responses, all respondents met these three eligibility criteria; 362 reported weekly delivery work and 298 reported occasional delivery work.

The survey protocol was reviewed and approved by the Ethics Committee of the School of Accounting, Tongling University (approval no. TLUIRB20260513001). Participation was voluntary and anonymous. Informed consent was obtained from participants and documented separately. The questionnaire landing page stated that the information would be used only for academic research, kept strictly confidential, and would not involve personally identifying information. Station managers had no access to individual responses.

### 3.2 Measures and data analysis method

The measures distinguish control-related experiences from emotional reports and minimum-only execution. TAC was informed by the pace and time-scarcity content of the Quantitative Workload Inventory (Spector and Jex, 1998), with item referents changed to platform time limits, reminders, system timing, continuous dispatching, and countdowns. Its items include both time-related requirements and reported pace adjustment. RAC was informed by workplace telepressure (Barber and Santuzzi, 2015); its administered items combine prompt-related response requirements with reports of checking, replying, and acting on notifications.

TRC was adapted from the tracking-and-evaluation dimension of perceived algorithmic control (Pei et al., 2021; see also Zhu et al., 2024). Its four administered items concern awareness that location, route changes, stops, or detours are recorded; they do not cover the source dimension’s work-attitude or completion-quality content. Supplementary Appendix S2 documents the source instruments and administered wording.

EEX was contextually adapted from the seven-item work-related burnout subscale of the Copenhagen Burnout Inventory (CBI; Kristensen et al., 2005). Four fatigue and exhaustion themes were redeveloped for platform pace and delivery work and administered using the survey’s seven-point agreement format. EEX therefore captures fatigue and exhaustion reports in this setting; its scores are not equivalent to the original CBI score or its 0–100 scoring procedure.

EFD was informed by the emotional dissonance scale in emotion-work research (Zapf et al., 1999). EFD1 describes suppressing emotions while continuing delivery work, EFD2 and EFD3 address suppressing dissatisfaction or holding back true emotions, and EFD4 describes remaining calm and continuing work. The block thus covers emotional suppression and calm continuation under work demands. Because remaining calm does not necessarily involve feigning an emotion, the score is a context-specific proxy with this content range, rather than a comprehensive measure of emotional feigning or surface acting.

PC was informed by the minimum-effort and procedure-following content of surface compliance (Hu et al., 2020). Its four rewritten items describe minimum-only execution within formal platform requirements. PC1 asks respondents to judge what delivery workers sometimes do, whereas PC2–PC4 concern their own conduct. PC thus captures endorsement of these statements, combining a judgment about delivery workers generally with personal reports.

Higher scores indicate stronger endorsement of minimum-only execution, while lower scores can reflect either additional contribution or failure to meet the stated requirement. The score does not separately measure basic compliance and additional contribution or distinguish effort among workers independently established to be equally compliant. The items do not assess intentions to punish, retaliate, or harm.

All six constructs used four items and a common seven-point endpoint-anchored agreement scale. The resulting scores are context-specific operationalizations, not metrically equivalent versions of the source instruments. Supplementary Appendix S2 documents item provenance and redevelopment; Supplementary Appendix S3 compares the PC items with definitions of adjacent constructs.

Table 1 reports the English translations of the 24 final-model items, their loadings, and construct-level reliability and convergent-validity statistics. Respondents used a seven-point scale labeled only at 1 (strongly disagree) and 7 (strongly agree). Supplementary Appendix S1 provides the complete Chinese questionnaire and the English translation prepared for reporting, including eligibility questions and screening rules, work-background and demographic questions, the attention check, and candidate items excluded from the final model.

**Table 1:** Measurement items and reflective measurement statistics.

Panel A. Administered item reporting translations and native PLS loadings			
Construct	Item	English reporting translation	Loading
TAC	TAC1	The platform uses time limits and reminders to set the pace of delivery work.	0.7841
TAC	TAC2	While making deliveries, I speed up to keep pace with the system’s timing.	0.7952
TAC	TAC3	While making deliveries, I speed up when orders are assigned continuously.	0.8111
TAC	TAC4	While making deliveries, I adjust my pace according to the countdown.	0.7786
RAC	RAC1	The system uses order-assignment notifications and messages to prompt delivery workers to respond promptly.	0.8055
RAC	RAC2	While making deliveries, I quickly check order-assignment notifications.	0.7845
RAC	RAC3	While making deliveries, I reply to customer messages as soon as possible.	0.7983
RAC	RAC4	While making deliveries, I act on system prompts immediately.	0.7446
TRC	TRC1	The system uses location and route information to record the delivery process.	0.8142
TRC	TRC2	While making deliveries, I feel that my location is continuously recorded.	0.8146
TRC	TRC3	While making deliveries, I know that changes in my route are recorded.	0.8179
TRC	TRC4	While making deliveries, I know that stops or detours are recorded.	0.7901
EEX	EEX1	When the platform maintains a tight work pace, delivery workers are likely to feel emotionally tired.	0.7926
EEX	EEX2	When working at the platform’s pace, I feel emotionally tired.	0.7442
EEX	EEX3	When working at the platform’s pace, I feel drained.	0.7885
EEX	EEX4	When working at the platform’s pace, I feel emotionally worn out.	0.7757
EFD	EFD1	In platform work, delivery workers sometimes need to suppress their emotions and continue making deliveries.	0.8015
EFD	EFD2	When facing work demands, I suppress my dissatisfaction.	0.8252
EFD	EFD3	When facing work demands, I hold back my true emotions.	0.8089
EFD	EFD4	When facing work demands, I remain calm and continue making deliveries.	0.8381
PC	PC1	Within platform rules, delivery workers sometimes perform only the prescribed actions.	0.7555
PC	PC2	When carrying out platform requirements, I sometimes perform only the prescribed actions.	0.7451
PC	PC3	When carrying out platform requirements, I sometimes do only what is basically required.	0.7884
PC	PC4	When carrying out platform requirements, I sometimes simply complete the required procedure.	0.7628

Panel B. Construct statistics				
Construct	Standardized alpha	rho_A	rho_C	AVE
TAC	0.8024	0.8038	0.8709	0.6278
RAC	0.7901	0.7915	0.8641	0.6140
TRC	0.8244	0.8248	0.8836	0.6549
EEX	0.7798	0.7845	0.8578	0.6014
EFD	0.8359	0.8374	0.8903	0.6700
PC	0.7607	0.7610	0.8479	0.5824

*N* = 650; four reflective indicators per construct. Item wording is preserved from the English reporting translations of the administered Chinese questionnaire in S1. Values refer to the joint dimension model. Construct definitions and measurement scope are described in Section 3.2. rho_C is composite reliability.

Before data collection, the authors developed the Chinese items through source review, semantic rendering, and contextual rewriting for platform delivery work, documented in an item-development workbook (S2). No independent back-translation or formal discrepancy-reconciliation record was retained. The English wording in Supplementary Appendix S1 was prepared for reporting during revision.

The primary analysis enters TAC, RAC, and TRC jointly as separate first-order constructs, with the original four reflective indicators retained in each of TAC, RAC, TRC, EEX, EFD, and PC. The common attributes posited within the control-related blocks are experienced time pacing, prompt responsiveness, and recording awareness, respectively. The reflective specification treats each block’s reports as manifestations expected to vary with its domain-level experience; this is a substantive modeling assumption, not evidence that requirements and workers’ responses have been separately identified.

The joint specification was introduced during revision after examining whether a single higher-order PAC representation obscured differences among the three domains. Items and paths were retained without selection based on the new estimates. No new directional dimension-level hypotheses were formulated after examining the data. Supplementary Appendix S11 preserves the original disjoint two-stage reflective higher-order model and its H1–H4 results.

The three structural equations are EEX ~ TAC + RAC + TRC; EFD ~ TAC + RAC + TRC + EEX; and PC ~ TAC + RAC + TRC + EEX + EFD. A tilde denotes regression on the listed predictors. The resulting 12 coefficients describe contemporaneous conditional associations; their products are reported as model-specific indirect associations and do not establish temporal mediation. For each control-related dimension, the reported products follow dimension–EEX–PC, dimension–EFD–PC, and dimension–EEX–EFD–PC. Their sum is the total indirect association; adding the dimension–PC coefficient gives its total association within this model. That quantity is distinct from the dimension’s zero-order correlation with PC.

The model was estimated in SmartPLS 4.1.1.8 using ordinary PLS-SEM with path weighting and standardized results. All six measurement blocks were reflective. The coefficients describe conditional associations among weighted summaries of the administered item responses, without correction for common-factor measurement-error attenuation. Algorithm settings and convergence records are provided in S12.

Measurement assessment considered loadings, standardized Cronbach’s alpha, rho_A, composite reliability (rho_C), average variance extracted (AVE), heterotrait–monotrait ratios (HTMT), and variance inflation factors (VIF), following the reporting framework of Hair et al. (2019, 2022) and Henseler et al. (2016).

AI assistance with earlier interface operations and model setup is described in the Generative AI statement.

The complete bootstrap procedure produced 10,000 saved parameter draws by resampling individual respondents. Inference uses two-tailed tests at 0.05 and the prespecified 95% percentile confidence intervals. Dependence among respondents from shared recruitment sources was not assessed, so the intervals reflect individual-level resampling. Bootstrap settings and export-record limitations are documented in S12.

Model-fit assessment reports SRMR alongside the adapted Bollen–Stine bootstrap reference bounds for SRMR, d_ULS, and d_G. These fit bounds are distinct from the parameter confidence intervals. Supplementary Appendix S12 documents the settings, source locations, complete parameter tables, and separate approximate- and exact-fit evidence.

A revision-stage CFA diagnostic examined the information lost when TAC, RAC, and TRC were represented by a common PAC factor. It compared a six-correlated-factor model with a model channeling the three domains’ external relations through that shared factor. Both models retained the same 650 observations, 24 indicators, and uncorrelated item residuals. Seven-point items were treated as approximately continuous and estimated with robust maximum likelihood (MLR) in lavaan 0.7–2 under R 4.6.1. The six additional covariance restrictions were tested with the Satorra 2000 scaled-and-shifted difference procedure. This comparison concerns two MLR models; it is separate from the PLS estimation (S11).

Supplementary Appendix S11 indexes the retained historical analyses and their original methods. S6 reports the unrotated principal-component diagnostic and a covariance-based sensitivity analysis that adds an orthogonal unmeasured common method factor to six correlated substantive factors. This analysis was not used to correct the revised PLS estimates, and its assumptions limit what it can establish about shared reporting variance (Podsakoff et al., 2003; Richardson et al., 2009; Chin et al., 2012).

The earlier principal-component-score and ordinary-least-squares dimension analyses are retained in S5. S7 reports aggregate PAC equations adjusted for work mode, age group, gender, delivery tenure, and daily working hours, together with work-mode subgroup analyses. Their settings and uncertainty estimates remain attached to those historical score models; they do not provide control-adjusted coefficients for the revised dimension-level PLS model.

Supplementary Appendix S8 examines response-order patterns using the preserved questionnaire sequence identifier, because submission timestamps and a recruitment denominator were unavailable. First-versus-last-quartile and tertile comparisons used Welch tests and Hedges g for focal constructs and chi-square tests and Cramer’s V for demographic categories. Such comparisons can detect gradients among submitted questionnaires but cannot establish representativeness of people outside the recruitment networks (Armstrong and Overton, 1977). Published workforce figures serve as descriptive benchmarks with different coverage and work-mode definitions.

Supplementary Appendices S9 and S10 compare alternative orderings of the original four-construct aggregate model using standardized principal-component scores and ordinary least squares. S9 places PC before PAC and the emotional variables; S10 reverses EEX–EFD while retaining the other direct relations. Within each comparison, the fully recursive score models are covariance-equivalent and cannot identify temporal direction. Their detailed results and the original IPMA (Ringle and Sarstedt, 2016) are indexed in S11.

## 4 Results

### 4.1 Measurement model assessment

In the revised dimension-level PLS model, the 24 loadings ranged from 0.7442 to 0.8381. Standardized Cronbach’s alpha ranged from 0.7607 to 0.8359, rho_A from 0.7610 to 0.8374, and rho_C from 0.8479 to 0.8903. AVE ranged from 0.5824 to 0.6700. These results are consistent with internal consistency and convergent validity under the retained reflective specification (Fornell and Larcker, 1981; Hair et al., 2019, 2022). Table 1 reports the item loadings and construct-level statistics from this model.

Across the 24 items, the means ranged from 4.635 to 6.026 and sample variances from 0.845 to 1.517. Each item’s own-construct loading exceeded its cross-loadings in the revised PLS model; the smallest margin was 0.3258 (S12).

### 4.2 Discriminant validity

The 15 HTMT ratios among the six constructs ranged from 0.3124 to 0.6827. The largest was TRC–RAC (0.6827), whose 95% percentile interval was [0.6192, 0.7417]; the largest upper confidence limit across all pairs was 0.7417 (Table 2). These values support discrimination among the six measured constructs under the HTMT diagnostic (Henseler et al., 2015). Item-content boundaries are addressed in the measurement descriptions and Supplementary Appendices S2 and S3.

**Table 2:** HTMT ratios among the six measured constructs.

Construct pair	HTMT	95% percentile CI
EFD—EEX	0.3124	[0.2290, 0.3952]
PC—EEX	0.4885	[0.4061, 0.5656]
PC—EFD	0.5145	[0.4369, 0.5903]
RAC—EEX	0.4200	[0.3343, 0.5029]
RAC—EFD	0.4863	[0.4063, 0.5622]
RAC—PC	0.5808	[0.5079, 0.6515]
TAC—EEX	0.3888	[0.2998, 0.4752]
TAC—EFD	0.5193	[0.4435, 0.5920]
TAC—PC	0.6572	[0.5900, 0.7205]
TAC—RAC	0.5795	[0.5095, 0.6457]
TRC—EEX	0.6058	[0.5359, 0.6721]
TRC—EFD	0.5135	[0.4417, 0.5820]
TRC—PC	0.3958	[0.3128, 0.4747]
TRC—RAC	0.6827	[0.6192, 0.7417]
TRC—TAC	0.6127	[0.5425, 0.6773]

The upper limit was below 0.85 for every pair. This diagnostic concerns the measured constructs and does not establish distinctiveness from unmeasured adjacent constructs.

### 4.3 Collinearity and model fit

The largest outer and inner VIF values were 1.9591 and 1.8939, respectively. Estimated and saturated SRMR were both 0.0492, below the 0.08 approximate-fit reference. In the complete bootstrap, however, SRMR exceeded its HI99 of 0.0428 and d_ULS (0.7274) exceeded its HI99 of 0.5487, while d_G (0.219479) was below its HI95 of 0.219742. The model therefore met the approximate SRMR reference but did not receive uniform support from the bootstrap discrepancy criteria (Table 3).

**Table 3:** Revised structural model and fit assessment.

Panel A. Explained variance			
Outcome	*R^2^*	Adjusted *R^2^*	95% percentile CI for *R^2^*
EEX	0.2478	0.2443	[0.1970, 0.3108]
EFD	0.2616	0.2570	[0.2124, 0.3236]
PC	0.3924	0.3876	[0.3424, 0.4540]

Panel B. Conditional direct associations					
Association	Beta	Bootstrap SD	*p*	95% percentile CI	Inner VIF
EEX → EFD	0.0257	0.0375	0.494	[−0.0476, 0.0999]	1.3294
EEX → PC	0.2345	0.0344	<0.001	[0.1681, 0.3024]	1.3303
EFD → PC	0.1888	0.0372	<0.001	[0.1152, 0.2609]	1.3542
RAC → EEX	0.0706	0.0425	0.097	[−0.0147, 0.1521]	1.5401
RAC → EFD	0.1636	0.0424	<0.001	[0.0797, 0.2464]	1.5467
RAC → PC	0.2423	0.0387	<0.001	[0.1671, 0.3184]	1.5829
TAC → EEX	0.0752	0.0400	0.060	[−0.0021, 0.1559]	1.4260
TAC → EFD	0.2410	0.0402	<0.001	[0.1621, 0.3175]	1.4336
TAC → PC	0.3392	0.0372	<0.001	[0.2652, 0.4128]	1.5122
TRC → EEX	0.4115	0.0406	<0.001	[0.3314, 0.4890]	1.6122
TRC → EFD	0.2045	0.0452	<0.001	[0.1172, 0.2943]	1.8373
TRC → PC	−0.1834	0.0429	<0.001	[−0.2686, −0.1003]	1.8939

Panel C. Approximate and bootstrap fit evidence					
Model	Criterion	Original value	HI95	HI99	Comparison
Saturated	SRMR	0.049243	0.041823	0.042769	Above HI99
Estimated	SRMR	0.049243	0.041823	0.042769	Above HI99
Saturated	d_ULS	0.727447	0.524749	0.548747	Above HI99
Estimated	d_ULS	0.727447	0.524749	0.548747	Above HI99
Saturated	d_G	0.219479	0.219742	0.225452	Below HI95
Estimated	d_G	0.219479	0.219742	0.225452	Below HI95

Native ordinary PLS, *N* = 650, 10,000 saved bootstrap parameter draws. Parameter intervals are two-tailed 95% percentile intervals. HI95 and HI99 are one-sided reference quantiles from the adapted Bollen–Stine model-fit bootstrap, not parameter confidence intervals. Approximate SRMR below 0.08 coexists with SRMR and d_ULS values above HI99; the fit diagnostics do not uniformly support the specification. No causal interpretation is assigned to the software path directions.

### 4.4 Structural model assessment

The revised model explained 24.78% of variance in EEX, 26.16% in EFD, and 39.24% in PC. Conditional on the other two control-related dimensions, TRC was positively associated with EEX (beta = 0.4115, 95% CI [0.3314, 0.4890]). The TAC–EEX and RAC–EEX intervals included zero (beta = 0.0752, *p* = 0.060, and beta = 0.0706, *p* = 0.097, respectively). In the EFD equation, TAC, RAC, and TRC were positively associated with EFD (beta = 0.2410, 0.1636, and 0.2045, respectively), whereas EEX–EFD was small and its interval included zero (beta = 0.0257, 95% CI [−0.0476, 0.0999], *p* = 0.494).

In the PC equation, TAC (beta = 0.3392, 95% CI [0.2652, 0.4128]) and RAC (beta = 0.2423, 95% CI [0.1671, 0.3184]) had positive conditional associations. TRC had a negative conditional coefficient (beta = −0.1834, 95% CI [−0.2686, −0.1003]). EEX and EFD were each positively associated with PC (beta = 0.2345 and 0.1888, respectively). All five PC coefficients had *p* < 0.001. Table 3 reports the full equations, including the nonsignificant coefficients. Differences in coefficient magnitudes are descriptive; no between-dimension coefficient contrast was estimated.

### 4.5 Model-specific indirect associations

Of the 13 specific indirect associations defined by the equations, four had 95% percentile intervals excluding zero (Table 4). The products through EFD were positive for TAC (0.0455, 95% CI [0.0252, 0.0696]), RAC (0.0309, [0.0137, 0.0517]), and TRC (0.0386, [0.0181, 0.0648]). The TRC–EEX–PC product was also positive (0.0965, [0.0647, 0.1329]). The corresponding TAC–EEX–PC product was 0.0176, but its percentile interval included zero [−0.0005, 0.0390] (*p* = 0.078); the RAC–EEX–PC interval also included zero.

**Table 4:** Specific indirect associations in the revised model.

Specified product	Estimate	Bootstrap SD	*p*	95% percentile CI
EEX → EFD → PC	0.0048	0.0073	0.509	[−0.0089, 0.0201]
RAC → EEX → EFD	0.0018	0.0033	0.587	[−0.0038, 0.0097]
RAC → EFD → PC	0.0309	0.0098	0.002	[0.0137, 0.0517]
RAC → EEX → PC	0.0166	0.0102	0.104	[−0.0035, 0.0371]
TAC → EEX → EFD	0.0019	0.0033	0.561	[−0.0042, 0.0094]
TAC → EFD → PC	0.0455	0.0115	<0.001	[0.0252, 0.0696]
TAC → EEX → PC	0.0176	0.0100	0.078	[−0.0005, 0.0390]
TRC → EEX → EFD	0.0106	0.0157	0.500	[−0.0196, 0.0419]
TRC → EFD → PC	0.0386	0.0121	0.001	[0.0181, 0.0648]
TRC → EEX → PC	0.0965	0.0175	<0.001	[0.0647, 0.1329]
TRC → EEX → EFD → PC	0.0020	0.0031	0.515	[−0.0037, 0.0085]
RAC → EEX → EFD → PC	0.0003	0.0006	0.595	[−0.0007, 0.0019]
TAC → EEX → EFD → PC	0.0004	0.0006	0.575	[−0.0008, 0.0019]

All 13 coefficient products implied by the 12 specified paths are reported, including nine dimension-to-PC products; four intervals exclude zero. Inference uses the prespecified percentile intervals. For TAC–EEX–PC, *p* = 0.078 and the unrounded lower endpoint is −0.00047095; the exported bias-corrected interval is not used for inference. All three dimension–EEX–EFD–PC intervals include zero. These estimates describe associations within one survey wave, leaving temporal ordering unresolved.

The products involving both EEX and EFD were 0.0004 for TAC, 0.0003 for RAC, and 0.0020 for TRC, and all three intervals included zero. These cross-sectional coefficient products leave the temporal relationship between EEX and EFD unresolved.

### 4.6 Direct, indirect, and total associations with PC

TAC and RAC had positive total associations with PC in the specified model (0.4027 and 0.2901, respectively; both *p* < 0.001). TRC combined a negative direct coefficient (−0.1834) with a positive total indirect association (0.1371, 95% CI [0.0996, 0.1797]); its total association was −0.0463, with a 95% interval of [−0.1253, 0.0320] and *p* = 0.241 (Table 5). The native PLS construct-score zero-order TRC–PC correlation was positive (*r* = 0.3145). The zero-order correlation and the conditional model decomposition describe different quantities and are not interchangeable.

**Table 5:** Direct, total indirect, and total associations with PC.

Dimension	Component	Estimate	*p*	95% percentile CI
TAC	Direct	0.3392	<0.001	[0.2652, 0.4128]
TAC	Total indirect	0.0635	<0.001	[0.0355, 0.0954]
TAC	Total	0.4027	<0.001	[0.3308, 0.4743]
RAC	Direct	0.2423	<0.001	[0.1671, 0.3184]
RAC	Total indirect	0.0478	<0.001	[0.0215, 0.0762]
RAC	Total	0.2901	<0.001	[0.2135, 0.3653]
TRC	Direct	−0.1834	<0.001	[−0.2686, −0.1003]
TRC	Total indirect	0.1371	<0.001	[0.0996, 0.1797]
TRC	Total	−0.0463	0.241	[−0.1253, 0.0320]

Total indirect is the sum of the three dimension-to-PC products; total is direct plus total indirect. The total quantity is conditional on the specified joint-dimension structure and is not the zero-order correlation. The native TRC–PC construct-score correlation is 0.31446121. TRC has opposite-signed direct and total indirect components, but its total interval includes zero.

A separate historical PCA-plus-OLS reconstruction showed the same sign pattern. In its fixed-score regressions, the TRC coefficient changed sign when TAC and RAC were included, before EEX and EFD were added (S5; indexed in S11). The revised PLS total association remained compatible with zero (Table 5).

### 4.7 Assessment of the common PAC representation

In the MLR diagnostic, the six-correlated-factor model had robust CFI = 1.000, robust RMSEA = 0.0000, and SRMR = 0.0227. The common-PAC model retained good approximate fit (robust CFI = 0.9884, robust RMSEA = 0.0205, SRMR = 0.0364), but the six added restrictions produced a robust difference of chi-square (6) = 102.495 (*p* < 0.001). The largest localized discrepancies concerned TAC–PC, TRC–PC, and TRC–EEX. The common representation therefore lost information about the pattern of external associations even though its higher-order loadings remained positive and its overall approximate fit was good (S11).

Together with the item-content differences, the localized discrepancies support reporting TAC, RAC, and TRC separately because aggregation obscured their external associations, particularly TAC–PC, TRC–PC, and TRC–EEX. This information loss is compatible with shared variation among the three domains.

### 4.8 Retained supplementary analyses

The original aggregate PAC analysis supported H1, H2, and H3 but did not support H4 (S11). The exploratory dimension-level questions are reported separately from these original hypothesis tests.

For the original four-construct score representation, S9 and S10 compared full recursive orderings with identical reconstructed stage-2 SRMR (0.073), log-likelihood, AIC, and BIC. The EFD-before-EEX coefficient product also had an interval including zero. These historical PCA-score comparisons leave temporal ordering unresolved (Henley et al., 2006; see S11).

### 4.9 Common method variance diagnostics

The unrotated principal-component analysis of the final 650 cases yielded five components with eigenvalues greater than one. The first accounted for 32.186% of total item variance, and the five together accounted for 59.519%. This descriptive finding does not establish the absence of common method variance (Supplementary Appendix S6).

The earlier correlated six-factor CFA gave chi-square (237) = 203.698 and SRMR = 0.024. Adding an orthogonal common method factor gave chi-square (213) = 162.867 and SRMR = 0.019; the difference was 40.831 with 24 additional parameters (*p* = 0.017), although AIC and BIC did not favor the more complex model. Average substantive, method, and residual variance were 44.557, 6.429, and 49.015%, respectively. Nine method loadings were significant at *p* < 0.05, with method variance varying across construct blocks. These results indicate heterogeneous method-related variance (S6); the revised dimension-level PLS coefficients remain unadjusted for this variance.

### 4.10 Non-response and sample-selection diagnostics

The first and last quartiles each contained 163 retained questionnaires. No focal construct comparison reached *p* < 0.05 (all *p* > = 0.120; maximum absolute Hedges *g* = 0.172), and gender, age group, and work mode did not differ significantly. The tertile analysis gave the same broad pattern (maximum absolute *g* = 0.180). These diagnostics did not reveal a strong response-order gradient among submitted questionnaires. They cannot assess workers who did not view the invitation or chose not to respond. Supplementary Appendix S8 retains the workforce benchmarks with their coverage and definition differences.

## 5 Discussion

### 5.1 Summary of key findings

The three control-related dimensions showed distinct patterns of association with minimum-only execution endorsement. With the other two dimensions and both emotional variables included, temporal demands and pace adjustment (TAC) and prompt-related response demands and behavior (RAC) had positive PC coefficients (*β* = 0.3392 and 0.2423), while trajectory-recording awareness (TRC) had a negative coefficient (*β* = −0.1834). TRC was positively associated with exhaustion (*β* = 0.4115), whereas the TAC–EEX and RAC–EEX intervals included zero. All three dimensions were positively associated with feigning after EEX was included in that equation. These findings identify time, prompt-response, and recording experiences as distinct components to examine in accounts of minimum-only execution endorsement.

TRC illustrates the importance of distinguishing marginal and conditional associations. Its native PLS construct-score correlation with PC was positive (r = 0.3145), while its coefficient in the joint PC equation was negative. The negative direct coefficient (−0.1834) and positive total indirect association (0.1371) sum to a total association of −0.0463, with a 95% percentile interval of [−0.1253, 0.0320]. Thus, the sign of the direct coefficient does not establish an overall protective relationship between trajectory recording and minimum-only execution. It describes the remaining association under the specified adjustment, and the model total is not a decomposition of the positive zero-order correlation.

Exhaustion and feigning were each positively associated with PC in the joint equation (*β* = 0.2345 and 0.1888). The coefficient products through EFD were positive for TAC, RAC, and TRC, and the TRC–EEX–PC product was also positive, with all four percentile intervals excluding zero. The TAC–EEX–PC and RAC–EEX–PC intervals included zero, as did all products involving both EEX and EFD. Together, these results make emotional masking a relevant part of the account of minimum-only execution endorsement across the three control-related dimensions. The original aggregate H4 remained unsupported in its own analysis, reported in Supplementary Appendix S11.

### 5.2 Theoretical implications

This study extends research on workers’ responses to algorithmic control by distinguishing the reported control experiences associated with minimum-only execution endorsement. Research on platform consent, coping, voice, and resistance has established the variety of these responses (Rosenblat and Stark, 2016; Galière, 2020; Kellogg et al., 2020; Cram et al., 2022; Wiener et al., 2023; Yu et al., 2022). In this sample, the common-PAC diagnostic identified its largest localized discrepancies for TAC–PC, TRC–PC, and TRC–EEX, while the joint model showed positive TAC–PC and RAC–PC coefficients, a negative conditional TRC–PC coefficient, and a positive TRC–EEX association. Treating control as a single predictor therefore obscured information relevant to both exhaustion and minimum-only execution endorsement. These findings shift attention from an overall perception of control to the content of workers’ time-related, prompt-response, and recording experiences.

The study also contributes to emotional-labor accounts of platform work by examining resource depletion and emotional masking separately in relation to minimum-only execution endorsement. Prior research connects algorithmic management to work intensity, technostress, autonomy, legitimacy judgments, and worker responses (Wood et al., 2019; Cram et al., 2022; Wiener et al., 2023; Parent-Rocheleau and Parker, 2022), while emotional-labor research distinguishes regulation strategies from strain outcomes (Brotheridge and Grandey, 2002; Grandey, 2003; Grandey and Gabriel, 2015). In the joint model, all three control-related dimensions were associated with EFD, whereas only the TRC–EEX coefficient had an interval excluding zero; both emotional scores were positively associated with PC. Emotional masking remained positively associated with minimum-only execution endorsement when exhaustion was included in the model. This finding broadens the emotional account of minimum-only execution endorsement in platform delivery work to include workers’ regulation of outward emotional displays alongside their experience of resource depletion.

### 5.3 Practical implications

Platform operators could supplement formal task-completion measures with assessments of discretionary contributions. Endorsement of minimum-only execution statements points to the value of examining contributions that completion, lateness, complaints, and rule-violation records may not capture. Proactive communication, service recovery, and voluntary problem solving are possible areas for prospective assessment. Recording these contributions together with basic task completion and opportunities to contribute would allow operators to examine variation in cooperation among workers facing comparable requirements.

Managers could review deadlines and pacing, prompt-related response demands, and trajectory recording as distinct features of delivery work. Trajectory recording warrants assessment alongside exhaustion and minimum-only execution endorsement because its direct and indirect components have opposite signs and its total interval includes zero. Prior research also links algorithmic management to workload, autonomy, legitimacy judgments, and worker responses (Parent-Rocheleau and Parker, 2022; Cram et al., 2022; Wiener et al., 2023). An operational review could examine whether deadlines, reminders, and route alerts accommodate congestion, merchant delay, elevator waiting, and customer communication. Exception handling and access to human review could be evaluated prospectively using workers’ experience and service outcomes alongside task completion.

Worker support could distinguish resource depletion from the demand to maintain an acceptable outward display. Recovery opportunities, pacing, and workload buffers are relevant areas for examining exhaustion, while complaint handling, customer-conflict support, and clear service expectations are relevant to emotional masking. The positive associations of both emotional variables with minimum-only execution endorsement make their separate assessment useful for identifying support needs. Prospective evaluations of these support options could track workers’ emotional experiences and discretionary contributions alongside basic task completion.

### 5.4 Interpreting the exhaustion–feigning association

In the joint model, the EEX–EFD coefficient was 0.0257 (95% percentile CI [−0.0476, 0.0999], *p* = 0.494), and each dimension–EEX–EFD–PC product had an interval including zero. The measured between-person associations therefore provided no support for the specified exhaustion–feigning link or the serial products. Their temporal relationship remains open for investigation with repeated measurements.

Longitudinal research could clarify how exhaustion and emotional masking relate across work episodes. Emotional-labor research distinguishes depleted states from display-regulation strategies and links surface acting to later strain (Grandey and Gabriel, 2015; Grandey, 2003; Hülsheger et al., 2010; Hülsheger and Schewe, 2011; Xanthopoulou et al., 2018). Resource-based and episode-level accounts also allow exhaustion to precede further masking or the two experiences to reinforce one another (Sayre et al., 2025; Nesher Shoshan et al., 2023). Diary or repeated-episode measurements could examine these possibilities at an appropriate temporal scale, while separating the context-specific EFD measure used here from the broader surface-acting construct.

### 5.5 Limitations and future research

The single-wave design does not identify temporal precedence or causal direction. Workers who endorse minimum-only execution may experience closer oversight or interpret platform requirements differently, and shared unmeasured conditions may also account for the associations. The coefficients, products, and totals summarize relationships among the measured scores; cross-sectional mediation estimates can differ from longitudinal processes, and alternative orderings can reproduce the same covariance information (Maxwell and Cole, 2007; Henley et al., 2006). Repeated measurements, plausibly exogenous platform-rule changes, and linked behavioral records could provide evidence about temporal relationships.

All focal constructs were reported by the same workers in one questionnaire, without a marker variable, temporal separation, or an independently sourced behavioral outcome. Common method variance therefore remains a limitation. The retained diagnostics cover the same 24 indicators, but their method-adjusted structural results concern the original aggregate PAC model (S6; indexed in S11). Social desirability, recall, negative affectivity, and subjective interpretation of requirements may still contribute to the revised dimension-level associations. Combining worker reports with independent records, observations, or repeated measurements would help assess these influences.

Measurement content remains a competing explanation for the different associations of TAC, RAC, and TRC: the first two blocks combine perceived requirements with reported responses, whereas TRC emphasizes recording awareness. Separating them does not isolate external control mechanisms, and the emotion measures retain the context-specific coverage described in Section 3.2. Without dispatch logs, assigned deadlines, penalty records, independent workload measures, or a human-dispatcher comparison group, the study cannot isolate objective platform actions or associations unique to artificial intelligence. Future measurement work should distinguish demands, responses, and recording awareness and assess automated sanctions and access to human review directly.

The PC score combines a judgment about delivery workers generally with three first-person reports. It does not independently establish equal basic compliance, comparable opportunities to contribute, or the amount of discretionary effort. The measurement implications and ambiguity of lower scores are described in Section 3.2 and S3. Future work should measure compliance, contribution opportunities, and effort separately. Direct comparisons with adjacent measures in the same sample are needed to assess empirical distinctiveness, with motive measured when it is part of the research question.

The sample comprises platform delivery workers in China recruited through station-manager and delivery-worker WeChat networks. Invitation, refusal, and station-source counts were unavailable, and the 650/660 ratio denotes retention among submitted questionnaires rather than a recruitment response rate. Cross-platform work and overlapping delivery arrangements also prevented mutually exclusive recruitment strata, while any similarity associated with shared recruitment or work contexts could not be quantified. The findings apply most directly to workers reached through comparable networks; replication with documented recruitment sources and broader sampling would help assess their generality.

The revised joint model was introduced during peer review using the original sample and items. Its questions and dimension-specific patterns therefore require independent replication, and no formal tests comparing coefficients across dimensions were conducted. The model’s approximate SRMR was below 0.08, but the observed SRMR and d_ULS values exceeded their bootstrap HI99 bounds, whereas d_G was below HI95; the fit evidence does not uniformly support exact fit. Perceived fairness, income uncertainty, customer conflict, and station support were not included in the model and could help account for the associations. Studies that measure these conditions alongside repeated emotional and behavioral observations could refine the account.

Testing whether the revised dimension-level associations differ across work arrangements would require prespecified group hypotheses, measurement-invariance assessment, and adequate samples within each group.

## 6 Conclusion

Among 650 surveyed delivery workers, temporal and response-related experiences were positively associated with endorsement of minimum-only execution statements after the other dimensions and emotional variables were included. Trajectory-recording awareness showed a negative conditional direct coefficient and positive indirect associations, while its total association with the outcome was not statistically distinguishable from zero. Exhaustion and feigning were each positively associated with stronger endorsement. Together, the common-PAC diagnostic and joint model show why time-related demands and pace adjustment, prompt-related demands and responses, and recording awareness merit separate attention when studying minimum-only execution endorsement. For platform management, the findings point to assessing these features separately while tracking workers’ emotional experiences and contributions beyond routine task completion.

## Data Availability

The original contributions presented in the study are included in the article/Supplementary material, further inquiries can be directed to the corresponding author/s.
