# Supplementary material for: Dimensions of perceived algorithmic control and endorsement of minimum-only execution in platform delivery work
Source: Front Psychol. 2026 Oct 7;17:1937810. doi: 10.3389/fpsyg.2026.1937810 (PMC13683074; doi:10.3389/fpsyg.2026.1937810)
Supplement: Supplementary file 1 [file Data_Sheet_1.ZIP › Supplementary Appendix S1 Full Questionnaire.docx]

Supplementary Appendix S1. Full Questionnaire Administered to Respondents

*Original language: Chinese | English translation supplied for reader inspection*

**Reporting boundary:** The Chinese text below is reproduced from the administered Wenjuanxing questionnaire. The English text was prepared for this revision to allow readers to inspect item content. It is not presented as evidence that a contemporaneous translation/back-translation procedure was conducted.

**Analytic transparency:** Items marked “Not used in final model” were administered in the survey but were not included in the model reported in the submitted manuscript. Their inclusion here is solely to disclose the complete instrument.

# S1. Survey title and introductory text

| **Code** | **中文原文** | **English translation** | **Analytic status** |
| --- | --- | --- | --- |
| Title | 一个关于配送工作体验的小调查 | A Short Survey on Delivery Work Experience | Survey text |
| Intro | 您好！本问卷用于了解外卖、快递等配送人员在平台接单、送单过程中的工作感受与行为反应。问卷匿名填写，所有信息仅用于学术研究，并将严格保密，不涉及个人身份识别。如果你参与作答，则视为您同意参与本次调研。预计作答约3—5分钟。感谢您在忙碌中支持本次调研！ | Hello. This questionnaire is intended to understand the work experiences and behavioral responses of food-delivery, express-delivery, and other delivery workers when accepting and completing platform-based delivery tasks. The questionnaire is anonymous. All information will be used only for academic research, kept strictly confidential, and will not involve personally identifying information. By participating in the questionnaire, you are deemed to have consented to take part in this study. Completion takes approximately 3-5 minutes. Thank you for supporting this study despite your busy schedule. | Survey text |

# S2. Eligibility questions and programmed response options

| **Code** | **中文原文** | **English translation** | **Analytic status** |
| --- | --- | --- | --- |
| S1 | 您是否年满18周岁？ | Are you 18 years of age or older? | Eligibility |
| S2 | 最近3个月，您是否从事过外卖、快递或同城配送工作？ | During the past three months, have you engaged in food delivery, express delivery, or local instant delivery work? | Eligibility |
| S3 | 您的配送工作是否主要通过平台或系统接单、派单或管理？ | Is your delivery work mainly accepted, dispatched, or managed through a platform or system? | Eligibility |

**S1 response options**

| **Option** | **中文原文** | **English translation** |
| --- | --- | --- |
| 1 | 是 | Yes |
| 2 | 否 | No |

**S2 response options**

| **Option** | **中文原文** | **English translation** |
| --- | --- | --- |
| 1 | 是，每周都会做 | Yes, every week |
| 2 | 是，但只是偶尔做 | Yes, but only occasionally |
| 3 | 没有做过 | No |

**S3 response options**

| **Option** | **中文原文** | **English translation** |
| --- | --- | --- |
| 1 | 是 | Yes |
| 2 | 否 | No |
| 3 | 不确定 | Not sure |

**Eligibility used in the analysis:** Respondents were retained if S1 = Yes, S2 = Yes every week or Yes but only occasionally, and S3 = Yes. In the 660 initially obtained responses, all respondents selected Yes for S1 and S3; 362 selected weekly delivery work and 298 selected occasional delivery work. The final 650-case dataset was produced after subsequent response-quality screening, not by excluding additional cases on these three eligibility questions.

# S3. Work-background questions

| **Code** | **中文原文** | **English translation** | **Analytic status** |
| --- | --- | --- | --- |
| W1 | 您目前主要做哪类配送？ | What type of delivery work do you mainly do at present? | Background |
| W2 | 您主要使用哪个平台或系统接单、派单或管理配送？ | Which platform or system do you mainly use to accept orders, receive dispatches, or manage deliveries? | Background |
| W3 | 您现在做配送的方式是？ | What is your current delivery-work arrangement? | Background |
| W4 | 您从事配送工作大约多久了？ | Approximately how long have you worked in delivery? | Background |
| W5 | 最近一个月，您每天通常跑单或派件多久？ | During the past month, how many hours per day did you usually spend making deliveries or delivering parcels? | Background |
| W6 | 最近一个月，您每月通过配送大约收入多少？ | During the past month, approximately how much monthly income did you earn from delivery work? | Background |

**W1 response options**

| **Option** | **中文原文** | **English translation** |
| --- | --- | --- |
| 1 | 外卖配送为主 | Mainly food delivery |
| 2 | 快递派件/取件为主 | Mainly express-parcel delivery/pickup |
| 3 | 同城跑腿/即时配送为主 | Mainly local errand or instant delivery |
| 4 | 几种都做 | Several types of delivery work |

**W2 response options**

| **Option** | **中文原文** | **English translation** |
| --- | --- | --- |
| 1 | 美团/美团众包 | Meituan / Meituan Crowdsourcing |
| 2 | 饿了么/蜂鸟众包 | Ele.me / Fengniao Crowdsourcing |
| 3 | 顺丰/顺丰同城 | SF Express / SF Intra-city |
| 4 | 京东/达达 | JD / Dada |
| 5 | 闪送/同城跑腿平台 | FlashEx / local errand-delivery platforms |
| 6 | 三通一达/菜鸟等快递系统 | STO/YTO/ZTO/Yunda/Cainiao-type express systems |
| 7 | 多个平台差不多 | Several platforms, with no single main platform |
| 8 | 其他 | Other |

**W3 response options**

| **Option** | **中文原文** | **English translation** |
| --- | --- | --- |
| 1 | 专职做配送 | Full-time delivery work |
| 2 | 兼职做配送 | Part-time delivery work |
| 3 | 偶尔跑单/临时做 | Occasional or temporary delivery work |
| 4 | 其他 | Other |

**W4 response options**

| **Option** | **中文原文** | **English translation** |
| --- | --- | --- |
| 1 | 不满6个月 | Less than 6 months |
| 2 | 6个月—1年 | 6 months to 1 year |
| 3 | 1—3年 | 1–3 years |
| 4 | 3—5年 | 3–5 years |
| 5 | 5年以上 | More than 5 years |

**W5 response options**

| **Option** | **中文原文** | **English translation** |
| --- | --- | --- |
| 1 | 4小时以内 | Less than 4 hours |
| 2 | 4—6小时 | 4–6 hours |
| 3 | 6—8小时 | 6–8 hours |
| 4 | 8—10小时 | 8–10 hours |
| 5 | 10小时以上 | More than 10 hours |

**W6 response options**

| **Option** | **中文原文** | **English translation** |
| --- | --- | --- |
| 1 | 3000元以下 | Below RMB 3,000 |
| 2 | 3000—4999元 | RMB 3,000–4,999 |
| 3 | 5000—6999元 | RMB 5,000–6,999 |
| 4 | 7000—8999元 | RMB 7,000–8,999 |
| 5 | 9000—11999元 | RMB 9,000–11,999 |
| 6 | 12000元及以上 | RMB 12,000 or above |
| 7 | 不方便透露 | Prefer not to say |

# S4. Response scale for all focal construct items

| **Point** | **中文界面** | **English rendering** | **Status** |
| --- | --- | --- | --- |
| 1 | 非常不同意 | Strongly disagree | Displayed response point |
| 2 | 2 | 2 | Displayed response point |
| 3 | 3 | 3 | Displayed response point |
| 4 | 4 | 4 | Displayed response point |
| 5 | 5 | 5 | Displayed response point |
| 6 | 6 | 6 | Displayed response point |
| 7 | 非常同意 | Strongly agree | Displayed response point |

**Scale-label clarification:** The Wenjuanxing interface verbally anchored only the two endpoints. Points 2–6 were displayed numerically. Accordingly, the full seven displayed response points were: 1 = Strongly disagree; 2; 3; 4; 5; 6; 7 = Strongly agree.

# S5. Temporal algorithmic control (TAC)

| **Code** | **中文原文** | **English translation** | **Analytic status** |
| --- | --- | --- | --- |
| TAC1 | 平台用时限和提醒，安排骑手的配送节奏。 | The platform uses time limits and reminders to set the pace of delivery work. | Used in final model |
| TAC2 | 配送时，我会按系统时间赶进度。 | While making deliveries, I speed up to keep pace with the system’s timing. | Used in final model |
| TAC3 | 配送时，我会因连续派单加快速度。 | While making deliveries, I speed up when orders are assigned continuously. | Used in final model |
| TAC4 | 配送时，我会跟着倒计时调整节奏。 | While making deliveries, I adjust my pace according to the countdown. | Used in final model |

# S6. Emotional exhaustion (EEX)

Source note. EEX1–EEX4 were contextually adapted from the work-related burnout subscale of the Copenhagen Burnout Inventory (Kristensen et al., 2005). The original subscale contains seven work-related fatigue/exhaustion items. The present study used a four-item platform-delivery version and a seven-point agreement scale; the scores should not be interpreted as the original CBI work-related burnout score.

| **Code** | **中文原文** | **English translation** | **Analytic status** |
| --- | --- | --- | --- |
| EEX1 | 平台节奏持续较紧时，骑手容易感到心累。 | When the platform maintains a tight work pace, delivery workers are likely to feel emotionally tired. | Used in final model |
| EEX2 | 按平台节奏跑单时，我会感到心累。 | When working at the platform’s pace, I feel emotionally tired. | Used in final model |
| EEX3 | 按平台节奏跑单时，我会感到没劲。 | When working at the platform’s pace, I feel drained. | Used in final model |
| EEX4 | 按平台节奏跑单时，我会感到心里疲惫。 | When working at the platform’s pace, I feel emotionally worn out. | Used in final model |

# S7. Response algorithmic control (RAC)

| **Code** | **中文原文** | **English translation** | **Analytic status** |
| --- | --- | --- | --- |
| RAC1 | 系统通过派单和消息，提醒骑手及时回应。 | The system uses order-assignment notifications and messages to prompt delivery workers to respond promptly. | Used in final model |
| RAC2 | 送单时，我会很快查看派单提醒。 | While making deliveries, I quickly check order-assignment notifications. | Used in final model |
| RAC3 | 送单时，我会尽快回复用户消息。 | While making deliveries, I reply to customer messages as soon as possible. | Used in final model |
| RAC4 | 送单时，我会马上处理系统提示。 | While making deliveries, I act on system prompts immediately. | Used in final model |

# S8. Emotional feigning (EFD)

| **Code** | **中文原文** | **English translation** | **Analytic status** |
| --- | --- | --- | --- |
| EFD1 | 平台工作中，骑手有时需要压住情绪继续配送。 | In platform work, delivery workers sometimes need to suppress their emotions and continue making deliveries. | Used in final model |
| EFD2 | 面对工作要求时，我会把不满压住。 | When facing work demands, I suppress my dissatisfaction. | Used in final model |
| EFD3 | 面对工作要求时，我会把真实情绪收住。 | When facing work demands, I hold back my true emotions. | Used in final model |
| EFD4 | 面对工作要求时，我会保持平静继续配送。 | When facing work demands, I remain calm and continue making deliveries. | Used in final model |

# S9. Attention-check item

| **Code** | **中文原文** | **English translation** | **Analytic status** |
| --- | --- | --- | --- |
| ATT1 | 请选择“6”。界面中的陈述为“请根据提示选择”。 | Please select “6.” The statement displayed in the matrix row was “Please choose as instructed.” | Quality check |

**Scoring:** A correct response was 6. All 660 initially obtained responses selected 6 on this item.

# S10. Trajectory algorithmic control (TRC)

| **Code** | **中文原文** | **English translation** | **Analytic status** |
| --- | --- | --- | --- |
| TRC1 | 系统通过定位和路线，记录骑手的配送过程。 | The system uses location and route information to record the delivery process. | Used in final model |
| TRC2 | 配送时，我感觉位置一直被记录。 | While making deliveries, I feel that my location is continuously recorded. | Used in final model |
| TRC3 | 配送时，我知道路线变化会被记录。 | While making deliveries, I know that changes in my route are recorded. | Used in final model |
| TRC4 | 配送时，我知道停留或绕路会被记录。 | While making deliveries, I know that stops or detours are recorded. | Used in final model |

# S11. Emotional reactance (ERR)

| **Code** | **中文原文** | **English translation** | **Analytic status** |
| --- | --- | --- | --- |
| ERR1 | 系统要求较紧时，骑手有时会心里抵触。 | When system requirements are tight, delivery workers sometimes feel internally resistant. | Not used in final model |
| ERR2 | 遇到系统要求时，我会因为催得紧而烦躁。 | When facing system requirements, I feel irritated when I am pushed too hard. | Not used in final model |
| ERR3 | 遇到系统要求时，我会因为不合现场而不服气。 | When system requirements do not fit conditions on the ground, I feel unconvinced and dissatisfied. | Not used in final model |
| ERR4 | 遇到系统要求时，我会因为安排不灵活而不太想配合。 | When facing system requirements, I am less willing to cooperate when the arrangements are inflexible. | Not used in final model |

# S12. Direct perceived algorithmic control items (PAC1–PAC4)

| **Code** | **中文原文** | **English translation** | **Analytic status** |
| --- | --- | --- | --- |
| PAC1 | 平台通过派单、时限和评价，管理骑手的配送过程。 | The platform manages delivery workers’ delivery process through order assignment, time limits, and evaluations. | Not used in final model |
| PAC2 | 配送时，我会感觉接单送单受系统安排。 | While making deliveries, I feel that order acceptance and delivery are arranged by the system. | Not used in final model |
| PAC3 | 配送时，我会感觉时间路线受系统影响。 | While making deliveries, I feel that timing and routes are influenced by the system. | Not used in final model |
| PAC4 | 配送时，我会感觉配送表现受平台评价影响。 | While making deliveries, I feel that my delivery performance is influenced by platform evaluations. | Not used in final model |

# S13. Punitive compliance behavior (PC)

| **Code** | **中文原文** | **English translation** | **Analytic status** |
| --- | --- | --- | --- |
| PC1 | 平台规则范围内，骑手有时只做规定动作。 | Within platform rules, delivery workers sometimes perform only the prescribed actions. | Used in final model |
| PC2 | 按平台要求执行时，我有时只做规定动作。 | When carrying out platform requirements, I sometimes perform only the prescribed actions. | Used in final model |
| PC3 | 按平台要求执行时，我有时只做到基本要求。 | When carrying out platform requirements, I sometimes do only what is basically required. | Used in final model |
| PC4 | 按平台要求执行时，我有时只把流程走完。 | When carrying out platform requirements, I sometimes simply complete the required procedure. | Used in final model |

# S14. Demographic questions

| **Code** | **中文原文** | **English translation** | **Analytic status** |
| --- | --- | --- | --- |
| D1 | 您的性别是？ | What is your gender? | Demographic |
| D2 | 您的年龄是？ | What is your age? | Demographic |
| D3 | 您的最高学历是？ | What is the highest level of education you have completed? | Demographic |
| D4 | 您目前的婚姻状况是？ | What is your current marital status? | Demographic |

**D1 response options**

| **Option** | **中文原文** | **English translation** |
| --- | --- | --- |
| 1 | 男 | Male |
| 2 | 女 | Female |
| 3 | 不方便透露 | Prefer not to say |

**D2 response options**

| **Option** | **中文原文** | **English translation** |
| --- | --- | --- |
| 1 | 18—25岁 | 18–25 years |
| 2 | 26—35岁 | 26–35 years |
| 3 | 36—45岁 | 36–45 years |
| 4 | 46—55岁 | 46–55 years |
| 5 | 56岁及以上 | 56 years or older |

**D3 response options**

| **Option** | **中文原文** | **English translation** |
| --- | --- | --- |
| 1 | 初中及以下 | Junior middle school or below |
| 2 | 高中/中专/职高 | Senior high school / technical secondary school / vocational high school |
| 3 | 大专 | Junior college |
| 4 | 本科 | Bachelor’s degree |
| 5 | 研究生及以上 | Postgraduate degree or above |

**D4 response options**

| **Option** | **中文原文** | **English translation** |
| --- | --- | --- |
| 1 | 未婚 | Unmarried |
| 2 | 已婚 | Married |
| 3 | 离异或丧偶 | Divorced or widowed |
| 4 | 不方便透露 | Prefer not to say |

# S15. Optional survey code

| **Code** | **中文原文** | **English translation** | **Analytic status** |
| --- | --- | --- | --- |
| CODE | 调研编码（选择填写，如果没有，可不填写。） | Survey code (optional; leave blank if none was provided). | Administrative field |

**End of Supplementary Appendix S1**
