# Supplementary material for: Dimensions of perceived algorithmic control and endorsement of minimum-only execution in platform delivery work
Source: Front Psychol. 2026 Oct 7;17:1937810. doi: 10.3389/fpsyg.2026.1937810 (PMC13683074; doi:10.3389/fpsyg.2026.1937810)
Supplement: Supplementary file 1 [file Data_Sheet_1.ZIP › Supplementary Appendix S2 Item Evidence and Robustness.docx]

Supplementary Appendix S2. Item-Level Provenance, Descriptive Statistics, Cross-Loadings, and Robustness Analyses

*Item evidence and historical sensitivity analyses | Final analysis sample: N = 650*

This appendix presents the administered items and their sources, checked descriptive statistics, archived measurement results, and historical sensitivity reports. The EEX measure was informed by the Copenhagen Burnout Inventory work-related burnout subscale, not the Maslach Burnout Inventory. S2.1 identifies the provenance and verification scope; Appendix S12 reports the revised native dimension-level analysis.

# S2.1 Analytical provenance

The final item-level dataset contained 650 cases and 24 analyzed items. Its item records, including repeated records, match the shared analytical dataset; the two files use different row orders. The original SmartPLS values were checked against the archived final results workbook. These checks establish the correspondence of the identified data and reported output; they do not recover the historical SmartPLS project or all intermediate analyses.

The earlier version described the loading and cross-loading diagnostics as a reflective Mode-A PLS-PM reconstruction and reported a maximum absolute difference of 0.012 from the archived SmartPLS own-loadings. It described the item-exclusion analyses as reflective disjoint two-stage models with 10,000 bootstrap resamples and two-tailed percentile 95% confidence intervals. The corresponding original code, complete estimation output, and resample files have not been recovered. The reconstruction columns and Tables S4–S6 therefore preserve the historical report; they are not presented as results independently reproduced during the present revision.

The source-content descriptions combine published measurement domains, administered wording, and the retained item-development record. For TRC, the original Pei et al. (2021) scale was checked directly, and the workbook documents delivery-context rewriting and successive shortened versions; the final version matches the administered questionnaire. “Informed by” identifies a conceptual reference rather than unchanged administration. The documented adaptations do not establish metric equivalence with the source instruments.

The item-development workbook was created on 12 April 2026 and last modified on 16 April 2026, before the survey opened on 18 May 2026. For each construct, it recorded the operational definition, source scale and citation, rationale for source selection, an English summary of source-item content, a Chinese semantic rendering, a platform-delivery contextualized version, a shortened candidate version, and the source location. The documented procedure was author-led source review, semantic rendering, and contextual rewriting; the records do not establish a formal translation-back-translation protocol.

# S2.2 Item-level provenance and contextualization map

Supplementary Table S2. Construct panels report the English rendering of the administered items, their source references, and the content represented by the present wording. Appendix S1 preserves the administered Chinese questionnaire. For TRC, the map combines the original tracking-and-evaluation items in Pei et al. (2021, Table 3, p. 19) with the documented delivery-context revisions.

## Table S2, Panel A: TAC

| **Item** | **English reporting rendering of administered wording** | **Source anchor** | **Source content/function summary** | **Present wording and content represented** | **Boundary / interpretive caution** |
| --- | --- | --- | --- | --- | --- |
| TAC1 | The platform uses time limits and reminders to set the pace of delivery work. | Conceptual reference: Quantitative Workload Inventory (QWI) | speed/time-scarcity content in QWI | The item names platform time limits and reminders as sources of delivery pace pressure. | The wording contains both a platform control cue and workload-related pace content; it is not a pure measure of an external algorithmic feature. |
| TAC2 | While making deliveries, I speed up to keep pace with the system’s timing. | Conceptual reference: Quantitative Workload Inventory (QWI) | work-fast/time-scarcity content in QWI | The item combines system timing with the worker’s reported acceleration. | Substantial work-pace and response-behavior content; retained as administered and examined in the historical exclusion analyses. |
| TAC3 | While making deliveries, I speed up when orders are assigned continuously. | Conceptual reference: Quantitative Workload Inventory (QWI) | high-quantity/work-fast content in QWI | The item combines continuous order assignment with the worker’s reported acceleration. | Substantial dispatch, workload, and response-behavior content; it does not isolate general workload from algorithmic control. |
| TAC4 | While making deliveries, I adjust my pace according to the countdown. | Conceptual reference: Quantitative Workload Inventory (QWI) | no close one-to-one QWI item; derived from time-pressure theme | The item names a countdown and the worker’s adjustment of pace. | The countdown is a specific platform cue, but the response still combines exposure with personal adjustment. |

## Table S2, Panel B: RAC

| **Item** | **English reporting rendering of administered wording** | **Source anchor** | **Source content/function summary** | **Present wording and content represented** | **Boundary / interpretive caution** |
| --- | --- | --- | --- | --- | --- |
| RAC1 | The system uses order-assignment notifications and messages to prompt delivery workers to respond promptly. | Conceptual reference: Workplace Telepressure Measure | telepressure concern/urge to respond quickly | The item names system notifications and messages that prompt a timely response. | The external prompt differs from telepressure as an internal preoccupation or urge; the present measure is conceptually informed by that literature. |
| RAC2 | While making deliveries, I quickly check order-assignment notifications. | Conceptual reference: Workplace Telepressure Measure | telepressure concern about response speed | The item concerns quickly checking order-assignment notifications. | It reports response behavior and does not separately measure the strength of an externally imposed requirement. |
| RAC3 | While making deliveries, I reply to customer messages as soon as possible. | Conceptual reference: Workplace Telepressure Measure | telepressure immediate response to work messages | The item concerns promptly replying to customer messages during delivery. | Customer messages are not exclusively algorithmic; the item supports a contextual response-demand interpretation. |
| RAC4 | While making deliveries, I act on system prompts immediately. | Conceptual reference: Workplace Telepressure Measure | telepressure immediate response urge | The item combines system prompts with the worker’s immediate action. | It combines an external cue with response behavior and is not a pure measure of system requirements. |

## Table S2, Panel C: TRC

| **Item** | **English reporting rendering of administered wording** | **Source anchor** | **Source content/function summary** | **Present wording and content represented** | **Boundary / interpretive caution** |
| --- | --- | --- | --- | --- | --- |
| TRC1 | The system uses location and route information to record the delivery process. | Adapted from the tracking-and-evaluation dimension in Pei et al. (2021, Table 3, p. 19), with delivery-context revisions documented in the item-development workbook. | The source dimension includes location and work-progress tracking, work-attitude monitoring, and completion-quality evaluation. | The administered item combines location and route information with recording of the delivery process. | Combines location and delivery-process recording with route information added during contextual revision. |
| TRC2 | While making deliveries, I feel that my location is continuously recorded. | Adapted from the tracking-and-evaluation dimension in Pei et al. (2021, Table 3, p. 19), with delivery-context revisions documented in the item-development workbook. | The original location item concerns real-time tracking of the worker’s geographical location. | The administered item describes the worker's perception that location is continuously recorded. | Focuses on location-recording awareness in delivery work. |
| TRC3 | While making deliveries, I know that changes in my route are recorded. | Adapted from the tracking-and-evaluation dimension in Pei et al. (2021, Table 3, p. 19), with delivery-context revisions documented in the item-development workbook. | The original dimension includes location and work-progress tracking; route-change wording emerged during the delivery-context revisions. | The administered item names awareness that changes in the delivery route are recorded. | Specifies route-recording awareness; the final item does not directly retain work-attitude monitoring. |
| TRC4 | While making deliveries, I know that stops or detours are recorded. | Adapted from the tracking-and-evaluation dimension in Pei et al. (2021, Table 3, p. 19), with delivery-context revisions documented in the item-development workbook. | The original dimension includes location and work-progress tracking; stops and detours were specified during the delivery-context revisions. | The administered item names awareness that stops or detours are recorded. | Specifies awareness that stops or detours are recorded; the final block does not directly retain completion-quality evaluation. |

## Table S2, Panel D: EEX

| **Item** | **English reporting rendering of administered wording** | **Source anchor** | **Source content/function summary** | **Present wording and content represented** | **Boundary / interpretive caution** |
| --- | --- | --- | --- | --- | --- |
| EEX1 | When the platform maintains a tight work pace, delivery workers are likely to feel emotionally tired. | Copenhagen Burnout Inventory—Work-related Burnout subscale | work-related emotional exhaustion and feeling exhausted because of work | The item combines platform pace with third-person wording about emotional tiredness. | Substantial contextual and response-format adaptation; this is not the original seven-item CBI work-related burnout scale and is not interpreted with the CBI 0–100 scoring procedure. |
| EEX2 | When working at the platform’s pace, I feel emotionally tired. | Copenhagen Burnout Inventory—Work-related Burnout subscale | fatigue/exhaustion attributed to work and feeling worn out by the working day | The item describes first-person emotional tiredness at the platform’s pace. | Substantial contextual and response-format adaptation; this is not the original seven-item CBI work-related burnout scale and is not interpreted with the CBI 0–100 scoring procedure. |
| EEX3 | When working at the platform’s pace, I feel drained. | Copenhagen Burnout Inventory—Work-related Burnout subscale | feeling worn out or burned out because of work | The item describes feeling drained while working at the platform’s pace. | Substantial contextual and response-format adaptation; this is not the original seven-item CBI work-related burnout scale and is not interpreted with the CBI 0–100 scoring procedure. |
| EEX4 | When working at the platform’s pace, I feel emotionally worn out. | Copenhagen Burnout Inventory—Work-related Burnout subscale | emotional exhaustion and fatigue attributed to work | The item describes feeling emotionally worn out while working at the platform’s pace. | Substantial contextual and response-format adaptation; this is not the original seven-item CBI work-related burnout scale and is not interpreted with the CBI 0–100 scoring procedure. |

## Table S2, Panel E: EFD

| **Item** | **English reporting rendering of administered wording** | **Source anchor** | **Source content/function summary** | **Present wording and content represented** | **Boundary / interpretive caution** |
| --- | --- | --- | --- | --- | --- |
| EFD1 | In platform work, delivery workers sometimes need to suppress their emotions and continue making deliveries. | Emotional Dissonance Scale / FEWS | display/suppression inconsistent with true feelings | The item describes suppressing emotions while continuing delivery work, using third-person wording. | Measures display regulation, not reduced effort. |
| EFD2 | When facing work demands, I suppress my dissatisfaction. | Emotional Dissonance Scale / FEWS | suppression of true feelings | The item describes suppressing dissatisfaction under work demands. | Low conceptual risk. |
| EFD3 | When facing work demands, I hold back my true emotions. | Emotional Dissonance Scale / FEWS | suppression of genuine emotion | The item describes holding back genuine emotions under work demands. | Low conceptual risk. |
| EFD4 | When facing work demands, I remain calm and continue making deliveries. | Emotional Dissonance Scale / FEWS | neutral outward display despite inner emotion | The item describes outward calm and continued delivery work. | Outward calm and continued work do not by themselves establish an inner–outer emotional discrepancy. Historical cross-loadings are retained subject to the source-verification limitation in S2.1. |

## Table S2, Panel F: PC

The abbreviation PC and the context-specific label punitive compliance were retained for continuity with the original analysis.

| **Item** | **English reporting rendering of administered wording** | **Source anchor** | **Source content/function summary** | **Present wording and content represented** | **Boundary / interpretive caution** |
| --- | --- | --- | --- | --- | --- |
| PC1 | Within platform rules, delivery workers sometimes perform only the prescribed actions. | Surface Compliance Scale | minimum-effort/procedure-following content in surface compliance | The item describes performing only prescribed actions within platform rules, using third-person wording. | Does not measure punitive intent; supports a narrower ‘reduced discretionary effort within compliance’ interpretation. |
| PC2 | When carrying out platform requirements, I sometimes perform only the prescribed actions. | Surface Compliance Scale | minimum-effort/procedure-following content in surface compliance | The item describes first-person performance of only prescribed actions under platform requirements. | The content overlaps PC1. Internal measurement statistics do not establish distinctiveness from adjacent constructs not measured in this survey. |
| PC3 | When carrying out platform requirements, I sometimes do only what is basically required. | Surface Compliance Scale | ticking off tasks/minimum cognitive investment | The item describes doing only what is basically required. | Does not distinguish punitive motive from other reasons for minimum effort. |
| PC4 | When carrying out platform requirements, I sometimes simply complete the required procedure. | Surface Compliance Scale | completing procedural steps without deeper engagement | The item describes procedural completion without specifying additional discretionary effort. | Supports rule-bounded minimum execution, not retaliation. |

# S2.3 Item-level descriptive statistics and loading diagnostics

Supplementary Table S3. Means, sample standard deviations, sample variances, minima, and maxima were checked from the final N = 650 item data. Archived loadings are reported from the original SmartPLS workbook. Reconstructed loadings and cross-loadings are retained from the earlier appendix. The RAC2 standard deviation is 1.053 and the EFD2 sample variance is 1.279 after correction of the last reported decimal.

| **Construct** | **Item** | **Mean** | **SD** | **Variance** | **Min** | **Max** | **Locked loading** | **Reconstructed loading** | **Largest cross-loading** | **Own - largest cross** |
| --- | --- | --- | --- | --- | --- | --- | --- | --- | --- | --- |
| TAC | TAC1 | 5.051 | 1.070 | 1.145 | 1 | 7 | 0.785 | 0.784 | 0.394 | 0.390 |
| TAC | TAC2 | 4.635 | 1.081 | 1.169 | 1 | 7 | 0.800 | 0.795 | 0.411 | 0.384 |
| TAC | TAC3 | 5.089 | 1.084 | 1.175 | 1 | 7 | 0.803 | 0.811 | 0.420 | 0.391 |
| TAC | TAC4 | 4.843 | 1.078 | 1.162 | 1 | 7 | 0.790 | 0.779 | 0.412 | 0.367 |
| RAC | RAC1 | 5.340 | 1.086 | 1.180 | 1 | 7 | 0.805 | 0.805 | 0.432 | 0.374 |
| RAC | RAC2 | 5.480 | 1.053 | 1.110 | 2 | 7 | 0.776 | 0.784 | 0.435 | 0.349 |
| RAC | RAC3 | 4.998 | 1.198 | 1.435 | 1 | 7 | 0.806 | 0.798 | 0.451 | 0.347 |
| RAC | RAC4 | 5.098 | 1.147 | 1.315 | 1 | 7 | 0.748 | 0.745 | 0.410 | 0.335 |
| TRC | TRC1 | 5.148 | 1.092 | 1.192 | 1 | 7 | 0.817 | 0.814 | 0.430 | 0.385 |
| TRC | TRC2 | 5.069 | 1.156 | 1.337 | 1 | 7 | 0.811 | 0.815 | 0.465 | 0.350 |
| TRC | TRC3 | 5.145 | 1.102 | 1.215 | 1 | 7 | 0.817 | 0.818 | 0.424 | 0.394 |
| TRC | TRC4 | 4.752 | 1.136 | 1.290 | 1 | 7 | 0.788 | 0.790 | 0.464 | 0.326 |
| EEX | EEX1 | 4.857 | 1.053 | 1.109 | 2 | 7 | 0.799 | 0.793 | 0.392 | 0.401 |
| EEX | EEX2 | 5.092 | 1.084 | 1.175 | 1 | 7 | 0.749 | 0.744 | 0.335 | 0.409 |
| EEX | EEX3 | 5.189 | 1.088 | 1.183 | 1 | 7 | 0.778 | 0.788 | 0.391 | 0.398 |
| EEX | EEX4 | 4.832 | 1.101 | 1.212 | 1 | 7 | 0.776 | 0.776 | 0.391 | 0.385 |
| EFD | EFD1 | 5.371 | 1.192 | 1.420 | 1 | 7 | 0.803 | 0.801 | 0.331 | 0.470 |
| EFD | EFD2 | 5.443 | 1.131 | 1.279 | 1 | 7 | 0.822 | 0.825 | 0.372 | 0.453 |
| EFD | EFD3 | 5.286 | 1.175 | 1.382 | 1 | 7 | 0.811 | 0.809 | 0.362 | 0.447 |
| EFD | EFD4 | 4.929 | 1.232 | 1.517 | 1 | 7 | 0.839 | 0.838 | 0.376 | 0.462 |
| PC | PC1 | 6.026 | 0.919 | 0.845 | 2 | 7 | 0.757 | 0.755 | 0.374 | 0.381 |
| PC | PC2 | 5.968 | 0.981 | 0.962 | 2 | 7 | 0.752 | 0.745 | 0.385 | 0.360 |
| PC | PC3 | 5.680 | 1.003 | 1.007 | 2 | 7 | 0.800 | 0.788 | 0.404 | 0.385 |
| PC | PC4 | 5.991 | 0.985 | 0.971 | 1 | 7 | 0.759 | 0.763 | 0.404 | 0.359 |

# S2.4 Full item-level loading and cross-loading matrix

Supplementary Table S4. Historical reported correlations between each standardized item and the reconstructed first-order construct scores. The intended loading is bolded. Each row reports a larger intended loading than its cross-loadings.

| **Construct** | **Item** | **TAC** | **RAC** | **TRC** | **EEX** | **EFD** | **PC** |
| --- | --- | --- | --- | --- | --- | --- | --- |
| TAC | TAC1 | 0.784 | 0.344 | 0.394 | 0.247 | 0.331 | 0.391 |
| TAC | TAC2 | 0.795 | 0.367 | 0.377 | 0.244 | 0.328 | 0.411 |
| TAC | TAC3 | 0.811 | 0.378 | 0.399 | 0.270 | 0.373 | 0.420 |
| TAC | TAC4 | 0.779 | 0.375 | 0.412 | 0.228 | 0.318 | 0.406 |
| RAC | RAC1 | 0.373 | 0.805 | 0.432 | 0.286 | 0.311 | 0.364 |
| RAC | RAC2 | 0.362 | 0.784 | 0.435 | 0.256 | 0.295 | 0.380 |
| RAC | RAC3 | 0.384 | 0.798 | 0.451 | 0.253 | 0.335 | 0.338 |
| RAC | RAC4 | 0.327 | 0.745 | 0.410 | 0.245 | 0.301 | 0.329 |
| TRC | TRC1 | 0.413 | 0.430 | 0.814 | 0.381 | 0.347 | 0.240 |
| TRC | TRC2 | 0.433 | 0.465 | 0.815 | 0.400 | 0.365 | 0.283 |
| TRC | TRC3 | 0.391 | 0.424 | 0.818 | 0.401 | 0.332 | 0.236 |
| TRC | TRC4 | 0.375 | 0.464 | 0.790 | 0.397 | 0.338 | 0.257 |
| EEX | EEX1 | 0.300 | 0.283 | 0.392 | 0.793 | 0.225 | 0.314 |
| EEX | EEX2 | 0.182 | 0.215 | 0.335 | 0.744 | 0.163 | 0.245 |
| EEX | EEX3 | 0.276 | 0.265 | 0.391 | 0.788 | 0.207 | 0.330 |
| EEX | EEX4 | 0.197 | 0.261 | 0.391 | 0.776 | 0.191 | 0.280 |
| EFD | EFD1 | 0.331 | 0.281 | 0.309 | 0.182 | 0.801 | 0.327 |
| EFD | EFD2 | 0.353 | 0.349 | 0.354 | 0.219 | 0.825 | 0.372 |
| EFD | EFD3 | 0.362 | 0.320 | 0.357 | 0.242 | 0.809 | 0.332 |
| EFD | EFD4 | 0.350 | 0.344 | 0.376 | 0.190 | 0.838 | 0.312 |
| PC | PC1 | 0.374 | 0.335 | 0.224 | 0.315 | 0.315 | 0.755 |
| PC | PC2 | 0.385 | 0.347 | 0.222 | 0.253 | 0.337 | 0.745 |
| PC | PC3 | 0.404 | 0.355 | 0.291 | 0.333 | 0.300 | 0.788 |
| PC | PC4 | 0.404 | 0.338 | 0.221 | 0.257 | 0.303 | 0.763 |

# S2.5 Sensitivity analyses for workload-like temporal-control items

TAC2 and TAC3 were identified as the items closest to generic work-pace or workload content. TAC1 and TAC4 name platform time limits/reminders and countdowns more directly. Single-item exclusions retained three-item TAC blocks; the joint exclusion is a stringent two-item sensitivity check and is not proposed as a replacement measurement model.

## Table S5, Panel A: Point estimates across exclusion specifications

| **Metric** | **Locked SmartPLS baseline** | **Reconstructed baseline** | **Drop TAC2** | **Drop TAC3** | **Drop TAC2 + TAC3** | **Max absolute change** |
| --- | --- | --- | --- | --- | --- | --- |
| PAC -> EEX | 0.462 | 0.462 | 0.461 | 0.458 | 0.456 | 0.006 |
| PAC -> EFD | 0.508 | 0.498 | 0.495 | 0.486 | 0.479 | 0.019 |
| EEX -> EFD | 0.015 | 0.025 | 0.027 | 0.033 | 0.037 | 0.012 |
| PAC -> PC | 0.347 | 0.346 | 0.336 | 0.335 | 0.318 | 0.027 |
| EEX -> PC | 0.167 | 0.173 | 0.176 | 0.177 | 0.184 | 0.011 |
| EFD -> PC | 0.193 | 0.190 | 0.195 | 0.197 | 0.206 | 0.016 |
| PAC -> EEX -> PC | 0.077 | 0.080 | 0.081 | 0.081 | 0.084 | 0.004 |
| PAC -> EFD -> PC | 0.098 | 0.095 | 0.097 | 0.096 | 0.099 | 0.004 |
| PAC -> EEX -> EFD -> PC | 0.001 | 0.002 | 0.002 | 0.003 | 0.004 | 0.001 |
| R2 EEX | 0.213 | 0.213 | 0.212 | 0.210 | 0.208 | 0.006 |
| R2 EFD | 0.265 | 0.261 | 0.259 | 0.252 | 0.247 | 0.014 |
| R2 PC | 0.324 | 0.325 | 0.321 | 0.321 | 0.315 | 0.010 |

## Table S5, Panel B: Historical 10,000-resample bootstrap report for the joint TAC2/TAC3 exclusion

*Note. R-squared rows are descriptive; bootstrap p values are not reported for R-squared.*

| **Metric** | **Estimate** | **Bootstrap mean** | **Bootstrap SD** | **2.5%** | **97.5%** | **Two-tailed p** |
| --- | --- | --- | --- | --- | --- | --- |
| PAC -> EEX | 0.456 | 0.458 | 0.032 | 0.395 | 0.519 | <.001 |
| PAC -> EFD | 0.479 | 0.480 | 0.034 | 0.413 | 0.544 | <.001 |
| EEX -> EFD | 0.037 | 0.037 | 0.037 | -0.036 | 0.109 | 0.309 |
| PAC -> PC | 0.318 | 0.320 | 0.039 | 0.242 | 0.395 | <.001 |
| EEX -> PC | 0.184 | 0.184 | 0.036 | 0.113 | 0.254 | <.001 |
| EFD -> PC | 0.206 | 0.206 | 0.038 | 0.131 | 0.280 | <.001 |
| PAC -> EEX -> PC | 0.084 | 0.084 | 0.018 | 0.050 | 0.120 | <.001 |
| PAC -> EFD -> PC | 0.099 | 0.099 | 0.019 | 0.062 | 0.138 | <.001 |
| PAC -> EEX -> EFD -> PC | 0.004 | 0.004 | 0.004 | -0.003 | 0.011 | 0.309 |
| R2 EEX | 0.208 | 0.211 | 0.029 | 0.156 | 0.269 |  |
| R2 EFD | 0.247 | 0.250 | 0.028 | 0.196 | 0.305 |  |
| R2 PC | 0.315 | 0.320 | 0.028 | 0.265 | 0.376 |  |

## Table S6. Historical TAC measurement-quality report across exclusion specifications

| **Model** | **TAC items** | **k** | **Cronbach alpha** | **Composite reliability** | **AVE** | **Minimum loading** | **Maximum loading** |
| --- | --- | --- | --- | --- | --- | --- | --- |
| Baseline_TAC1-4 | TAC1, TAC2, TAC3, TAC4 | 4 | 0.802 | 0.871 | 0.628 | 0.779 | 0.811 |
| Drop_TAC3 | TAC1, TAC2, TAC4 | 3 | 0.746 | 0.855 | 0.663 | 0.809 | 0.823 |
| Drop_TAC2_TAC3 | TAC1, TAC4 | 2 | 0.650 | 0.851 | 0.741 | 0.859 | 0.862 |
| Drop_TAC2 | TAC1, TAC3, TAC4 | 3 | 0.750 | 0.857 | 0.666 | 0.801 | 0.837 |

# S2.6 Interpretive summary and limits

- Across the 24 analyzed items, means ranged from 4.635 to 6.026 and sample variances from 0.845 to 1.517.
- The historical loading table reports a largest cross-loading of 0.465 and a smallest own-loading advantage of 0.326.
- The retained exclusion tables report positive principal direct associations and positive two-coefficient products involving EEX alone and EFD alone across the specified exclusions.
- In the retained joint-exclusion report, the EEX–EFD coefficient and the three-coefficient PAC–EEX–EFD–PC product were nonsignificant. This coefficient pattern does not establish a parallel temporal process.
- The historical tables report PC R² of 0.324 in the original SmartPLS model and 0.315 in the joint-exclusion reconstruction. These values belong to different reported specifications and do not replace the revised native dimension-level result.
- The joint exclusion leaves two TAC items and a reported alpha of 0.650; it remains a sensitivity check rather than a replacement measure. Direct calculations from the identified item data corroborated the TAC alpha values for the four item sets. The current standardized alphas for TAC, RAC, TRC, and PC differ from the archived values by .001–.006, with all four remaining above .70. Table S11.1a retains the historical and verified values; the source of these small differences remains unresolved.
- TAC and RAC combine system cues with pace adjustment, checking, replying, or immediate action. The documented TRC adaptation from Pei et al. (2021) focuses on location, route changes, stops, and detours; the final items do not directly retain the source work-attitude and completion-quality content. EEX is a four-item, seven-point contextual measure informed by the seven-item CBI work-related burnout subscale, not the original CBI score or its 0–100 scoring system. PC denotes rule-bounded reduced discretionary effort and does not measure retaliatory intent. No independent pre-fieldwork back-translation record has been established.

# S2.7 References cited in the adaptation map

Spector, P. E., & Jex, S. M. (1998). Development of four self-report measures of job stressors and strain: Interpersonal Conflict at Work Scale, Organizational Constraints Scale, Quantitative Workload Inventory, and Physical Symptoms Inventory. Journal of Occupational Health Psychology, 3(4), 356–367. https://doi.org/10.1037/1076-8998.3.4.356

Official QWI description and response format (Paul Spector). https://paulspector.com/assessments/pauls-no-cost-assessments/quantitative-workload-inventory-qwi/

Barber, L. K., & Santuzzi, A. M. (2015). Please respond ASAP: Workplace telepressure and employee recovery. Journal of Occupational Health Psychology, 20(2), 172–189. https://doi.org/10.1037/a0038278

Pei, J. L., Liu, S. S., Cui, X., & Qu, J. J. (2021). Perceived algorithmic control of gig workers: Conceptualization, measurement and verification the impact on service performance. Nankai Business Review, 24(6), 14–25.

Zhu, J., Zhang, B., & Wang, H. (2024). The double-edged sword effects of perceived algorithmic control on platform workers' service performance. Humanities and Social Sciences Communications, 11, 316. https://doi.org/10.1057/s41599-024-02812-0

Kristensen, T. S., Borritz, M., Villadsen, E., & Christensen, K. B. (2005). The Copenhagen Burnout Inventory: A new tool for the assessment of burnout. Work & Stress, 19(3), 192–207. https://doi.org/10.1080/02678370500297720

Zapf, D., Vogt, C., Seifert, C., Mertini, H., & Isic, A. (1999). Emotion work as a source of stress: The concept and development of an instrument. European Journal of Work and Organizational Psychology, 8(3), 371–400. https://doi.org/10.1080/135943299398230

Hu, X., Yeo, G., & Griffin, M. A. (2020). More to safety compliance than meets the eye: Differentiating deep compliance from surface compliance. Safety Science, 130, 104852. https://doi.org/10.1016/j.ssci.2020.104852
