# Supplementary material for: Dimensions of perceived algorithmic control and endorsement of minimum-only execution in platform delivery work
Source: Front Psychol. 2026 Oct 7;17:1937810. doi: 10.3389/fpsyg.2026.1937810 (PMC13683074; doi:10.3389/fpsyg.2026.1937810)
Supplement: Supplementary file 1 [file Data_Sheet_1.ZIP › Supplementary Appendix S3 Construct Boundary.docx]

Supplementary Appendix S3

**Item-level construct-boundary analysis for the focal outcome measure**

Purpose. This appendix responds to the request to map each of the four focal outcome items onto published definitions of adjacent constructs. The exercise is interpretive and item-based. It does not substitute for a same-sample discriminant-validity test because the adjacent constructs were not measured in the survey.

Terminology. The revised manuscript uses minimum-only execution to denote self-reported endorsement of statements about performing only prescribed or basic actions within platform requirements. PC is retained as the historical outcome code. PC1 asks about delivery workers in general, while PC2–PC4 concern the respondent's own conduct. The items do not directly assess retaliatory or punitive intent, and the study does not claim to have established a new construct distinct from work-to-rule or reduced organizational citizenship behavior.

Quiet quitting provides an additional close comparison. Patel et al. (2025) combine a behavioral component of minimal work contribution with an emotional component evaluating that contribution. The PC items address related behavioral content but omit those contribution-related evaluations. EEX and EFD do not supply the missing component: they concern exhaustion and emotion regulation. The present study therefore neither equates PC with that quiet-quitting measure nor establishes empirical distinctiveness from it.

## Table S3A. Adjacent constructs and the boundary supported by the present items

| **Adjacent construct** | **Published definitional focus** | **Relationship to the four PC items** | **What the present study can and cannot claim** |
| --- | --- | --- | --- |
| Surface compliance | Outward compliance without full internalization of the rule's purpose (Hu et al., 2020). | The PC items mention platform requirements and prescribed procedures, but they do not ask whether workers accept or internalize the purpose of those requirements. | Content focus differs, but empirical distinctiveness is not established because rule internalization was not measured. |
| Work-to-rule | Strict adherence to formal rules while withholding activity beyond what rules or job requirements specify; it may be used as a resistance tactic (Hale and Borys, 2013; Alcadipani et al., 2018). | This is the closest adjacent construct. All four PC items combine a formal-requirement frame with minimum-only execution. | The current items do not assess collective action, deliberate slowdown, or coordinated protest. Their absence does not establish a distinct construct; the score represents endorsement of minimum-only execution statements in a platform-work context. |
| Reduced organizational citizenship behavior | A reduction in discretionary, extra-role contributions that are not formally required (Organ, 1997). | PC3 most directly overlaps with reduced discretionary contribution; the other items also restrict action to prescribed or basic requirements. | The survey did not measure helping, civic virtue, sportsmanship, or other OCB domains. The measure therefore cannot be shown to be distinct from reduced citizenship behavior. |
| Organizational withdrawal | Behavior used to avoid or distance oneself from dissatisfying work, including lateness, absence, turnover intentions, and related withdrawal repertoires (Hanisch and Hulin, 1990). | The PC items do not ask about absence, lateness, leaving, or temporal/physical avoidance of work. | The item content is narrower than organizational withdrawal, but no same-sample comparison was conducted. |
| Workplace deviance / counterproductive work behavior | Voluntary conduct that violates significant organizational norms or threatens the organization or its members (Robinson and Bennett, 1995; Bennett and Robinson, 2000). | The PC items are explicitly framed within platform rules and do not ask about norm violation, sabotage, or intentional harm. | At the item-content level, the measure does not operationalize deviance. This does not establish its uniqueness relative to all non-deviant low-effort behaviors. |
| Passive resistance / everyday resistance | Low-visibility or indirect opposition that may avoid overt confrontation (Scott, 1985; Alcadipani et al., 2018). | Minimum-only execution could function as passive resistance when motivated by opposition, but the present items do not measure that motive. | The study cannot infer oppositional or retaliatory intent from the PC scores. |

## Table S3B. Item-level mapping

| **Item** | **Administered item (English reporting translation)** | **Formal-requirement cue** | **Minimum-only cue** | **Closest overlap** | **Boundary indicated by the wording** |
| --- | --- | --- | --- | --- | --- |
| PC1 | Within platform rules, delivery workers sometimes perform only the prescribed actions. | "Within platform rules" | "only the prescribed actions" | Work-to-rule; reduced discretionary effort | Expresses a judgment about delivery workers in general, rather than a direct report of the respondent's own conduct. It combines a rule frame with minimum-only execution but does not measure motive, collective action, or a specific discretionary contribution. |
| PC2 | When carrying out platform requirements, I sometimes perform only the prescribed actions. | "carrying out platform requirements" | "only the prescribed actions" | Work-to-rule; surface compliance | Indicates procedural completion at the prescribed level; it does not assess rule internalization or retaliatory intent. |
| PC3 | When carrying out platform requirements, I sometimes do only what is basically required. | "carrying out platform requirements" | "only what is basically required" | Reduced organizational citizenship behavior; work-to-rule | Refers to doing only what is basically required. It does not independently establish basic compliance or quantify effort beyond that requirement. |
| PC4 | When carrying out platform requirements, I sometimes simply complete the required procedure. | "carrying out platform requirements" | "simply complete the required procedure" | Surface compliance; work-to-rule | Captures procedural completion without extra involvement; it does not determine whether the respondent accepts the rule's purpose or intends to obstruct the platform. |

## Interpretive conclusion

Taken together, the four items provide a measure of endorsement of minimum-only execution statements within a formal platform-requirement frame. The score combines one judgment about delivery workers generally with three personal reports, so it should not be described as four direct reports of individual behavior. The items do not measure retaliatory motive or independently validated compliance and discretionary-effort components, and they do not establish discriminant validity against work-to-rule, citizenship behavior, or quiet quitting. Further construct-development work should assess basic compliance, discretionary contribution, and relevant adjacent measures in the same sample; a punitive motive should be measured directly if it is part of the research question.

A further scoring boundary follows from the item wording. Higher scores indicate stronger endorsement of the minimum-only statements. Lower scores cannot be interpreted unambiguously as greater discretionary cooperation or failure to meet basic requirements. The scale therefore does not identify effort differences among workers independently shown to be equally compliant, nor does it define a bipolar continuum from full cooperation to noncompliance.

## References

Alcadipani, R., Hassard, J., and Islam, G. (2018). "I shot the sheriff": Irony, sarcasm and the changing nature of workplace resistance. Journal of Management Studies, 55(8), 1452-1487. https://doi.org/10.1111/joms.12356

Bennett, R. J., and Robinson, S. L. (2000). Development of a measure of workplace deviance. Journal of Applied Psychology, 85(3), 349-360. https://doi.org/10.1037/0021-9010.85.3.349

Hale, A., and Borys, D. (2013). Working to rule, or working safely? Part 1: A state of the art review. Safety Science, 55, 207-221. https://doi.org/10.1016/j.ssci.2012.05.011

Hanisch, K. A., and Hulin, C. L. (1990). Job attitudes and organizational withdrawal: An examination of retirement and other voluntary withdrawal behaviors. Journal of Vocational Behavior, 37(1), 60-78. https://doi.org/10.1016/0001-8791(90)90007-O

Hu, X., Yeo, G., and Griffin, M. A. (2020). More to safety compliance than meets the eye: Differentiating deep compliance from surface compliance. Safety Science, 130, 104852. https://doi.org/10.1016/j.ssci.2020.104852

Organ, D. W. (1997). Organizational citizenship behavior: It's construct clean-up time. Human Performance, 10(2), 85-97. https://doi.org/10.1207/s15327043hup1002_2

Patel, P. C., Guedes, M. J., Bachrach, D. G., and Cho, Y. (2025). A multidimensional quiet quitting scale: Development and test of a measure of quiet quitting. PLOS ONE, 20(4), e0317624. https://doi.org/10.1371/journal.pone.0317624

Robinson, S. L., and Bennett, R. J. (1995). A typology of deviant workplace behaviors: A multidimensional scaling study. Academy of Management Journal, 38(2), 555-572. https://doi.org/10.5465/256693

Scott, J. C. (1985). Weapons of the weak: Everyday forms of peasant resistance. Yale University Press.
