# Supplementary material for: Dimensions of perceived algorithmic control and endorsement of minimum-only execution in platform delivery work
Source: Front Psychol. 2026 Oct 7;17:1937810. doi: 10.3389/fpsyg.2026.1937810 (PMC13683074; doi:10.3389/fpsyg.2026.1937810)
Supplement: Supplementary file 1 [file Data_Sheet_1.ZIP › Supplementary Appendix S4 Data and Model Reproducibility.docx]

Supplementary Appendix S4. Data and Model Reproducibility Documentation

*Manuscript ID 1937810*

This appendix documents the original Supplementary Data File 1, including its shared files, de-identification, sample derivation, and original higher-order model. The historical estimates remain available for verification. Appendix S11 retains the original higher-order results, and Appendix S12 reports the separate native dimension-level analysis introduced during revision.

# S4.1 Package contents

| **File** | **Content** | **Included in Supplementary Data File 1** | **Purpose** |
| --- | --- | --- | --- |
| item_level_data_main_model_650.csv | Randomized case ID plus 24 item responses used in the final model | Yes | Re-estimate the submitted measurement and structural model |
| data_dictionary_model_items.csv | Coding, construct membership, analytic role, and response scale | Yes | Interpret the shared variables |
| sample_derivation.csv | Derivation of N = 650 from 660 initially obtained responses | Yes | Document response-quality exclusions |
| model_specification.md | Higher-order measurement structure and all structural paths | Yes | Reconstruct the estimated model |
| participant_information_consent_and_ethics_note.md | Administered landing-page notice, reporting translation, ethics basis, and sharing scope | Yes | Document the ethical basis for the minimized file |
| deidentification_and_confidentiality_note.md | Variables removed and case-ID procedure | Yes | Document disclosure-risk reduction |
| final_smartpls_exported_results.xlsx | Complete locked final result workbook used for the manuscript | Yes | Verify reported estimates |
| machine-readable result tables | Loadings, paths, indirect associations, R-squared, reliability, HTMT, and VIF | Yes | Facilitate independent checks |
| final_model_figure.png | Final measurement and structural model figure | Yes | Visualize the model specification |
| MANIFEST_SHA256.txt | File-level SHA-256 checksums | Yes | Verify package integrity |

# S4.2 De-identification, ethics, and consent basis

- The survey was administered anonymously and did not collect names, phone numbers, email addresses, geographic coordinates, or platform-account identifiers.
- The protocol was reviewed and approved by the Ethics Committee of the School of Accounting, Tongling University (approval no. TLUIRB20260513001). Informed consent was obtained from participants and documented separately. The questionnaire landing page stated that responses would be used only for academic research, kept strictly confidential, and would not involve personally identifying information.
- For Supplementary Data File 1, the internal response identifier was removed, rows were permuted, and new study-specific case identifiers were assigned. Survey codes, response-order information, work-background variables, demographic variables, and administrative fields were excluded.
- Only the 24 item responses used in the final model are shared. The more detailed background dataset used for author-conducted supplementary analyses is not included in the public supplementary package.

# S4.3 Sample derivation

| **Stage** | **N** | **Rule or note** |
| --- | --- | --- |
| Initially obtained responses | 660 | All met the programmed eligibility conditions and passed the attention-check item. |
| Excluded for completion time below 90 seconds | 9 | Objective response-quality criterion documented in the cleaning record. |
| Excluded as a construct-level multivariate outlier | 1 | Mahalanobis and robust Mahalanobis p < .001 in the documented cleaning analysis. |
| Final analysis sample | 650 | Used in the submitted model and the locked final result workbook. |

# S4.4 Original higher-order model specification

Measurement structure

- TAC, RAC, TRC, EEX, EFD, and PC are four-item reflective first-order constructs.
- In the original specification, perceived algorithmic control (PAC) was represented as a reflective higher-order construct with TAC, RAC, and TRC as its dimensions.
- The original manuscript described a disjoint two-stage approach in which first-order scores were used as reflective indicators of PAC. This records the reported historical specification; the original SmartPLS project has not been recovered.

Structural paths

- PAC -> EEX
- PAC -> EFD
- PAC -> PC
- EEX -> EFD
- EEX -> PC
- EFD -> PC

# S4.5 Archived estimates for the original specification

| **Relationship** | **Estimate** | **STDEV** | **t** | **p** |
| --- | --- | --- | --- | --- |
| PAC -> EEX | 0.462 | 0.031 | 14.943 | < .001 |
| PAC -> EFD | 0.508 | 0.033 | 15.222 | < .001 |
| PAC -> PC | 0.347 | 0.037 | 9.352 | < .001 |
| EEX -> EFD | 0.015 | 0.036 | 0.422 | .673 |
| EEX -> PC | 0.167 | 0.036 | 4.622 | < .001 |
| EFD -> PC | 0.193 | 0.037 | 5.135 | < .001 |
| PAC -> EEX -> PC | 0.077 | 0.018 | 4.302 | < .001 |
| PAC -> EFD -> PC | 0.098 | 0.020 | 4.948 | < .001 |
| PAC -> EEX -> EFD -> PC | 0.001 | 0.003 | 0.403 | .687 |

R-squared values: EEX = 0.213; EFD = 0.265; PC = 0.324.

# S4.6 Reproducibility boundary

The historical SmartPLS project used for the original analysis has not been recovered. The original supplementary data archive supplies the exported result workbook and a documented model specification, rather than a reconstructed project presented as the historical file. The revised native six-construct PLS analysis has separate result exports and settings and is reported in Appendix S12. Its 12 structural relations and results must not be confused with the six-path higher-order specification above.

# S4.7 Frontiers supplementary-file status

Supplementary Data File 1 has been prepared for upload with the revised manuscript. The portal-assigned file label should replace the working label if Frontiers renames the file during submission.
