# Supplementary material for: Dimensions of perceived algorithmic control and endorsement of minimum-only execution in platform delivery work
Source: Front Psychol. 2026 Oct 7;17:1937810. doi: 10.3389/fpsyg.2026.1937810 (PMC13683074; doi:10.3389/fpsyg.2026.1937810)
Supplement: Supplementary file 1 [file Data_Sheet_1.ZIP › Supplementary Appendix S5 Subdimension Model.docx]

**Supplementary Appendix S5**

*Earlier dimension-level sensitivity analysis using principal-component scores*

# S5.1 Purpose and specification

This appendix retains the earlier reviewer-requested dimension-level sensitivity analysis. TAC, RAC, and TRC were entered simultaneously: EEX was regressed on the three dimensions; EFD on the three dimensions and EEX; and PC on the three dimensions, EEX, and EFD. PC denotes rule-bounded reduced discretionary effort. This score-based analysis is distinct from the native PLS analysis introduced during the present revision and reported in Appendix S12.

The six first-order blocks were represented by standardized first principal-component scores from the final N = 650 item data, followed by ordinary least squares estimation of the conditional equations. The archived analysis used 10,000 nonparametric bootstrap resamples, two-tailed tests, percentile 95% confidence intervals, and seed 20260819. The reported agreement within approximately .01 concerns the accompanying reconstruction of the original higher-order baseline; it does not make this dimension-level analysis a native SmartPLS estimate.

# S5.2 Direct associations

| **Path** | **beta** | **95% CI** | **p** | **CI excludes zero** |
| --- | --- | --- | --- | --- |
| TAC -> EEX | 0.070 | [-0.009, 0.146] | 0.082 | No |
| RAC -> EEX | 0.071 | [-0.014, 0.152] | 0.101 | No |
| TRC -> EEX | 0.412 | [0.331, 0.493] | <0.001 | Yes |
| TAC -> EFD | 0.240 | [0.159, 0.317] | <0.001 | Yes |
| RAC -> EFD | 0.164 | [0.075, 0.250] | <0.001 | Yes |
| TRC -> EFD | 0.204 | [0.115, 0.293] | <0.001 | Yes |
| EEX -> EFD | 0.025 | [-0.048, 0.099] | 0.518 | No |
| TAC -> PC | 0.341 | [0.269, 0.413] | <0.001 | Yes |
| RAC -> PC | 0.242 | [0.166, 0.317] | <0.001 | Yes |
| TRC -> PC | -0.183 | [-0.268, -0.099] | <0.001 | Yes |
| EEX -> PC | 0.233 | [0.167, 0.299] | <0.001 | Yes |
| EFD -> PC | 0.188 | [0.116, 0.260] | <0.001 | Yes |

The EEX equation explained 24.5% of variance, the EFD equation 26.0%, and the PC equation 39.2%. TAC and RAC did not have significant unique associations with EEX once TRC was included. TRC was the strongest unique predictor of EEX. All three dimensions were positively associated with EFD. TAC and RAC were positively associated with PC, whereas the conditional TRC direct coefficient was negative.

# S5.3 Indirect and total associations with PC

| **Association** | **beta** | **95% CI** | **p** | **CI excludes zero** |
| --- | --- | --- | --- | --- |
| TAC -> EEX -> PC | 0.016 | [-0.002, 0.036] | 0.082 | No |
| TAC -> EFD -> PC | 0.045 | [0.024, 0.069] | <0.001 | Yes |
| TAC -> EEX -> EFD -> PC | 0.000 | [-0.001, 0.002] | 0.564 | No |
| TAC total indirect -> PC | 0.062 | [0.034, 0.093] | <0.001 | Yes |
| TAC total association -> PC | 0.403 | [0.331, 0.473] | <0.001 | Yes |
| RAC -> EEX -> PC | 0.017 | [-0.003, 0.037] | 0.101 | No |
| RAC -> EFD -> PC | 0.031 | [0.013, 0.052] | <0.001 | Yes |
| RAC -> EEX -> EFD -> PC | 0.000 | [-0.001, 0.002] | 0.558 | No |
| RAC total indirect -> PC | 0.048 | [0.020, 0.075] | <0.001 | Yes |
| RAC total association -> PC | 0.290 | [0.213, 0.364] | <0.001 | Yes |
| TRC -> EEX -> PC | 0.096 | [0.064, 0.133] | <0.001 | Yes |
| TRC -> EFD -> PC | 0.038 | [0.017, 0.065] | <0.001 | Yes |
| TRC -> EEX -> EFD -> PC | 0.002 | [-0.004, 0.008] | 0.518 | No |
| TRC total indirect -> PC | 0.136 | [0.099, 0.179] | <0.001 | Yes |
| TRC total association -> PC | -0.046 | [-0.125, 0.031] | 0.239 | No |

The archived TRC–PC direct coefficient was −0.182685, the sum of its indirect products was +0.136408, and their total was −0.046277 (95% CI [−0.125484, 0.031498], p = .239). The zero-order TRC–PC score correlation was positive (r = .313875). A separate check of PC regressed on TAC, RAC, and TRC yielded the same −0.046277 coefficient, showing that the sign had already changed after conditioning on the other control dimensions. These quantities do not decompose the zero-order correlation, identify a unique suppressor, or demonstrate a protective total association of trajectory monitoring.

# S5.4 Explained variance and collinearity

| **Endogenous construct** | **R-squared** | **Bootstrap 95% interval** |
| --- | --- | --- |
| EEX | 0.245 | [0.191, 0.307] |
| EFD | 0.260 | [0.209, 0.319] |
| PC | 0.392 | [0.338, 0.452] |

| **Equation** | **Predictor** | **VIF** |
| --- | --- | --- |
| EEX | TAC | 1.426 |
| EEX | RAC | 1.538 |
| EEX | TRC | 1.610 |
| EFD | TAC | 1.432 |
| EFD | RAC | 1.545 |
| EFD | TRC | 1.834 |
| EFD | EEX | 1.324 |
| PC | TAC | 1.510 |
| PC | RAC | 1.581 |
| PC | TRC | 1.891 |
| PC | EEX | 1.325 |
| PC | EFD | 1.352 |

All VIF values were below 1.90. The coefficients nevertheless describe conditional associations among correlated control dimensions in the same platform setting; low VIF does not establish that they share one reflective higher-order explanation.

# S5.5 Zero-order correlations

| **Construct** | **TAC** | **RAC** | **TRC** | **EEX** | **EFD** | **PC** |
| --- | --- | --- | --- | --- | --- | --- |
| TAC | 1.000 | 0.462 | 0.498 | 0.308 | 0.425 | 0.514 |
| RAC | 0.462 | 1.000 | 0.551 | 0.330 | 0.395 | 0.450 |
| TRC | 0.498 | 0.551 | 1.000 | 0.486 | 0.426 | 0.314 |
| EEX | 0.308 | 0.330 | 0.486 | 1.000 | 0.252 | 0.377 |
| EFD | 0.425 | 0.395 | 0.426 | 0.252 | 1.000 | 0.409 |
| PC | 0.514 | 0.450 | 0.314 | 0.377 | 0.409 | 1.000 |

# S5.6 Interpretation boundary

The disaggregated model answers a limited question: whether the higher-order pattern rests only on the workload-like temporal block. It does not. TRC had a strong unique association with exhaustion and a positive unique association with emotional feigning; RAC also contributed beyond TAC. The model does not, however, identify an effect unique to artificial intelligence. The survey did not include objective dispatch logs, assigned deadlines, penalty records, a comparison group managed by a human dispatcher, a separate measure of general work pressure, or items explicitly capturing automatic penalties and the absence of human appeal. The supported claim is therefore limited to workers' perceived control in an algorithmically managed platform-delivery setting.

The saved bootstrap results support the reported marginal intervals. Rows across independently resampled statistics in the historical output must not be treated as jointly paired parameter draws for new coefficient-difference or decomposition tests. The native PLS estimates and their intervals in Appendix S12 retain a separate provenance.
