# Supplementary material for: Dimensions of perceived algorithmic control and endorsement of minimum-only execution in platform delivery work
Source: Front Psychol. 2026 Oct 7;17:1937810. doi: 10.3389/fpsyg.2026.1937810 (PMC13683074; doi:10.3389/fpsyg.2026.1937810)
Supplement: Supplementary file 1 [file Data_Sheet_1.ZIP › Supplementary Appendix S6 Common Method Variance.docx]

**Supplementary Appendix S6**

**Common method variance diagnostics and method-factor sensitivity analysis**

# **S6.1 Purpose, data, and interpretation boundary**

All focal variables were reported by the same respondent in one cross-sectional questionnaire. In response to Reviewer 1, this appendix replaces reliance on a single Harman threshold with a multi-part revision-stage diagnostic based on the locked final item-level dataset (N = 650; 24 final-model items). The analyses include the complete unrotated principal-component solution, a six-factor confirmatory factor model, an unmeasured latent common method factor (ULMC) model, variance decomposition, and a method-adjusted structural sensitivity analysis. These post hoc diagnostics assess whether a simple common response tendency can reproduce the reported pattern; they cannot establish that common method variance is absent or substitute for temporal separation, a marker variable, or an independent outcome source.

The ULMC diagnostic was estimated as a covariance-based confirmatory factor model. Each item loaded on its intended substantive factor and on one common method factor that was constrained to be orthogonal to the six substantive factors. The method factor variance was fixed to one and its item loadings were freely estimated. Because the performance of post hoc CMV procedures depends on their assumptions, and PLS implementations of the ULMC technique have been criticized, this analysis is treated as a sensitivity diagnostic rather than as a correction to the locked PLS-SEM model (Podsakoff et al., 2003; Richardson et al., 2009; Chin et al., 2012).

This appendix retains the earlier common-method diagnostic and the aggregate-PAC structural sensitivity analysis. The CFA comparison here concerns a method-factor specification; it is distinct from the later common-PAC restriction diagnostic in Appendix S11 and from the native six-construct PLS estimates in Appendix S12.

# **S6.2 Complete unrotated principal-component solution**

The final N = 650 analysis yielded five components with eigenvalues greater than one. The first component accounted for 32.186% of total variance. The 32.262% value in the submitted manuscript was traced to the pre-exclusion N = 660 file and has been corrected. The complete eigenvalue and variance solution is reported below; loadings for the five retained components follow in Table S6B.

**Table S6A. Full unrotated principal-component eigenvalue and variance solution**

| **Component** | **Eigenvalue** | **Variance (%)** | **Cumulative (%)** | **Eigenvalue > 1** |
| --- | --- | --- | --- | --- |
| 1 | 7.725 | 32.186 | 32.186 | Yes |
| 2 | 2.022 | 8.425 | 40.611 | Yes |
| 3 | 1.685 | 7.020 | 47.630 | Yes |
| 4 | 1.554 | 6.475 | 54.106 | Yes |
| 5 | 1.299 | 5.414 | 59.519 | Yes |
| 6 | 0.811 | 3.380 | 62.899 | No |
| 7 | 0.658 | 2.743 | 65.642 | No |
| 8 | 0.629 | 2.621 | 68.264 | No |
| 9 | 0.622 | 2.593 | 70.857 | No |
| 10 | 0.581 | 2.419 | 73.275 | No |
| 11 | 0.561 | 2.336 | 75.611 | No |
| 12 | 0.558 | 2.326 | 77.937 | No |
| 13 | 0.525 | 2.187 | 80.124 | No |
| 14 | 0.505 | 2.103 | 82.227 | No |
| 15 | 0.491 | 2.044 | 84.272 | No |
| 16 | 0.475 | 1.981 | 86.252 | No |
| 17 | 0.459 | 1.912 | 88.165 | No |
| 18 | 0.456 | 1.899 | 90.063 | No |
| 19 | 0.438 | 1.825 | 91.889 | No |
| 20 | 0.416 | 1.734 | 93.623 | No |
| 21 | 0.400 | 1.668 | 95.291 | No |
| 22 | 0.388 | 1.618 | 96.908 | No |
| 23 | 0.379 | 1.580 | 98.488 | No |
| 24 | 0.363 | 1.512 | 100.000 | No |

**Table S6B. Unrotated item loadings for the five components with eigenvalues greater than one**

| **Item** | **Component 1** | **Component 2** | **Component 3** | **Component 4** | **Component 5** |
| --- | --- | --- | --- | --- | --- |
| TAC1 | 0.588 | -0.159 | 0.124 | -0.197 | 0.420 |
| TAC2 | 0.594 | -0.181 | 0.156 | -0.231 | 0.385 |
| TAC3 | 0.623 | -0.187 | 0.125 | -0.170 | 0.375 |
| TAC4 | 0.596 | -0.158 | 0.112 | -0.262 | 0.368 |
| RAC1 | 0.603 | 0.027 | -0.090 | -0.314 | -0.375 |
| RAC2 | 0.590 | 0.011 | -0.075 | -0.330 | -0.372 |
| RAC3 | 0.604 | -0.012 | -0.160 | -0.321 | -0.336 |
| RAC4 | 0.554 | 0.009 | -0.128 | -0.281 | -0.370 |
| TRC1 | 0.626 | 0.244 | -0.332 | -0.091 | 0.175 |
| TRC2 | 0.656 | 0.223 | -0.287 | -0.094 | 0.122 |
| TRC3 | 0.620 | 0.291 | -0.327 | -0.073 | 0.149 |
| TRC4 | 0.622 | 0.268 | -0.300 | -0.088 | 0.040 |
| EEX1 | 0.517 | 0.445 | 0.187 | 0.245 | 0.052 |
| EEX2 | 0.418 | 0.526 | 0.175 | 0.317 | -0.020 |
| EEX3 | 0.504 | 0.460 | 0.216 | 0.257 | 0.042 |
| EEX4 | 0.467 | 0.528 | 0.142 | 0.295 | -0.061 |
| EFD1 | 0.525 | -0.399 | -0.227 | 0.424 | -0.014 |
| EFD2 | 0.579 | -0.364 | -0.209 | 0.379 | -0.085 |
| EFD3 | 0.568 | -0.329 | -0.228 | 0.399 | -0.011 |
| EFD4 | 0.569 | -0.380 | -0.316 | 0.375 | -0.044 |
| PC1 | 0.525 | -0.135 | 0.459 | 0.076 | -0.168 |
| PC2 | 0.521 | -0.235 | 0.399 | 0.032 | -0.160 |
| PC3 | 0.564 | -0.099 | 0.457 | 0.018 | -0.124 |
| PC4 | 0.520 | -0.211 | 0.462 | -0.015 | -0.119 |

# **S6.3 Six-factor CFA and ULMC model comparison**

Both the correlated six-factor CFA and the ULMC model showed low residual misfit. Adding the method factor reduced chi-square by 40.831 with 24 additional parameters (p = .017), but the complexity-adjusted AIC and BIC did not favor the ULMC specification. The comparison is therefore not interpreted as proof that a single common method factor explains the substantive associations.

**Table S6C. Fit comparison for the substantive CFA and ULMC model**

| **Model** | **Free par.** | **Chi-square** | **df** | **p** | **CFI** | **TLI** | **RMSEA** | **SRMR** | **AIC*** | **BIC*** |
| --- | --- | --- | --- | --- | --- | --- | --- | --- | --- | --- |
| Six-factor CFA | 63 | 203.698 | 237 | 0.943 | 1.000 | 1.000 | 0.000 | 0.024 | 329.698 | 611.748 |
| Six-factor CFA + ULMC | 87 | 162.867 | 213 | 0.996 | 1.000 | 1.000 | 0.000 | 0.019 | 336.867 | 726.363 |

*Note. *Relative information criteria computed from the same observed covariance matrix. ULMC vs. six-factor CFA: delta chi-square(24) = 40.831, p = .017.*

# **S6.4 Substantive, method, and residual variance**

Across the 24 items, the average variance attributed to substantive factors was 44.557%, average method variance was 6.429%, and average residual variance was 49.015%. Nine of the 24 method loadings were statistically different from zero at p < .05. Method variance was heterogeneous across item blocks, ranging from 1.682% for TRC to 12.808% for TAC, rather than operating as a uniform elevation across all constructs.

**Table S6D. Variance decomposition by construct block**

| **Scope** | **Substantive (%)** | **Method (%)** | **Residual (%)** | **Method/substantive** | **Method loadings p < .05** | **Items** |
| --- | --- | --- | --- | --- | --- | --- |
| All 24 items | 44.557 | 6.429 | 49.015 | 0.144 | 9 | 24 |
| EEX | 45.455 | 2.493 | 52.053 | 0.055 | 1 | 4 |
| EFD | 47.522 | 8.860 | 43.618 | 0.186 | 3 | 4 |
| PC | 39.114 | 7.577 | 53.309 | 0.194 | 1 | 4 |
| RAC | 43.603 | 5.152 | 51.245 | 0.118 | 1 | 4 |
| TAC | 38.811 | 12.808 | 48.380 | 0.330 | 3 | 4 |
| TRC | 52.835 | 1.682 | 45.483 | 0.032 | 0 | 4 |

**Table S6E. Item-level substantive and method loadings**

| **Item** | **Construct** | **Substantive loading** | **Method loading** | **Substantive var. (%)** | **Method var. (%)** | **Residual var. (%)** | **z** | **p** |
| --- | --- | --- | --- | --- | --- | --- | --- | --- |
| TAC1 | TAC | 0.598 | 0.353 | 35.80 | 12.45 | 51.75 | 3.262 | 0.001 |
| TAC2 | TAC | 0.627 | 0.333 | 39.33 | 11.06 | 49.60 | 3.211 | 0.001 |
| TAC3 | TAC | 0.578 | 0.476 | 33.38 | 22.63 | 43.99 | 4.619 | <.001 |
| TAC4 | TAC | 0.684 | 0.226 | 46.73 | 5.10 | 48.17 | 1.731 | 0.083 |
| RAC1 | RAC | 0.695 | 0.210 | 48.35 | 4.41 | 47.24 | 1.520 | 0.128 |
| RAC2 | RAC | 0.660 | 0.230 | 43.60 | 5.27 | 51.13 | 1.303 | 0.192 |
| RAC3 | RAC | 0.676 | 0.258 | 45.68 | 6.67 | 47.65 | 2.128 | 0.033 |
| RAC4 | RAC | 0.607 | 0.206 | 36.79 | 4.26 | 58.96 | 1.507 | 0.132 |
| TRC1 | TRC | 0.727 | 0.135 | 52.92 | 1.82 | 45.26 | 1.415 | 0.157 |
| TRC2 | TRC | 0.717 | 0.183 | 51.44 | 3.34 | 45.22 | 1.895 | 0.058 |
| TRC3 | TRC | 0.766 | 0.035 | 58.60 | 0.12 | 41.28 | 0.318 | 0.750 |
| TRC4 | TRC | 0.696 | 0.120 | 48.38 | 1.45 | 50.17 | 1.011 | 0.312 |
| EEX1 | EEX | 0.662 | 0.234 | 43.85 | 5.47 | 50.68 | 2.459 | 0.014 |
| EEX2 | EEX | 0.667 | 0.041 | 44.47 | 0.17 | 55.36 | 0.284 | 0.777 |
| EEX3 | EEX | 0.665 | 0.201 | 44.28 | 4.05 | 51.67 | 1.881 | 0.060 |
| EEX4 | EEX | 0.702 | 0.053 | 49.21 | 0.28 | 50.51 | 0.340 | 0.734 |
| EFD1 | EFD | 0.685 | 0.251 | 46.96 | 6.31 | 46.73 | 2.743 | 0.006 |
| EFD2 | EFD | 0.688 | 0.298 | 47.34 | 8.86 | 43.80 | 1.783 | 0.075 |
| EFD3 | EFD | 0.641 | 0.355 | 41.03 | 12.57 | 46.40 | 2.976 | 0.003 |
| EFD4 | EFD | 0.740 | 0.278 | 54.76 | 7.71 | 37.54 | 2.566 | 0.010 |
| PC1 | PC | 0.607 | 0.238 | 36.89 | 5.65 | 57.47 | 1.026 | 0.305 |
| PC2 | PC | 0.526 | 0.376 | 27.67 | 14.14 | 58.19 | 2.538 | 0.011 |
| PC3 | PC | 0.764 | 0.098 | 58.37 | 0.97 | 40.67 | 0.778 | 0.437 |
| PC4 | PC | 0.579 | 0.309 | 33.53 | 9.55 | 56.92 | 1.753 | 0.080 |

# **S6.5 Method-adjusted structural sensitivity**

To assess whether the principal pattern survived adjustment for the estimated method factor, the focal structural equations were recomputed from the ULMC-adjusted substantive latent correlation matrix. The adjusted estimates are revision-stage sensitivity results and do not replace the locked SmartPLS coefficients. The positive PAC associations with EEX, EFD, and PC remained, as did the positive EEX-PC and EFD-PC associations. The EEX-EFD path remained close to zero and nonsignificant, which is inconsistent with the simplest account in which one uniform response tendency inflates every focal association in the same direction.

**Table S6F. ULMC-adjusted structural sensitivity estimates**

| **Association** | **Estimate** | **SE** | **z** | **p** | **95% CI lower** | **95% CI upper** |
| --- | --- | --- | --- | --- | --- | --- |
| PAC -> EEX | 0.517 | 0.060 | 8.553 | <.001 | 0.398 | 0.635 |
| PAC -> EFD | 0.517 | 0.071 | 7.243 | <.001 | 0.377 | 0.656 |
| EEX -> EFD | -0.009 | 0.063 | -0.148 | 0.883 | -0.133 | 0.115 |
| PAC -> PC | 0.361 | 0.064 | 5.667 | <.001 | 0.236 | 0.485 |
| EEX -> PC | 0.228 | 0.055 | 4.107 | <.001 | 0.119 | 0.336 |
| EFD -> PC | 0.174 | 0.072 | 2.409 | 0.016 | 0.032 | 0.316 |
| PAC -> EEX -> PC | 0.118 | 0.033 | 3.596 | <.001 | 0.054 | 0.182 |
| PAC -> EFD -> PC | 0.090 | 0.044 | 2.059 | 0.039 | 0.004 | 0.176 |
| PAC -> EEX -> EFD -> PC | -0.001 | 0.006 | -0.150 | 0.881 | -0.012 | 0.010 |
| R2 EEX | 0.267 | 0.062 | 4.277 | <.001 | 0.145 | 0.389 |
| R2 EFD | 0.262 | 0.072 | 3.624 | <.001 | 0.120 | 0.404 |
| R2 PC | 0.382 | 0.093 | 4.126 | <.001 | 0.200 | 0.563 |

# **S6.6 Interpretation boundary**

The evidence does not support the claim that common method variance is absent. All focal variables remain same-source, self-reported, and cross-sectional; the original survey did not include a marker variable, temporal separation, or an external behavioral criterion. The full unrotated solution, heterogeneous item-level method loadings, modest average method variance, method-adjusted structural sensitivity, and near-zero EEX-EFD association jointly argue against the narrowest explanation that one uniform response tendency produced all reported relationships. They do not rule out more selective response styles, negative affectivity, social desirability, recall processes, or other method-related influences.

# **References**

Chin, W. W., Thatcher, J. B., and Wright, R. T. (2012). Assessing common method bias: Problems with the ULMC technique. MIS Quarterly, 36(3), 1003-1019. doi: 10.2307/41703491

Podsakoff, P. M., MacKenzie, S. B., Lee, J.-Y., and Podsakoff, N. P. (2003). Common method biases in behavioral research: A critical review of the literature and recommended remedies. Journal of Applied Psychology, 88(5), 879-903. doi: 10.1037/0021-9010.88.5.879

Richardson, H. A., Simmering, M. J., and Sturman, M. C. (2009). A tale of three perspectives: Examining post hoc statistical techniques for detection and correction of common method variance. Organizational Research Methods, 12(4), 762-800. doi: 10.1177/1094428109332834
