# Supplementary material for: Dimensions of perceived algorithmic control and endorsement of minimum-only execution in platform delivery work
Source: Front Psychol. 2026 Oct 7;17:1937810. doi: 10.3389/fpsyg.2026.1937810 (PMC13683074; doi:10.3389/fpsyg.2026.1937810)
Supplement: Supplementary file 1 [file Data_Sheet_1.ZIP › Supplementary Appendix S7 Control and Work Mode.docx]

**Supplementary Appendix S7**

*Control-adjusted and work-mode-stratified sensitivity models*

# S7.1 Purpose, data, and model specification

This appendix retains the earlier control-adjusted and work-mode-stratified sensitivity analyses based on the final N = 650 dataset. They use the aggregate PAC score reconstruction described below. They do not constitute controlled or subgroup re-estimation of the revised native six-construct PLS model.

In the full-sample model, work mode was represented by part-time and occasional/temporary indicators, with full-time workers as the reference group. Gender, age group, delivery tenure, and daily working hours were also included. All controls were entered in the EEX, EFD, and PC equations. In the stratified models, age, gender, tenure, and hours were retained within each work-mode group. Reflective first-order construct scores were estimated as standardized first principal-component scores, and reflective higher-order PAC was estimated from TAC, RAC, and TRC scores. Inference used 10,000 nonparametric bootstrap samples, two-tailed sign-proportion p values, percentile 95% confidence intervals, and random seed 20260819.

The reconstruction reproduced the locked SmartPLS baseline coefficients within 0.011. These analyses are sensitivity checks rather than a recovery of the historical SmartPLS project. The group-specific results are reported directly; exploratory pairwise contrasts are not presented as formal MICOM-based PLS-MGA tests.

# S7.2 Sample composition and descriptive differences

Table S7A. Sample composition and pooled-score descriptive statistics by work mode

| **Work mode** | **N** | **Percent** | **PAC mean (pooled z)** | **PAC SD (pooled z)** | **EEX mean (pooled z)** | **EEX SD (pooled z)** | **EFD mean (pooled z)** | **EFD SD (pooled z)** | **PC mean (pooled z)** | **PC SD (pooled z)** |
| --- | --- | --- | --- | --- | --- | --- | --- | --- | --- | --- |
| Full-time | 299 | 46.000 | -0.532 | 0.960 | -0.278 | 0.989 | -0.371 | 0.995 | -0.364 | 0.998 |
| Part-time | 215 | 33.077 | 0.248 | 0.760 | 0.063 | 0.930 | 0.184 | 0.932 | 0.208 | 0.911 |
| Occasional/temporary | 136 | 20.923 | 0.778 | 0.727 | 0.512 | 0.918 | 0.526 | 0.783 | 0.471 | 0.844 |

# S7.3 Baseline reproduction and full-sample control-adjusted model

Table S7B. Locked SmartPLS baseline and revision-stage reconstruction

| **Association** | **Locked SmartPLS** | **Revision reconstruction** | **Difference** | **Absolute difference** |
| --- | --- | --- | --- | --- |
| PAC -> EEX | 0.462 | 0.460 | -0.002 | 0.002 |
| PAC -> EFD | 0.508 | 0.497 | -0.011 | 0.011 |
| EEX -> EFD | 0.015 | 0.024 | 0.009 | 0.009 |
| PAC -> PC | 0.347 | 0.341 | -0.006 | 0.006 |
| EEX -> PC | 0.167 | 0.171 | 0.004 | 0.004 |
| EFD -> PC | 0.193 | 0.193 | -0.000 | 0.000 |
| PAC -> EEX -> PC | 0.077 | 0.079 | 0.002 | 0.002 |
| PAC -> EFD -> PC | 0.098 | 0.096 | -0.002 | 0.002 |
| PAC -> EEX -> EFD -> PC | 0.001 | 0.002 | 0.001 | 0.001 |
| R2 EEX | 0.213 | 0.211 | -0.002 | 0.002 |
| R2 EFD | 0.265 | 0.258 | -0.007 | 0.007 |
| R2 PC | 0.324 | 0.320 | -0.004 | 0.004 |
| PAC -> TAC | 0.792 | 0.792 | -0.000 | 0.000 |
| PAC -> RAC | 0.821 | 0.822 | 0.001 | 0.001 |
| PAC -> TRC | 0.837 | 0.840 | 0.003 | 0.003 |

Table S7C. Full-sample control-adjusted focal paths and indirect associations

| **Association** | **Estimate** | **Bootstrap mean** | **Bootstrap SE** | **CI 2.5%** | **CI 97.5%** | **Two-tailed p** |
| --- | --- | --- | --- | --- | --- | --- |
| PAC -> EEX | 0.193 | 0.194 | 0.052 | 0.092 | 0.295 | <.001 |
| PAC -> EFD | 0.223 | 0.220 | 0.052 | 0.118 | 0.322 | <.001 |
| EEX -> EFD | -0.041 | -0.041 | 0.037 | -0.113 | 0.031 | 0.258 |
| PAC -> PC | 0.210 | 0.210 | 0.050 | 0.112 | 0.308 | <.001 |
| EEX -> PC | 0.138 | 0.138 | 0.037 | 0.065 | 0.210 | <.001 |
| EFD -> PC | 0.149 | 0.149 | 0.039 | 0.072 | 0.227 | <.001 |
| PAC -> EEX -> PC | 0.027 | 0.027 | 0.011 | 0.009 | 0.050 | <.001 |
| PAC -> EFD -> PC | 0.033 | 0.033 | 0.012 | 0.012 | 0.059 | <.001 |
| PAC -> EEX -> EFD -> PC | -0.001 | -0.001 | 0.001 | -0.004 | 0.001 | 0.258 |
| PAC total indirect -> PC | 0.059 | 0.058 | 0.015 | 0.031 | 0.092 | <.001 |
| PAC total association -> PC | 0.269 | 0.268 | 0.051 | 0.169 | 0.367 | <.001 |

Table S7D. Full-sample control-variable paths

| **Association** | **Estimate** | **Bootstrap mean** | **Bootstrap SE** | **CI 2.5%** | **CI 97.5%** | **Two-tailed p** |
| --- | --- | --- | --- | --- | --- | --- |
| Female -> EEX | 0.019 | 0.019 | 0.036 | -0.052 | 0.090 | 0.598 |
| Age group -> EEX | 0.115 | 0.115 | 0.044 | 0.030 | 0.201 | 0.009 |
| Delivery tenure -> EEX | 0.107 | 0.108 | 0.043 | 0.021 | 0.191 | 0.014 |
| Daily hours -> EEX | 0.175 | 0.175 | 0.042 | 0.092 | 0.257 | <.001 |
| Part-time (vs full-time) -> EEX | -0.013 | -0.012 | 0.038 | -0.088 | 0.063 | 0.755 |
| Occasional/temporary (vs full-time) -> EEX | 0.071 | 0.071 | 0.040 | -0.008 | 0.149 | 0.078 |
| Female -> EFD | 0.080 | 0.081 | 0.037 | 0.006 | 0.152 | 0.034 |
| Age group -> EFD | 0.133 | 0.133 | 0.040 | 0.055 | 0.210 | 0.001 |
| Delivery tenure -> EFD | 0.133 | 0.134 | 0.042 | 0.051 | 0.216 | 0.002 |
| Daily hours -> EFD | 0.100 | 0.100 | 0.041 | 0.021 | 0.179 | 0.014 |
| Part-time (vs full-time) -> EFD | 0.085 | 0.085 | 0.040 | 0.007 | 0.162 | 0.034 |
| Occasional/temporary (vs full-time) -> EFD | 0.103 | 0.103 | 0.041 | 0.023 | 0.183 | 0.012 |
| Female -> PC | 0.013 | 0.014 | 0.034 | -0.052 | 0.081 | 0.687 |
| Age group -> PC | 0.088 | 0.088 | 0.038 | 0.014 | 0.162 | 0.019 |
| Delivery tenure -> PC | 0.104 | 0.105 | 0.040 | 0.026 | 0.182 | 0.009 |
| Daily hours -> PC | 0.038 | 0.038 | 0.045 | -0.050 | 0.126 | 0.395 |
| Part-time (vs full-time) -> PC | 0.074 | 0.074 | 0.039 | -0.004 | 0.149 | 0.061 |
| Occasional/temporary (vs full-time) -> PC | 0.042 | 0.041 | 0.041 | -0.040 | 0.122 | 0.321 |

Table S7E. R-squared comparison

| **Endogenous construct** | **Locked SmartPLS R2** | **Reconstructed baseline R2** | **Control-adjusted R2** | **Increment vs reconstructed baseline** | **Control-adjusted adjusted R2** |
| --- | --- | --- | --- | --- | --- |
| EEX | 0.213 | 0.211 | 0.266 | 0.055 | 0.258 |
| EFD | 0.265 | 0.258 | 0.316 | 0.057 | 0.307 |
| PC | 0.324 | 0.320 | 0.340 | 0.020 | 0.331 |

Table S7F. Inner VIF values for the control-adjusted model

| **Equation** | **Predictor** | **VIF** |
| --- | --- | --- |
| EEX | PAC | 2.549 |
| EEX | Female | 1.289 |
| EEX | Age group | 1.567 |
| EEX | Delivery tenure | 1.588 |
| EEX | Daily hours | 1.596 |
| EEX | Part-time (vs full-time) | 1.351 |
| EEX | Occasional/temporary (vs full-time) | 1.579 |
| EFD | PAC | 2.600 |
| EFD | EEX | 1.362 |
| EFD | Female | 1.289 |
| EFD | Age group | 1.585 |
| EFD | Delivery tenure | 1.604 |
| EFD | Daily hours | 1.638 |
| EFD | Part-time (vs full-time) | 1.352 |
| EFD | Occasional/temporary (vs full-time) | 1.586 |
| PC | PAC | 2.673 |
| PC | EEX | 1.365 |
| PC | EFD | 1.461 |
| PC | Female | 1.299 |
| PC | Age group | 1.611 |
| PC | Delivery tenure | 1.630 |
| PC | Daily hours | 1.652 |
| PC | Part-time (vs full-time) | 1.362 |
| PC | Occasional/temporary (vs full-time) | 1.601 |

# S7.4 Work-mode-stratified models

Table S7G. Control-adjusted structural paths by work mode

| **Work mode** | **N** | **Path** | **Estimate** | **CI 2.5%** | **CI 97.5%** | **Two-tailed p** |
| --- | --- | --- | --- | --- | --- | --- |
| Full-time | 299 | PAC -> EEX | 0.155 | 0.008 | 0.299 | 0.038 |
| Full-time | 299 | PAC -> EFD | 0.323 | 0.175 | 0.460 | <.001 |
| Full-time | 299 | EEX -> EFD | -0.026 | -0.134 | 0.082 | 0.612 |
| Full-time | 299 | PAC -> PC | 0.224 | 0.087 | 0.356 | 0.001 |
| Full-time | 299 | EEX -> PC | 0.107 | 0.003 | 0.209 | 0.043 |
| Full-time | 299 | EFD -> PC | 0.240 | 0.133 | 0.347 | <.001 |
| Part-time | 215 | PAC -> EEX | 0.213 | 0.075 | 0.348 | 0.002 |
| Part-time | 215 | PAC -> EFD | 0.000 | -0.158 | 0.153 | 0.988 |
| Part-time | 215 | EEX -> EFD | 0.017 | -0.107 | 0.138 | 0.810 |
| Part-time | 215 | PAC -> PC | 0.187 | 0.027 | 0.346 | 0.021 |
| Part-time | 215 | EEX -> PC | 0.230 | 0.096 | 0.361 | 0.002 |
| Part-time | 215 | EFD -> PC | 0.119 | -0.010 | 0.243 | 0.075 |
| Occasional/temporary | 136 | PAC -> EEX | 0.154 | -0.041 | 0.357 | 0.121 |
| Occasional/temporary | 136 | PAC -> EFD | 0.266 | 0.059 | 0.455 | 0.011 |
| Occasional/temporary | 136 | EEX -> EFD | -0.160 | -0.342 | 0.031 | 0.100 |
| Occasional/temporary | 136 | PAC -> PC | 0.096 | -0.105 | 0.281 | 0.348 |
| Occasional/temporary | 136 | EEX -> PC | -0.001 | -0.173 | 0.165 | 0.985 |
| Occasional/temporary | 136 | EFD -> PC | -0.100 | -0.251 | 0.056 | 0.212 |

Table S7H. Specific and total indirect associations by work mode

| **Work mode** | **N** | **Indirect association** | **Estimate** | **CI 2.5%** | **CI 97.5%** | **Two-tailed p** |
| --- | --- | --- | --- | --- | --- | --- |
| Full-time | 299 | PAC -> EEX -> PC | 0.017 | -0.001 | 0.045 | 0.076 |
| Full-time | 299 | PAC -> EFD -> PC | 0.078 | 0.033 | 0.133 | <.001 |
| Full-time | 299 | PAC -> EEX -> EFD -> PC | -0.001 | -0.006 | 0.004 | 0.631 |
| Full-time | 299 | PAC total indirect -> PC | 0.093 | 0.044 | 0.155 | <.001 |
| Full-time | 299 | PAC total association -> PC | 0.317 | 0.176 | 0.449 | <.001 |
| Part-time | 215 | PAC -> EEX -> PC | 0.049 | 0.012 | 0.098 | 0.004 |
| Part-time | 215 | PAC -> EFD -> PC | 0.000 | -0.025 | 0.020 | 0.976 |
| Part-time | 215 | PAC -> EEX -> EFD -> PC | 0.000 | -0.003 | 0.005 | 0.818 |
| Part-time | 215 | PAC total indirect -> PC | 0.049 | 0.006 | 0.099 | 0.024 |
| Part-time | 215 | PAC total association -> PC | 0.237 | 0.073 | 0.398 | 0.006 |
| Occasional/temporary | 136 | PAC -> EEX -> PC | -0.000 | -0.036 | 0.034 | 0.998 |
| Occasional/temporary | 136 | PAC -> EFD -> PC | -0.027 | -0.076 | 0.016 | 0.224 |
| Occasional/temporary | 136 | PAC -> EEX -> EFD -> PC | 0.002 | -0.002 | 0.011 | 0.377 |
| Occasional/temporary | 136 | PAC total indirect -> PC | -0.024 | -0.079 | 0.030 | 0.364 |
| Occasional/temporary | 136 | PAC total association -> PC | 0.071 | -0.111 | 0.247 | 0.440 |

Table S7I. Control-variable paths within work-mode groups

| **Work mode** | **N** | **Control path** | **Estimate** | **CI 2.5%** | **CI 97.5%** | **Two-tailed p** |
| --- | --- | --- | --- | --- | --- | --- |
| Full-time | 299 | Female -> EEX | 0.078 | -0.027 | 0.180 | 0.142 |
| Full-time | 299 | Age group -> EEX | 0.051 | -0.087 | 0.185 | 0.466 |
| Full-time | 299 | Delivery tenure -> EEX | 0.175 | 0.054 | 0.296 | 0.005 |
| Full-time | 299 | Daily hours -> EEX | 0.149 | 0.021 | 0.275 | 0.023 |
| Full-time | 299 | Female -> EFD | 0.042 | -0.078 | 0.161 | 0.491 |
| Full-time | 299 | Age group -> EFD | 0.107 | -0.011 | 0.233 | 0.081 |
| Full-time | 299 | Delivery tenure -> EFD | 0.086 | -0.036 | 0.211 | 0.160 |
| Full-time | 299 | Daily hours -> EFD | 0.071 | -0.048 | 0.188 | 0.244 |
| Full-time | 299 | Female -> PC | 0.069 | -0.030 | 0.167 | 0.173 |
| Full-time | 299 | Age group -> PC | 0.056 | -0.060 | 0.168 | 0.333 |
| Full-time | 299 | Delivery tenure -> PC | 0.111 | -0.007 | 0.229 | 0.066 |
| Full-time | 299 | Daily hours -> PC | -0.072 | -0.195 | 0.060 | 0.270 |
| Part-time | 215 | Female -> EEX | -0.080 | -0.210 | 0.053 | 0.240 |
| Part-time | 215 | Age group -> EEX | 0.117 | -0.027 | 0.253 | 0.100 |
| Part-time | 215 | Delivery tenure -> EEX | 0.040 | -0.104 | 0.184 | 0.595 |
| Part-time | 215 | Daily hours -> EEX | 0.189 | 0.049 | 0.325 | 0.009 |
| Part-time | 215 | Female -> EFD | 0.115 | -0.016 | 0.246 | 0.083 |
| Part-time | 215 | Age group -> EFD | 0.264 | 0.131 | 0.394 | <.001 |
| Part-time | 215 | Delivery tenure -> EFD | 0.134 | -0.009 | 0.276 | 0.066 |
| Part-time | 215 | Daily hours -> EFD | 0.112 | -0.017 | 0.250 | 0.096 |
| Part-time | 215 | Female -> PC | -0.027 | -0.141 | 0.091 | 0.648 |
| Part-time | 215 | Age group -> PC | 0.085 | -0.046 | 0.212 | 0.194 |
| Part-time | 215 | Delivery tenure -> PC | -0.037 | -0.169 | 0.096 | 0.592 |
| Part-time | 215 | Daily hours -> PC | 0.147 | 0.004 | 0.295 | 0.046 |
| Occasional/temporary | 136 | Female -> EEX | 0.064 | -0.109 | 0.223 | 0.477 |
| Occasional/temporary | 136 | Age group -> EEX | 0.236 | 0.058 | 0.416 | 0.009 |
| Occasional/temporary | 136 | Delivery tenure -> EEX | 0.042 | -0.142 | 0.225 | 0.612 |
| Occasional/temporary | 136 | Daily hours -> EEX | 0.232 | 0.050 | 0.403 | 0.010 |
| Occasional/temporary | 136 | Female -> EFD | 0.135 | -0.025 | 0.294 | 0.096 |
| Occasional/temporary | 136 | Age group -> EFD | 0.012 | -0.164 | 0.192 | 0.885 |
| Occasional/temporary | 136 | Delivery tenure -> EFD | 0.206 | 0.027 | 0.372 | 0.024 |
| Occasional/temporary | 136 | Daily hours -> EFD | 0.074 | -0.099 | 0.248 | 0.407 |
| Occasional/temporary | 136 | Female -> PC | -0.018 | -0.176 | 0.163 | 0.871 |
| Occasional/temporary | 136 | Age group -> PC | 0.209 | 0.056 | 0.364 | 0.008 |
| Occasional/temporary | 136 | Delivery tenure -> PC | 0.367 | 0.220 | 0.509 | <.001 |
| Occasional/temporary | 136 | Daily hours -> PC | 0.166 | -0.001 | 0.330 | 0.052 |

Table S7J. R-squared values by work mode

| **Work mode** | **N** | **Endogenous construct** | **R2** | **Adjusted R2** | **Bootstrap CI 2.5%** | **Bootstrap CI 97.5%** |
| --- | --- | --- | --- | --- | --- | --- |
| Full-time | 299 | EEX | 0.207 | 0.193 | 0.141 | 0.304 |
| Full-time | 299 | EFD | 0.247 | 0.231 | 0.181 | 0.349 |
| Full-time | 299 | PC | 0.290 | 0.273 | 0.224 | 0.386 |
| Part-time | 215 | EEX | 0.166 | 0.146 | 0.095 | 0.287 |
| Part-time | 215 | EFD | 0.209 | 0.187 | 0.148 | 0.316 |
| Part-time | 215 | PC | 0.255 | 0.230 | 0.174 | 0.388 |
| Occasional/temporary | 136 | EEX | 0.267 | 0.239 | 0.169 | 0.423 |
| Occasional/temporary | 136 | EFD | 0.215 | 0.178 | 0.132 | 0.368 |
| Occasional/temporary | 136 | PC | 0.321 | 0.284 | 0.225 | 0.477 |

# S7.5 Group-specific measurement diagnostics

Table S7K. Reliability, convergent validity, and higher-order loadings by work mode

| **Work mode** | **N** | **Construct** | **Cronbach alpha** | **Composite reliability** | **AVE** | **Minimum loading** | **Maximum loading** |
| --- | --- | --- | --- | --- | --- | --- | --- |
| Full-time | 299 | TAC | 0.773 | 0.854 | 0.595 | 0.740 | 0.787 |
| Full-time | 299 | RAC | 0.769 | 0.853 | 0.593 | 0.719 | 0.812 |
| Full-time | 299 | TRC | 0.821 | 0.882 | 0.652 | 0.769 | 0.833 |
| Full-time | 299 | EEX | 0.775 | 0.855 | 0.597 | 0.743 | 0.803 |
| Full-time | 299 | EFD | 0.820 | 0.881 | 0.649 | 0.778 | 0.838 |
| Full-time | 299 | PC | 0.743 | 0.839 | 0.566 | 0.701 | 0.806 |
| Full-time | 299 | PAC -> TAC |  |  |  | 0.771 | 0.771 |
| Full-time | 299 | PAC -> RAC |  |  |  | 0.815 | 0.815 |
| Full-time | 299 | PAC -> TRC |  |  |  | 0.841 | 0.841 |
| Part-time | 215 | TAC | 0.733 | 0.834 | 0.557 | 0.707 | 0.778 |
| Part-time | 215 | RAC | 0.724 | 0.830 | 0.549 | 0.720 | 0.760 |
| Part-time | 215 | TRC | 0.720 | 0.827 | 0.544 | 0.687 | 0.792 |
| Part-time | 215 | EEX | 0.737 | 0.835 | 0.559 | 0.734 | 0.764 |
| Part-time | 215 | EFD | 0.829 | 0.887 | 0.662 | 0.782 | 0.853 |
| Part-time | 215 | PC | 0.717 | 0.825 | 0.542 | 0.717 | 0.761 |
| Part-time | 215 | PAC -> TAC |  |  |  | 0.681 | 0.681 |
| Part-time | 215 | PAC -> RAC |  |  |  | 0.769 | 0.769 |
| Part-time | 215 | PAC -> TRC |  |  |  | 0.729 | 0.729 |
| Occasional/temporary | 136 | TAC | 0.766 | 0.851 | 0.589 | 0.689 | 0.803 |
| Occasional/temporary | 136 | RAC | 0.739 | 0.839 | 0.565 | 0.722 | 0.770 |
| Occasional/temporary | 136 | TRC | 0.696 | 0.815 | 0.524 | 0.682 | 0.746 |
| Occasional/temporary | 136 | EEX | 0.751 | 0.843 | 0.574 | 0.719 | 0.803 |
| Occasional/temporary | 136 | EFD | 0.747 | 0.841 | 0.570 | 0.741 | 0.774 |
| Occasional/temporary | 136 | PC | 0.700 | 0.818 | 0.529 | 0.686 | 0.783 |
| Occasional/temporary | 136 | PAC -> TAC |  |  |  | 0.707 | 0.707 |
| Occasional/temporary | 136 | PAC -> RAC |  |  |  | 0.716 | 0.716 |
| Occasional/temporary | 136 | PAC -> TRC |  |  |  | 0.756 | 0.756 |

All group-level composite reliability and AVE values met conventional thresholds. Cronbach's alpha for TRC in the occasional/temporary group was 0.696, marginally below 0.70, while its composite reliability (0.815) and AVE (0.524) remained acceptable. This minor result reinforces the need to interpret the smallest subgroup cautiously.

# S7.6 Exploratory between-group contrasts

Table S7L. Exploratory pairwise bootstrap contrasts

| **Comparison** | **Association** | **Difference** | **Bootstrap SE** | **CI 2.5%** | **CI 97.5%** | **Two-tailed p** | **Interpretation note** |
| --- | --- | --- | --- | --- | --- | --- | --- |
| Full-time - Part-time | PAC -> EEX | -0.057 | 0.103 | -0.255 | 0.145 | 0.598 | Exploratory contrast; no formal MICOM-based PLS-MGA claim. |
| Full-time - Part-time | PAC -> EFD | 0.323 | 0.108 | 0.109 | 0.530 | 0.003 | Exploratory contrast; no formal MICOM-based PLS-MGA claim. |
| Full-time - Part-time | EEX -> EFD | -0.043 | 0.083 | -0.204 | 0.120 | 0.598 | Exploratory contrast; no formal MICOM-based PLS-MGA claim. |
| Full-time - Part-time | PAC -> PC | 0.037 | 0.108 | -0.176 | 0.246 | 0.742 | Exploratory contrast; no formal MICOM-based PLS-MGA claim. |
| Full-time - Part-time | EEX -> PC | -0.123 | 0.085 | -0.288 | 0.043 | 0.144 | Exploratory contrast; no formal MICOM-based PLS-MGA claim. |
| Full-time - Part-time | EFD -> PC | 0.122 | 0.085 | -0.046 | 0.291 | 0.149 | Exploratory contrast; no formal MICOM-based PLS-MGA claim. |
| Full-time - Part-time | PAC -> EEX -> PC | -0.032 | 0.025 | -0.085 | 0.013 | 0.186 | Exploratory contrast; no formal MICOM-based PLS-MGA claim. |
| Full-time - Part-time | PAC -> EFD -> PC | 0.078 | 0.028 | 0.029 | 0.138 | 0.001 | Exploratory contrast; no formal MICOM-based PLS-MGA claim. |
| Full-time - Part-time | PAC -> EEX -> EFD -> PC | -0.001 | 0.003 | -0.008 | 0.004 | 0.628 | Exploratory contrast; no formal MICOM-based PLS-MGA claim. |
| Full-time - Part-time | PAC total indirect -> PC | 0.044 | 0.037 | -0.026 | 0.120 | 0.221 | Exploratory contrast; no formal MICOM-based PLS-MGA claim. |
| Full-time - Part-time | PAC total association -> PC | 0.081 | 0.109 | -0.131 | 0.293 | 0.468 | Exploratory contrast; no formal MICOM-based PLS-MGA claim. |
| Full-time - Occasional/temporary | PAC -> EEX | 0.001 | 0.126 | -0.244 | 0.244 | 0.988 | Exploratory contrast; no formal MICOM-based PLS-MGA claim. |
| Full-time - Occasional/temporary | PAC -> EFD | 0.057 | 0.124 | -0.190 | 0.301 | 0.646 | Exploratory contrast; no formal MICOM-based PLS-MGA claim. |
| Full-time - Occasional/temporary | EEX -> EFD | 0.134 | 0.109 | -0.084 | 0.340 | 0.238 | Exploratory contrast; no formal MICOM-based PLS-MGA claim. |
| Full-time - Occasional/temporary | PAC -> PC | 0.128 | 0.122 | -0.105 | 0.371 | 0.286 | Exploratory contrast; no formal MICOM-based PLS-MGA claim. |
| Full-time - Occasional/temporary | EEX -> PC | 0.108 | 0.099 | -0.086 | 0.302 | 0.274 | Exploratory contrast; no formal MICOM-based PLS-MGA claim. |
| Full-time - Occasional/temporary | EFD -> PC | 0.341 | 0.095 | 0.150 | 0.524 | <.001 | Exploratory contrast; no formal MICOM-based PLS-MGA claim. |
| Full-time - Occasional/temporary | PAC -> EEX -> PC | 0.017 | 0.020 | -0.022 | 0.061 | 0.329 | Exploratory contrast; no formal MICOM-based PLS-MGA claim. |
| Full-time - Occasional/temporary | PAC -> EFD -> PC | 0.104 | 0.035 | 0.038 | 0.175 | 0.002 | Exploratory contrast; no formal MICOM-based PLS-MGA claim. |
| Full-time - Occasional/temporary | PAC -> EEX -> EFD -> PC | -0.003 | 0.004 | -0.014 | 0.003 | 0.349 | Exploratory contrast; no formal MICOM-based PLS-MGA claim. |
| Full-time - Occasional/temporary | PAC total indirect -> PC | 0.118 | 0.039 | 0.041 | 0.196 | 0.003 | Exploratory contrast; no formal MICOM-based PLS-MGA claim. |
| Full-time - Occasional/temporary | PAC total association -> PC | 0.246 | 0.116 | 0.018 | 0.473 | 0.036 | Exploratory contrast; no formal MICOM-based PLS-MGA claim. |
| Part-time - Occasional/temporary | PAC -> EEX | 0.058 | 0.123 | -0.185 | 0.299 | 0.665 | Exploratory contrast; no formal MICOM-based PLS-MGA claim. |
| Part-time - Occasional/temporary | PAC -> EFD | -0.265 | 0.127 | -0.513 | -0.010 | 0.040 | Exploratory contrast; no formal MICOM-based PLS-MGA claim. |
| Part-time - Occasional/temporary | EEX -> EFD | 0.177 | 0.114 | -0.052 | 0.395 | 0.137 | Exploratory contrast; no formal MICOM-based PLS-MGA claim. |
| Part-time - Occasional/temporary | PAC -> PC | 0.091 | 0.129 | -0.155 | 0.350 | 0.473 | Exploratory contrast; no formal MICOM-based PLS-MGA claim. |
| Part-time - Occasional/temporary | EEX -> PC | 0.231 | 0.108 | 0.024 | 0.446 | 0.029 | Exploratory contrast; no formal MICOM-based PLS-MGA claim. |
| Part-time - Occasional/temporary | EFD -> PC | 0.219 | 0.103 | 0.013 | 0.416 | 0.036 | Exploratory contrast; no formal MICOM-based PLS-MGA claim. |
| Part-time - Occasional/temporary | PAC -> EEX -> PC | 0.049 | 0.027 | -0.000 | 0.108 | 0.051 | Exploratory contrast; no formal MICOM-based PLS-MGA claim. |
| Part-time - Occasional/temporary | PAC -> EFD -> PC | 0.027 | 0.026 | -0.023 | 0.080 | 0.312 | Exploratory contrast; no formal MICOM-based PLS-MGA claim. |
| Part-time - Occasional/temporary | PAC -> EEX -> EFD -> PC | -0.002 | 0.004 | -0.012 | 0.005 | 0.599 | Exploratory contrast; no formal MICOM-based PLS-MGA claim. |
| Part-time - Occasional/temporary | PAC total indirect -> PC | 0.074 | 0.036 | 0.002 | 0.146 | 0.043 | Exploratory contrast; no formal MICOM-based PLS-MGA claim. |
| Part-time - Occasional/temporary | PAC total association -> PC | 0.165 | 0.125 | -0.076 | 0.412 | 0.181 | Exploratory contrast; no formal MICOM-based PLS-MGA claim. |

The contrasts identify several potentially meaningful differences, including a stronger PAC-EFD path among full-time than part-time workers and stronger EFD-PC and EEX-PC paths in full-time or part-time workers than in occasional/temporary workers. These comparisons are exploratory because the original study was not designed around formal multi-group measurement invariance. A path being significant in one group and nonsignificant in another is not, by itself, evidence of a statistically significant difference.

# S7.7 Interpretation boundary

In these aggregate-score sensitivity analyses, several focal associations persisted after accounting for work arrangement and demographic or work-exposure characteristics, while PAC coefficients attenuated. The coefficient products involving EFD were clearest among full-time workers, and those involving EEX among part-time workers. None of the focal paths to PC was significant among occasional/temporary workers, for whom age and delivery tenure explained a substantial share of PC variance. These post hoc patterns suggest heterogeneity requiring replication; they do not identify temporal routes, definitive moderation, or causal effects.
