# Supplementary material for: Dimensions of perceived algorithmic control and endorsement of minimum-only execution in platform delivery work
Source: Front Psychol. 2026 Oct 7;17:1937810. doi: 10.3389/fpsyg.2026.1937810 (PMC13683074; doi:10.3389/fpsyg.2026.1937810)
Supplement: Supplementary file 1 [file Data_Sheet_1.ZIP › Supplementary Appendix S8 Nonresponse and Sample Selection.docx]

**Supplementary Appendix S8**

*Non-response and sample-selection diagnostics*

# S8.1 Purpose and evidence boundary

Reviewer 1 requested station-specific response rates, an early-versus-late respondent comparison, and a comparison between the study sample and published rider-population figures. Recruitment began when the research team contacted a manager at a food-delivery station, explained the study, and obtained support for distribution. The manager circulated the questionnaire link through nationwide WeChat networks used by food-delivery and express-delivery workers and asked managers in other regions to forward the invitation in local work groups. The resulting process was a network-based group broadcast rather than a station-by-station sampling frame. The archived records contain 660 submitted questionnaires, 650 retained analytical cases, questionnaire completion duration in the historical file, and the original questionnaire sequence ID. They do not contain station identifiers, invitation denominators, refusal counts, unique station assignments, or submission timestamps. Consequently, station response rates and a timestamp-based early-late comparison cannot be reconstructed. The analyses below distinguish these unavailable quantities from the checks that can be performed.

The response-order comparison uses the first and last quartiles of retained cases ranked by original questionnaire sequence ID (n = 163 each). Welch tests and Hedges g were used for continuous construct scores. Gender, age group, and work mode were assessed with chi-square tests and Cramer's V. A first-versus-last-tertile comparison (n = 216 each) was conducted as a sensitivity check. This approach follows the logic of late-response diagnostics but is interpreted conservatively because questionnaire sequence is not a submission timestamp (Armstrong and Overton, 1977).

# S8.2 Recruitment and response-record availability

The open link could be re-forwarded across regions and work groups. In addition, platform delivery work in China is often overlapping: workers may deliver part-time for more than one platform and may combine food delivery with express or local instant delivery. The study's delivery-type and primary-platform fields therefore identify predominant self-reported arrangements rather than mutually exclusive occupational memberships. These features prevent station or platform membership from serving as a unique recruitment denominator.

Table S8A. Records available for response-rate and non-response diagnostics

| **Record or metric** | **Available** | **Value or evidence** | **Interpretation** |
| --- | --- | --- | --- |
| Initial submitted questionnaires | Yes | 660 | Number of submitted questionnaires before quality screening. |
| Final retained analytical questionnaires | Yes | 650 | Number retained after response-time and multivariate quality screening. |
| Analytic retention rate | Yes | 0.9848484848484849 | 98.5% of submitted questionnaires were retained; this is not a recruitment response rate. |
| Original questionnaire sequence ID | Yes | ID 1-660 with ten excluded IDs absent from the final file | Supports an order-based early/late proxy; it is not a timestamp. |
| Questionnaire completion duration | Historical 660 file only | DURATION_SEC | Used for quality screening, not for identifying early versus late calendar respondents. |
| Submission timestamp | No | No timestamp field in retained or historical analysis files | A timestamp-based early/late comparison cannot be reconstructed. |
| Station identifier | No | No station field retained | Station-specific submissions cannot be counted. |
| Number invited at each station | No | No invitation denominator retained | Station-specific response rates cannot be calculated. |
| Number declining at each station | No | No refusal count retained | Respondents cannot be directly compared with decliners. |
| Recruitment distribution pathway | Yes | Initial contact with one food-delivery-station manager; nationwide WeChat group circulation and regional manager forwarding | Network-based group broadcast rather than station-by-station sampling. |
| Unique station assignment | No | The link could be re-forwarded across regions and groups | Respondents cannot be assigned to one recruitment station. |
| Platform and delivery-role exclusivity | No | Workers may use multiple platforms or combine food, express, and local instant delivery | Recorded categories represent predominant arrangements, not mutually exclusive strata. |

# S8.3 Early-versus-late response-order comparison

Table S8B. First and last quartiles on focal constructs

| **Construct** | **Early M** | **Early SD** | **Late M** | **Late SD** | **Difference** | **95% CI lower** | **95% CI upper** | **Welch t** | **p** | **Hedges g** |
| --- | --- | --- | --- | --- | --- | --- | --- | --- | --- | --- |
| Temporal algorithmic control | 4.865 | 0.850 | 4.952 | 0.841 | -0.087 | -0.272 | 0.097 | -0.934 | 0.351 | -0.103 |
| Response algorithmic control | 5.144 | 0.847 | 5.278 | 0.872 | -0.133 | -0.321 | 0.054 | -1.401 | 0.162 | -0.155 |
| Trajectory algorithmic control | 4.923 | 0.796 | 5.058 | 0.958 | -0.135 | -0.327 | 0.057 | -1.384 | 0.167 | -0.153 |
| Perceived algorithmic control | 4.978 | 0.664 | 5.096 | 0.710 | -0.119 | -0.268 | 0.031 | -1.558 | 0.120 | -0.172 |
| Emotional exhaustion | 5.020 | 0.754 | 4.879 | 0.886 | 0.141 | -0.038 | 0.320 | 1.549 | 0.122 | 0.171 |
| Emotional feigning | 5.210 | 1.009 | 5.209 | 0.936 | 0.002 | -0.211 | 0.214 | 0.014 | 0.989 | 0.002 |
| Rule-bounded reduced discretionary effort | 5.923 | 0.713 | 5.914 | 0.766 | 0.009 | -0.152 | 0.170 | 0.112 | 0.911 | 0.012 |

Note. Early cases were the first 163 retained questionnaires in original sequence order (IDs 1-167); late cases were the last 163 retained questionnaires (IDs 494-660). Differences are early minus late. No comparison reached p < .05; the largest absolute Hedges g was .172.

Table S8C. Early and late quartiles on gender, age group, and work mode

| **Variable** | **Category** | **Early n** | **Early %** | **Late n** | **Late %** | **Chi-square** | **p** | **Cramer V** |
| --- | --- | --- | --- | --- | --- | --- | --- | --- |
| Gender | Male | 124 | 76.1 | 123 | 75.5 | 0.000 | 1.000 | 0.000 |
| Gender | Female | 39 | 23.9 | 40 | 24.5 | 0.000 | 1.000 | 0.000 |
| Age group | 18-25 | 37 | 22.7 | 34 | 20.9 | 1.653 | 0.799 | 0.071 |
| Age group | 26-35 | 77 | 47.2 | 74 | 45.4 | 1.653 | 0.799 | 0.071 |
| Age group | 36-45 | 39 | 23.9 | 39 | 23.9 | 1.653 | 0.799 | 0.071 |
| Age group | 46-55 | 8 | 4.9 | 12 | 7.4 | 1.653 | 0.799 | 0.071 |
| Age group | 56+ | 2 | 1.2 | 4 | 2.5 | 1.653 | 0.799 | 0.071 |
| Work mode | Full-time | 77 | 47.2 | 80 | 49.1 | 0.333 | 0.847 | 0.032 |
| Work mode | Part-time | 54 | 33.1 | 55 | 33.7 | 0.333 | 0.847 | 0.032 |
| Work mode | Occasional/temporary | 32 | 19.6 | 28 | 17.2 | 0.333 | 0.847 | 0.032 |

Note. The omnibus tests were nonsignificant for gender, chi-square(1) = 0.000, p = 1.000; age group, chi-square(4) = 1.653, p = .799; and work mode, chi-square(2) = 0.333, p = .847.

# S8.4 Tertile sensitivity analysis

Table S8D. First and last tertiles on focal constructs

| **Construct** | **Early M** | **Late M** | **Difference** | **95% CI** | **Welch t** | **p** | **Hedges g** |
| --- | --- | --- | --- | --- | --- | --- | --- |
| Temporal algorithmic control | 4.916 | 4.905 | 0.010 | [-0.154, 0.175] | 0.125 | 0.901 | 0.012 |
| Response algorithmic control | 5.203 | 5.252 | -0.050 | [-0.213, 0.113] | -0.599 | 0.549 | -0.058 |
| Trajectory algorithmic control | 4.988 | 5.044 | -0.056 | [-0.227, 0.116] | -0.636 | 0.525 | -0.061 |
| Perceived algorithmic control | 5.035 | 5.067 | -0.032 | [-0.167, 0.104] | -0.458 | 0.647 | -0.044 |
| Emotional exhaustion | 5.062 | 4.917 | 0.146 | [-0.007, 0.299] | 1.871 | 0.062 | 0.180 |
| Emotional feigning | 5.294 | 5.190 | 0.104 | [-0.079, 0.287] | 1.119 | 0.264 | 0.107 |
| Rule-bounded reduced discretionary effort | 5.927 | 5.898 | 0.029 | [-0.112, 0.170] | 0.404 | 0.686 | 0.039 |

Note. The first and last tertiles each contained 216 cases. No comparison reached p < .05; the smallest p value was .062 for EEX, and the largest absolute Hedges g was .180.

# S8.5 Descriptive comparison with published rider benchmarks

The benchmark comparison is descriptive. The study includes food delivery, express delivery, local instant delivery, and mixed delivery workers across multiple platforms. Published sources use different coverage periods and category definitions. The comparisons therefore identify directional representativeness issues; they are not weighting targets or formal population-equivalence tests.

Table S8E. Study sample and published benchmarks

| **Characteristic** | **Study sample** | **Published benchmark** | **Directional comparison** | **Comparability limitation** | **Source** |
| --- | --- | --- | --- | --- | --- |
| Age | Age 35 or younger: 440/650 (67.7%); age 46 or older: 51/650 (7.8%) | QuestMobile 2025: 61.8% of food-delivery workers were age 35 or younger. Meituan 2024: riders age 45 or older exceeded 10%. | The study sample is somewhat younger, particularly at the older tail. | Age cutoffs differ (46+ versus 45+); benchmark populations focus on food delivery, whereas the study includes express and local delivery. | QuestMobile/199IT (2025); Xu and Tang (2025) |
| Gender | Women: 176/650 (27.1%) | QuestMobile 2025: women represented 24.3% of food-delivery workers. | The study sample is broadly similar, with a modestly higher female share. | The benchmark concerns food-delivery workers nationally; the study includes multiple platforms and delivery types. | QuestMobile/199IT (2025) |
| Work mode | Full-time 46.0%; part-time 33.1%; occasional/temporary 20.9% (non-full-time total 54.0%) | Meituan monthly active riders in 2024: crowdsourced 71.52% and dedicated approximately 28.48%. | The study sample contains a larger structured/full-time segment and a smaller flexible segment than the platform benchmark suggests. | Dedicated/crowdsourced status is not equivalent to self-reported full-time/part-time/occasional work mode; the study is mixed-platform. | Zheng (2026), reporting Meituan report |

# S8.6 Interpretation

The available response-order diagnostics do not reveal a large early-late gradient among submitted questionnaires. However, they cannot evaluate riders who declined, did not see the link, or were not connected to the nationwide and regional WeChat networks through which the invitation was forwarded. Nor can respondents be uniquely assigned to one station, platform, or delivery type because cross-platform and mixed-delivery work is common. The external comparison suggests that the sample is broadly similar to a recent national food-delivery benchmark in gender composition, but somewhat younger and more oriented toward stable or structured work arrangements. These patterns are consistent with the network-based recruitment channel and justify limiting generalization to platform delivery workers reachable through comparable station-manager and delivery-worker group networks.

# S8.7 Sources

Armstrong, J. S., and Overton, T. S. (1977). Estimating nonresponse bias in mail surveys. Journal of Marketing Research, 14(3), 396-402. doi: 10.1177/002224377701400320.

199IT. (2025, September 11). QuestMobile: In July 2025, China had 14 million food-delivery workers and women represented 24.3%. Source summary of QuestMobile 2025 New Blue-Collar Workforce Insights. https://www.199it.com/archives/1784626.html

Xu, J., and Tang, H. (2025, July 25). Stable employment: A profile of millions of Meituan riders in 2024. Caixin. https://www.caixin.com/2025-07-25/102344920.html

Zheng, X. (2026, July 28). The food-delivery price war pauses: crowdsourced riders wait for orders. National Business Daily. https://www.nbd.com.cn/articles/2026-07-28/4524384.html
