# Supplementary material for: Dimensions of perceived algorithmic control and endorsement of minimum-only execution in platform delivery work
Source: Front Psychol. 2026 Oct 7;17:1937810. doi: 10.3389/fpsyg.2026.1937810 (PMC13683074; doi:10.3389/fpsyg.2026.1937810)
Supplement: Supplementary file 1 [file Data_Sheet_1.ZIP › Supplementary Appendix S9 Reverse Order.docx]

**Supplementary Appendix S9**

*Reverse statistical ordering of the earlier aggregate-score model*

# S9.1 Purpose

This appendix retains the earlier reverse-order sensitivity analysis of the aggregate PAC representation. It compares two recursive arrangements of PAC, EEX, EFD, and PC using the same final N = 650 item data. The comparison illustrates the ambiguity of assigning direction to cross-sectional associations; it does not establish either a forward or feedback process and is not a reverse-order analysis of the revised six-construct PLS model.

# S9.2 Model specifications

Table S9A. Recursive orderings and structural equations

| **Model** | Statistical ordering | **Equation 1** | **Equation 2** | **Equation 3** | **Interpretive purpose** |
| --- | --- | --- | --- | --- | --- |
| Original aggregate ordering | PAC -> EEX -> EFD -> PC | EEX ~ PAC | EFD ~ PAC + EEX | PC ~ PAC + EEX + EFD | Historical aggregate representation retained for comparison; arrows specify conditional equations. |
| Reverse ordering | PC -> PAC -> EEX -> EFD | PAC ~ PC | EEX ~ PC + PAC | EFD ~ PC + PAC + EEX | Reviewer-motivated reverse statistical ordering; temporal precedence is not established. |

Both specifications contain the same four constructs and six pairwise relations and differ in their recursive regression ordering. First-order blocks were represented by standardized first principal-component scores, and PAC by the standardized first principal component of TAC, RAC, and TRC scores. The equations were estimated by ordinary least squares. The reconstruction of the original ordering reproduced the archived SmartPLS direct coefficients and R² values within a maximum absolute difference of 0.011. Inference used 10,000 nonparametric bootstrap resamples, two-tailed sign-proportion tests, percentile 95% confidence intervals, and seed 20260819.

# S9.3 Fit comparison

Table S9B. Fit of the reported and reverse recursive orderings

| **Model** | **Ordering** | **Construct SRMR** | **Stage-2 SRMR** | **Log-likelihood** | **AIC** | **BIC** | **Delta AIC** | **Delta BIC** |
| --- | --- | --- | --- | --- | --- | --- | --- | --- |
| Original aggregate reconstruction | PAC -> EEX -> EFD -> PC | 0.000 | 0.073 | -3389.448 | 6806.895 | 6869.573 | 0.000 | 0.000 |
| Reverse aggregate reconstruction | PC -> PAC -> EEX -> EFD | 0.000 | 0.073 | -3389.448 | 6806.895 | 6869.573 | 0.000 | 0.000 |

Note. The archived estimated-model SRMR for the original higher-order SmartPLS specification was 0.089 and did not meet the more conservative 0.08 criterion. The stage-2 SRMR of 0.073 in this table belongs to the principal-component reconstruction and is used only to compare its two statistical orderings. It neither replaces the original 0.089 nor reports the fit of the revised native PLS model. Equal fit is expected because the two complete recursive score models represent the same covariance information.

# S9.4 Reverse-order path estimates

Table S9C. Direct paths and explained variance in the reverse-order model

| Association or quantity | **Estimate** | **Bootstrap SD** | **95% CI** | **Two-tailed p** |
| --- | --- | --- | --- | --- |
| PC -> PAC | 0.518 | 0.027 | [0.462, 0.569] | <.001 |
| PC -> EEX | 0.190 | 0.038 | [0.113, 0.262] | <.001 |
| PAC -> EEX | 0.361 | 0.038 | [0.285, 0.436] | <.001 |
| PC -> EFD | 0.202 | 0.039 | [0.124, 0.279] | <.001 |
| PAC -> EFD | 0.409 | 0.038 | [0.333, 0.483] | <.001 |
| EEX -> EFD | -0.012 | 0.036 | [-0.083, 0.060] | 0.741 |
| R2 PAC | 0.268 | 0.028 | [0.213, 0.324] | <.001 |
| R2 EEX | 0.238 | 0.030 | [0.182, 0.298] | <.001 |
| R2 EFD | 0.287 | 0.028 | [0.235, 0.345] | <.001 |

Table S9D. Indirect associations in the reverse-order model

| **Indirect association** | **Estimate** | **Bootstrap SD** | **95% CI** | **Two-tailed p** |
| --- | --- | --- | --- | --- |
| PC -> PAC -> EEX | 0.187 | 0.022 | [0.145, 0.232] | <.001 |
| PC -> PAC -> EFD | 0.212 | 0.024 | [0.165, 0.260] | <.001 |
| PC -> EEX -> EFD | -0.002 | 0.007 | [-0.017, 0.012] | 0.741 |
| PC -> PAC -> EEX -> EFD | -0.002 | 0.007 | [-0.016, 0.012] | 0.741 |

# S9.5 Interpretation

The reverse parameterization yielded a positive PC–PAC coefficient and statistically nonzero downstream associations. These coefficients do not show that minimum-only execution causes stronger perceived control. The two complete recursive score models had equal global fit, so this comparison does not select a temporal ordering. The result is specific to the aggregate-score reconstructions examined here; reverse and reciprocal processes remain possible.

Direct coefficients and their products are consequently interpreted as conditional associations. Repeated measurements, exogenous changes in platform rules, and linkage to independent behavioral records could help examine temporal and causal questions that the present survey cannot resolve.

# S9.6 Reproducibility files

R1_Comment9_Reverse_Order_Analysis_20260819.py; R1_Comment9_Reverse_Order_Bootstrap_Replicates_20260819.csv; R1_Comment9_Reverse_Order_Inference_Summary_20260819.csv; R1_Comment9_Reverse_Order_Fit_Comparison_20260819.csv; R1_Comment9_Reverse_Order_Point_Estimates_20260819.csv; R1_Comment9_Reverse_Order_Evidence_20260819.xlsx; R1_Comment9_Reverse_Order_Analysis_Run_Log.txt

# S9.7 References

Henley, A. B., Shook, C. L., and Peterson, M. (2006). The presence of equivalent models in strategic management research using structural equation modeling: Assessing and addressing the problem. Organizational Research Methods, 9(4), 516-535. doi: 10.1177/1094428106290195
Maxwell, S. E., and Cole, D. A. (2007). Bias in cross-sectional analyses of longitudinal mediation. Psychological Methods, 12(1), 23-44. doi: 10.1037/1082-989X.12.1.23
