# Supplementary material for: Dimensions of perceived algorithmic control and endorsement of minimum-only execution in platform delivery work
Source: Front Psychol. 2026 Oct 7;17:1937810. doi: 10.3389/fpsyg.2026.1937810 (PMC13683074; doi:10.3389/fpsyg.2026.1937810)
Supplement: Supplementary file 1 [file Data_Sheet_1.ZIP › Supplementary Appendix S10 Alternative Serial Order.docx]

**Supplementary Appendix S10**

**Alternative emotional ordering in the earlier aggregate-score model**

# **S10.1 Purpose**

This appendix retains the earlier sensitivity analysis that placed EFD before EEX in the aggregate PAC score model. The comparison responds to the alternative emotional ordering raised during review. Both the original and alternative arrangements are retained as supplementary statistical parameterizations; neither is treated as the primary revised dimension-level analysis or as evidence of temporal causality.

# **S10.2 Theoretical accounts motivating the comparison**

| Theoretical account | Literature basis | Interpretation in this study | Boundary |
| --- | --- | --- | --- |
| EEX -> EFD | Sayre et al. (2025) | Low energy resources may increase reliance on surface acting as a resource-conserving strategy and contribute to a loss spiral. | Does not establish that exhaustion precedes feigning in the present sample. |
| EFD -> EEX | Grandey (2003); Hülsheger and Schewe (2011); Xanthopoulou et al. (2018) | Surface acting/display regulation may consume resources and precede exhaustion or recovery need. | Does not establish that feigning precedes exhaustion in the present sample. |
| Reciprocal or episode-dependent dynamics | Both literatures | The two experiences may reinforce one another across service episodes. | Requires repeated within-person or multi-wave data. |

# **S10.3 Model specifications and estimation**

The earlier aggregate representation placed EEX before EFD; the alternative placed EFD before EEX. Both retained the PAC–EEX, PAC–EFD, PAC–PC, EEX–PC, and EFD–PC relations. First-order blocks were represented by standardized first principal-component scores, and PAC by the standardized first principal component of TAC, RAC, and TRC scores. Ordinary least squares equations were then estimated. This is a principal-component score reconstruction, not the native disjoint two-stage SmartPLS analysis. The reconstruction of the original ordering matched its archived principal coefficients and R² values within a maximum absolute difference of 0.011. Inference used 10,000 nonparametric bootstrap resamples, two-tailed sign-proportion tests, percentile 95% confidence intervals, and seed 20260820.

*Table S10A. Original and alternative statistical orderings*

| Model | Structural equations | Focal three-coefficient product |
| --- | --- | --- |
| Original aggregate reconstruction | EEX ~ PAC; EFD ~ PAC + EEX; PC ~ PAC + EEX + EFD | PAC -> EEX -> EFD -> PC |
| Alternative aggregate reconstruction | EFD ~ PAC; EEX ~ PAC + EFD; PC ~ PAC + EFD + EEX | PAC -> EFD -> EEX -> PC |

# **S10.4 Alternative-order estimates**

*Table S10B. Direct associations and explained variance in the alternative ordering*

| Path or quantity | Estimate | 95% CI lower | 95% CI upper | Two-tailed p |
| --- | --- | --- | --- | --- |
| PAC -> EFD | 0.508 | 0.450 | 0.561 | <.001 |
| PAC -> EEX | 0.447 | 0.372 | 0.518 | <.001 |
| EFD -> EEX | 0.025 | -0.050 | 0.102 | 0.521 |
| PAC -> PC | 0.341 | 0.266 | 0.413 | <.001 |
| EFD -> PC | 0.193 | 0.119 | 0.265 | <.001 |
| EEX -> PC | 0.171 | 0.101 | 0.241 | <.001 |
| R2 EFD | 0.258 | 0.203 | 0.315 | <.001 |
| R2 EEX | 0.212 | 0.158 | 0.271 | <.001 |
| R2 PC | 0.320 | 0.268 | 0.378 | <.001 |

*Table S10C. Specific indirect associations in the alternative ordering*

| Indirect association | Estimate | 95% CI lower | 95% CI upper | Two-tailed p |
| --- | --- | --- | --- | --- |
| PAC -> EFD -> PC | 0.098 | 0.061 | 0.137 | <.001 |
| PAC -> EEX -> PC | 0.077 | 0.043 | 0.112 | <.001 |
| PAC -> EFD -> EEX -> PC | 0.002 | -0.005 | 0.009 | 0.521 |

# **S10.5 Fit comparison and interpretation**

*Table S10D. Fit of the original and alternative aggregate-score reconstructions*

| Model | Stage-2 SRMR | Log-likelihood | AIC | BIC | ΔAIC | ΔBIC |
| --- | --- | --- | --- | --- | --- | --- |
| Original aggregate reconstruction | 0.073 | -3389.448 | 6806.895 | 6869.573 | 0.000 | 0.000 |
| Alternative aggregate reconstruction | 0.073 | -3389.448 | 6806.895 | 6869.573 | 0.000 | 0.000 |

In the alternative parameterization, the EFD–EEX coefficient and the PAC–EFD–EEX–PC product were not statistically distinguishable from zero. The products involving EEX alone and EFD alone were positive, with intervals excluding zero. The two complete recursive score models had identical fit because they represented the same pairwise covariance information with different regression orderings. These results do not favor a parallel temporal process or exclude reciprocal relationships. They describe the coefficient pattern in the earlier aggregate-score analysis and leave the temporal relationship between exhaustion and feigning unidentified.

The stage-2 SRMR of 0.073 belongs to these score reconstructions. It does not replace the original higher-order SmartPLS SRMR of 0.089 or the separately reported fit results of the revised native dimension-level model.

# **S10.6 Reproducibility files**

R2_Comment2_Alternative_Serial_Analysis_20260820.py; R2_Comment2_Alternative_Serial_Bootstrap_Replicates_20260820.csv; R2_Comment2_Alternative_Serial_Inference_Summary_20260820.csv; R2_Comment2_Alternative_Serial_Fit_Comparison_20260820.csv; R2_Comment2_Alternative_Serial_Point_Estimates_20260820.csv; R2_Comment2_Alternative_Serial_Evidence_20260820.xlsx; R2_Comment2_Alternative_Serial_Analysis_Run_Log.txt.

# **S10.7 References**

Grandey, A. A. (2003). When the show must go on: Surface acting and deep acting as determinants of emotional exhaustion and peer-rated service delivery. Academy of Management Journal, 46(1), 86-96. https://doi.org/10.2307/30040678

Hülsheger, U. R., & Schewe, A. F. (2011). On the costs and benefits of emotional labor: A meta-analysis of three decades of research. Journal of Occupational Health Psychology, 16(3), 361-389. https://doi.org/10.1037/a0022876

Sayre, G. M., Chi, N.-W., & Grandey, A. A. (2025). Surface acting loss spirals: Getting unstuck with recovery activities. Journal of Organizational Behavior, 46(5), 685-700. https://doi.org/10.1002/job.2859

Xanthopoulou, D., Bakker, A. B., Oerlemans, W. G. M., & Koszucka, M. (2018). Need for recovery after emotional labor: Differential effects of daily deep and surface acting. Journal of Organizational Behavior, 39(4), 481-494. https://doi.org/10.1002/job.2245
