# Supplementary material for: Dimensions of perceived algorithmic control and endorsement of minimum-only execution in platform delivery work
Source: Front Psychol. 2026 Oct 7;17:1937810. doi: 10.3389/fpsyg.2026.1937810 (PMC13683074; doi:10.3389/fpsyg.2026.1937810)
Supplement: Supplementary file 1 [file Data_Sheet_1.ZIP › Supplementary Appendix S11 Original Aggregate and CFA Diagnostic.docx]

**Supplementary Appendix S11. Original aggregate PAC analysis and common-representation diagnostic**

# **S11.1 Analytical history and original hypotheses**

The original analysis represented perceived algorithmic control (PAC) as a reflective higher-order construct measured by TAC, RAC, and TRC scores using a disjoint two-stage PLS approach. This appendix preserves its hypothesis identifiers, archived results, and associated analyses for comparison with the revised primary model, which enters the three dimensions jointly.

H1. Perceived algorithmic control is positively associated with rule-bounded reduced discretionary effort (PC) among platform delivery workers.

H2. In the specified aggregate PAC model, the product of the PAC–EEX and EEX–PC coefficients is positive.

H3. In the specified aggregate PAC model, the product of the PAC–EFD and EFD–PC coefficients is positive.

H4. In the specified aggregate PAC model, the product of the PAC–EEX, EEX–EFD, and EFD–PC coefficients is positive.

H2–H4 retain the original tested relationships and are expressed as coefficient products to clarify their statistical meaning. H1 was operationalized as the adjusted PAC coefficient in the PC equation. These statements do not identify a temporal process. The dimension-level research questions were introduced during revision and are not retrospective replacements for H1–H4.

# **S11.2 Archived original measurement and structural results**

The original results below were checked against final_smartpls_exported_results.xlsx in the original Supplementary Data File 1. This check confirms correspondence with the saved workbook; the old native project and complete bootstrap history were not recovered.

## **Table S11.1. Original item loadings and reliability**

| **Construct** | **Item** | **Measurement item (English reporting translation)** | **Factor loading** | **Cronbach's alpha** | **CR** | **AVE** |
| --- | --- | --- | --- | --- | --- | --- |
| TAC | TAC1 | The platform uses time limits and reminders to set the pace of delivery work. | 0.785 | 0.805 | 0.873 | 0.631 |
| TAC | TAC2 | While making deliveries, I speed up to keep pace with the system’s timing. | 0.800 |  |  |  |
| TAC | TAC3 | While making deliveries, I speed up when orders are assigned continuously. | 0.803 |  |  |  |
| TAC | TAC4 | While making deliveries, I adjust my pace according to the countdown. | 0.790 |  |  |  |
| RAC | RAC1 | The system uses order-assignment notifications and messages to prompt delivery workers to respond promptly. | 0.805 | 0.791 | 0.865 | 0.615 |
| RAC | RAC2 | While making deliveries, I quickly check order-assignment notifications. | 0.776 |  |  |  |
| RAC | RAC3 | While making deliveries, I reply to customer messages as soon as possible. | 0.806 |  |  |  |
| RAC | RAC4 | While making deliveries, I act on system prompts immediately. | 0.748 |  |  |  |
| TRC | TRC1 | The system uses location and route information to record the delivery process. | 0.817 | 0.823 | 0.883 | 0.653 |
| TRC | TRC2 | While making deliveries, I feel that my location is continuously recorded. | 0.811 |  |  |  |
| TRC | TRC3 | While making deliveries, I know that changes in my route are recorded. | 0.817 |  |  |  |
| TRC | TRC4 | While making deliveries, I know that stops or detours are recorded. | 0.788 |  |  |  |
| EEX | EEX1 | When the platform maintains a tight work pace, delivery workers are likely to feel emotionally tired. | 0.799 | 0.780 | 0.858 | 0.602 |
| EEX | EEX2 | When working at the platform’s pace, I feel emotionally tired. | 0.749 |  |  |  |
| EEX | EEX3 | When working at the platform’s pace, I feel drained. | 0.778 |  |  |  |
| EEX | EEX4 | When working at the platform’s pace, I feel emotionally worn out. | 0.776 |  |  |  |
| EFD | EFD1 | In platform work, delivery workers sometimes need to suppress their emotions and continue making deliveries. | 0.803 | 0.836 | 0.891 | 0.671 |
| EFD | EFD2 | When facing work demands, I suppress my dissatisfaction. | 0.822 |  |  |  |
| EFD | EFD3 | When facing work demands, I hold back my true emotions. | 0.811 |  |  |  |
| EFD | EFD4 | When facing work demands, I remain calm and continue making deliveries. | 0.839 |  |  |  |
| PC | PC1 | Within platform rules, delivery workers sometimes perform only the prescribed actions. | 0.757 | 0.767 | 0.851 | 0.589 |
| PC | PC2 | When carrying out platform requirements, I sometimes perform only the prescribed actions. | 0.752 |  |  |  |
| PC | PC3 | When carrying out platform requirements, I sometimes do only what is basically required. | 0.800 |  |  |  |
| PC | PC4 | When carrying out platform requirements, I sometimes simply complete the required procedure. | 0.759 |  |  |  |

Note. These archived values are retained for historical comparison only. The revised primary measurement statistics are in Table 1 and S12. Item translations remain the reporting translations of the administered questionnaire.

## **Table S11.1a. Archived and recalculated standardized alpha**

| **Construct** | **Archived alpha** | **Recalculated alpha** | **Absolute difference** |
| --- | --- | --- | --- |
| TAC | .805 | .802 | .003 |
| RAC | .791 | .790 | .001 |
| TRC | .823 | .824 | .001 |
| PC | .767 | .761 | .006 |

Note. Recalculated values are standardized alpha from the identified 650-case item dataset. At three decimals, the differences range from .001 to .006, and all four values remain above .70. The specific source of the historical differences is undetermined. Table S11.1 preserves the archived values; the main manuscript reports the verified current measurement results.

## **Table S11.2. Original aggregate-model HTMT**

| **Construct** | **EEX** | **EFD** | **PAC** | **PC** |
| --- | --- | --- | --- | --- |
| EEX |  |  |  |  |
| EFD | 0.305 |  |  |  |
| PAC | 0.597 | 0.649 |  |  |
| PC | 0.479 | 0.516 | 0.691 |  |

## **Table S11.3. Original explained variance**

| **Endogenous construct** | **R-square** |
| --- | --- |
| EEX | 0.213 |
| EFD | 0.265 |
| PC | 0.324 |

## **Table S11.4. Original structural direct associations**

| **Path** | **Original sample (O)** | **STDEV** | **T statistics** | **P values** | **VIF** |
| --- | --- | --- | --- | --- | --- |
| PAC → EEX | 0.462 | 0.031 | 14.943 | < .001 | 1.000 |
| PAC → EFD | 0.508 | 0.033 | 15.222 | < .001 | 1.271 |
| PAC → PC | 0.347 | 0.037 | 9.352 | < .001 | 1.622 |
| EEX → EFD | 0.015 | 0.036 | 0.422 | .673 | 1.271 |
| EEX → PC | 0.167 | 0.036 | 4.622 | < .001 | 1.272 |
| EFD → PC | 0.193 | 0.037 | 5.135 | < .001 | 1.360 |

## **Table S11.5. Original higher-order reflective loadings**

| **Dimension of PAC** | **Loading (O)** | **STDEV** | **T statistics** | **P values** |
| --- | --- | --- | --- | --- |
| Temporal algorithmic control (TAC) | 0.792 | 0.016 | 50.970 | < .001 |
| Response algorithmic control (RAC) | 0.821 | 0.013 | 65.049 | < .001 |
| Trajectory algorithmic control (TRC) | 0.837 | 0.013 | 66.795 | < .001 |

Note. TAC, RAC, and TRC rows are higher-order measurement loadings, not structural paths or tests of dimension-specific effects.

## **Table S11.6. Original specific indirect associations**

| **Hypothesis** | **Relationship** | **Original sample (O)** | **STDEV** | **T statistics** | **P values** | **2.5%** | **97.5%** | **Result** |
| --- | --- | --- | --- | --- | --- | --- | --- | --- |
| H2 | PAC → EEX → PC | 0.077 | 0.018 | 4.302 | < .001 | 0.044 | 0.114 | Supported |
| H3 | PAC → EFD → PC | 0.098 | 0.020 | 4.948 | < .001 | 0.061 | 0.138 | Supported |
| H4 | PAC → EEX → EFD → PC | 0.001 | 0.003 | 0.403 | .687 | -0.005 | 0.009 | Not supported |

Note. The original manuscript labeled these intervals bias-corrected. The saved workbook verifies the endpoints, but the original bootstrap settings were not recovered to independently verify that label. They are therefore identified here as the reported original 95% intervals. The revised model uses separately verified percentile intervals.

## **Table S11.7. Original IPMA**

| **Construct** | **Importance (SmartPLS total-effect statistic)** | **Performance** |
| --- | --- | --- |
| Emotional exhaustion (EEX) | 0.170 | 64.406 |
| Emotional feigning (EFD) | 0.193 | 70.957 |
| Perceived algorithmic control (PAC) | 0.523 | 58.154 |

Note. SmartPLS importance is the original IPMA total-effect statistic; it is not an intervention effect. PAC importance = .523, EFD = .193, and EEX = .170. The original figure is preserved in the protected submission baseline; its numerical information is retained in this table. This ranking is not a result of the new dimension-level model.

## **Table S11.8. Original control-adjusted and work-mode results**

| **Path / outcome** | **Full sample + controls** | **Full-time** | **Part-time** | **Occasional/temporary** |
| --- | --- | --- | --- | --- |
| PAC -> EEX | 0.193 (<.001) | 0.155 (.038) | 0.213 (.002) | 0.154 (.121) |
| PAC -> EFD | 0.223 (<.001) | 0.323 (<.001) | 0.000 (.988) | 0.266 (.011) |
| EEX -> EFD | -0.041 (.258) | -0.026 (.612) | 0.017 (.810) | -0.160 (.100) |
| PAC -> PC | 0.210 (<.001) | 0.224 (.001) | 0.187 (.021) | 0.096 (.348) |
| EEX -> PC | 0.138 (<.001) | 0.107 (.043) | 0.230 (.002) | -0.001 (.985) |
| EFD -> PC | 0.149 (<.001) | 0.240 (<.001) | 0.119 (.075) | -0.100 (.212) |
| R² EEX | 0.266 | 0.207 | 0.166 | 0.267 |
| R² EFD | 0.316 | 0.247 | 0.209 | 0.215 |
| R² PC | 0.340 | 0.290 | 0.255 | 0.321 |

Note. Values are standardized coefficients with two-tailed p values in parentheses except R² rows. Full-sample models include work mode, age, gender, tenure, and hours; within-group models include age, gender, tenure, and hours. These are historical score-reconstructed aggregate models. Complete intervals and exploratory group contrasts remain in S7.

In the original model, H1 was supported by the positive PAC–PC coefficient (.347, p < .001). H2 and H3 were supported by products of .077 (reported 95% CI [.044, .114]) and .098 ([.061, .138]), respectively. H4 was not supported: the three-coefficient product was .001, with a reported interval of [−.005, .009] and p = .687. Its small estimate is consistent with the small EEX–EFD coefficient (.015, p = .673), but the decision on H4 follows the interval for the product itself. It does not require a separate significance criterion for every constituent coefficient.

For archival transparency, .077 + .098 = .175, which is approximately 33.5% of the original IPMA total-association statistic .523; .347/.523 is approximately 66.3%. These are arithmetic comparisons across the original reported quantities and rounded values. They are not causal proportions mediated and are not carried into the revised model.

The archived estimated-model SRMR was .089 (SRMR sheet, G6; model label at G5). It did not meet the more conservative .08 criterion, indicating residual discrepancy. We do not treat it as evidence of close fit. The archived sheet does not provide a saturated-model column or bootstrap fit bounds. New-model SRMR, CFA fit, and reconstructed stage-2 fit do not replace this original value.

# **S11.3 Index to retained historical sensitivity evidence**

S2 retains the item-overlap and item-exclusion diagnostics with their available-source qualifications. These models were not rerun during the current content revision, and their missing historical outputs are not supplied by the new PLS model. Historical item diagnostics, including two corrected last-decimal descriptive statistics, retain their original source attribution in S2.

S5 retains the PCA-plus-OLS dimension reconstruction. TRC–PC has a zero-order score correlation of .313875, a conditional direct coefficient of −.182685, a total indirect association of .136408, and a total association of −.046277 (95% CI [−.125484, .031498], p = .239). A later fixed-score regression of PC on TAC, RAC, and TRC reproduces the total coefficient (difference approximately 2.1 × 10⁻¹⁶). The sign change is already present when the other dimensions are included; this does not identify a unique suppressor or decompose the zero-order correlation into the direct and indirect quantities. Different statistics in the historical S5 bootstrap file were sampled separately, so its same-row entries are not treated as jointly paired parameter draws.

S6 retains the earlier CFA and common-method-factor diagnostic and its aggregate structural sensitivity estimates. Those adjusted coefficients do not adjust the new dimension-level model for common method variance. The separate MLR common-PAC diagnostic in S11.4 evaluates the external-association information lost under a shared PAC factor.

S7 retains the background-adjusted and work-arrangement analyses introduced during peer review using the original aggregate model. In the full-sample control-adjusted analysis, the PAC–EEX, PAC–EFD, and PAC–PC coefficients were .193, .223, and .210, respectively; EEX–PC and EFD–PC were .138 and .149; EEX–EFD was −.041. The products through EEX and EFD were .027 (95% CI [.009, .050]) and .033 ([.012, .059]). R² was .266, .316, and .340 for EEX, EFD, and PC. PC R² in the full-time, part-time, and occasional/temporary groups was .290, .255, and .321. Differences in within-group significance alone are not tests of between-group differences. S7 preserves the explicitly exploratory pairwise contrasts. Unequal group sizes, particularly the smaller occasional/temporary group, and the absence of a formal measurement-invariance design limit these comparisons. These estimates are not control-adjusted results for the new three-dimension PLS model.

S8 retains the response-order comparisons and external descriptive benchmarks. The first-versus-last-quartile comparison had 163 observations in each group; all focal p values were at least .120. The lack of an observed response-order gradient cannot characterize unobserved refusals or workers outside the recruitment networks.

S9 compares original and reverse full recursive score orderings, with reconstructed SRMR = .073, log-likelihood = −3389.448, AIC = 6806.895, and BIC = 6869.573 for both. S10 reverses the EEX–EFD order in the aggregate score representation: the EFD–EEX coefficient was .025 (95% CI [−.050, .102], p = .521), and the PAC–EFD–EEX–PC product was .002 ([−.005, .009], p = .521). These PCA reconstructions preserve all six pairwise structural relations within their four-construct orderings. Equal fit cannot select a causal order, and intervals including zero in both emotional orderings do not establish parallel temporal processes.

# **S11.4 Revision-stage CFA common-representation diagnostic**

Two prespecified diagnostic models used the original N = 650 and 24 items, with four reflective indicators per factor and no item residual correlations. M0 freely correlated TAC, RAC, TRC, EEX, EFD, and PC. M1 added a common PAC factor for the first three dimensions and restricted their external relations to be represented through that factor; the three emotional/outcome factors remained freely related. This adds six covariance restrictions beyond the three-indicator higher-order block. The specification was fixed before these diagnostic estimates, during revision, rather than preregistered before data collection.

Models were estimated using MLR in lavaan 0.7-2 under R 4.6.1, treating the seven-point responses as approximately continuous. Both converged without warnings or detected improper solutions. The comparison used Satorra's 2000 delta, scaled-and-shifted procedure; it was not obtained by subtracting the two scaled chi-square values directly.

| **Statistic** | **M0 six correlated factors** | **M1 common PAC restrictions** |
| --- | --- | --- |
| Free parameters | 87 | 81 |
| df | 237 | 243 |
| Conventional chi-square | 204.012 | 307.661 |
| MLR scaled chi-square | 205.699 | 309.789 |
| Robust CFI | 1.00000 | .98835 |
| Robust RMSEA | .00000 | .02049 |
| 90% robust RMSEA CI | [.00000, .00501] | [.01271, .02701] |
| SRMR | .02270 | .03642 |

The robust difference was chi-square(6) = 102.495, p = 7.56 × 10⁻²⁰. M1 retained good approximate fit, but the shared representation lost information relative to M0. Positive standardized higher-order loadings (.771, .786, .814) did not eliminate that loss. The following latent correlations show where the restrictions changed the pattern; they are CFA correlations, not PLS path coefficients or score correlations.

| **Relation** | **M0** | **M1** |
| --- | --- | --- |
| TAC–EEX | .393 | .471 |
| RAC–EEX | .421 | .480 |
| TRC–EEX | .606 | .497 |
| TAC–EFD | .518 | .493 |
| RAC–EFD | .486 | .502 |
| TRC–EFD | .514 | .520 |
| TAC–PC | .656 | .518 |
| RAC–PC | .577 | .528 |
| TRC–PC | .397 | .547 |

The item-block residual RMS increased from .022 to .069 for TAC–PC, from .026 to .078 for TRC–PC, and from .022 to .059 for TRC–EEX. These are descriptive local diagnostics, not separate tests of each difference. Together with the item meanings, they motivate keeping the domains separate in the primary analysis. The loss of information is compatible with shared variation among the three domains.

The revised model represents TAC, RAC, and TRC as separate first-order reflective constructs and does not posit a formative PAC composite. The common-PAC diagnostic tests restrictions on external associations under a shared factor; it does not establish a formative PAC alternative. The CFA comparison uses a different estimator and purpose from the native PLS structural analysis.
