# Supplementary material for: Dimensions of perceived algorithmic control and endorsement of minimum-only execution in platform delivery work
Source: Front Psychol. 2026 Oct 7;17:1937810. doi: 10.3389/fpsyg.2026.1937810 (PMC13683074; doi:10.3389/fpsyg.2026.1937810)
Supplement: Supplementary file 1 [file Data_Sheet_1.ZIP › Supplementary Appendix S12 Revised Dimension Native PLS.docx]

**Supplementary Appendix S12. Native results for the revised joint dimension PLS model**

The revised primary model uses the original 650 cases and 24 items in six four-indicator reflective blocks. TAC, RAC, and TRC are entered jointly. Ordinary PLS estimation was followed by complete bootstrapping in SmartPLS 4.1.1.8. No item deletion, extra structural path, alternative estimator, or new sample was introduced.

# **S12.1 Specification and execution record**

EEX ~ TAC + RAC + TRC

EFD ~ TAC + RAC + TRC + EEX

PC ~ TAC + RAC + TRC + EEX + EFD

The algorithm used path weighting, standardized results, initial weights of 1, a maximum of 3,000 iterations, and a 10⁻⁷ stopping threshold. The recorded weights met that threshold at iteration 6. All six outer-weighting entries are AUTOMATIC. The bootstrap used complete output, 10,000 saved parameter draws, percentile intervals, two-tailed tests, alpha = .05, parallel processing, and a fixed-seed option. The numerical seed and separate attempt/failure counts are not displayed in the export. The reported parameter intervals use the native percentile endpoints rather than the separately exported bias-corrected intervals.

The 24 item columns and all 15,600 values were compared with the imported 650-row CSV, with identical order and values. The 44 structural original-sample estimates match the ordinary PLS export. Twenty saved-result tables, including three model-fit tables, contain 10,000 draws each, with no missing or nonfinite values among 2,110,000 entries. Re-summarization of the 44 structural estimates reproduces their means, population-denominator bootstrap standard deviations, and native percentile endpoints. The fit-result means and HI95/HI99 bounds were checked separately. This verifies the saved results; it does not reconstruct the unexported case-index list for each draw.

# **S12.2 Complete structural parameters**

| **Parameter type** | **Association** | **O** | **SD** | **t** | **p** | **95% percentile CI** | **Native summary / interval rows** |
| --- | --- | --- | --- | --- | --- | --- | --- |
| direct | EEX -> EFD | 0.02568555 | 0.03753825 | 0.68425 | .494 | [-0.04758282, 0.09991646] | B13:G13 / B30:F30 |
| direct | EEX -> PC | 0.23448074 | 0.03439156 | 6.81797 | <.001 | [0.16811286, 0.30236452] | B14:G14 / B31:F31 |
| direct | EFD -> PC | 0.18878694 | 0.03724659 | 5.06857 | <.001 | [0.11518296, 0.26094382] | B15:G15 / B32:F32 |
| direct | RAC -> EEX | 0.07063572 | 0.04250060 | 1.66199 | .097 | [-0.01468105, 0.15205526] | B16:G16 / B33:F33 |
| direct | RAC -> EFD | 0.16355971 | 0.04236165 | 3.86103 | <.001 | [0.07972233, 0.24635754] | B17:G17 / B34:F34 |
| direct | RAC -> PC | 0.24230633 | 0.03869364 | 6.26217 | <.001 | [0.16710163, 0.31839059] | B18:G18 / B35:F35 |
| direct | TAC -> EEX | 0.07518617 | 0.03995010 | 1.88200 | .060 | [-0.00214075, 0.15589432] | B19:G19 / B36:F36 |
| direct | TAC -> EFD | 0.24096996 | 0.04015462 | 6.00105 | <.001 | [0.16209069, 0.31749841] | B20:G20 / B37:F37 |
| direct | TAC -> PC | 0.33918275 | 0.03715764 | 9.12821 | <.001 | [0.26523971, 0.41279802] | B21:G21 / B38:F38 |
| direct | TRC -> EEX | 0.41148028 | 0.04060295 | 10.13425 | <.001 | [0.33144576, 0.48897189] | B22:G22 / B39:F39 |
| direct | TRC -> EFD | 0.20454184 | 0.04522497 | 4.52276 | <.001 | [0.11718136, 0.29428266] | B23:G23 / B40:F40 |
| direct | TRC -> PC | -0.18336664 | 0.04291629 | 4.27266 | <.001 | [-0.26864696, -0.10034291] | B24:G24 / B41:F41 |
| total_indirect | EEX -> PC | 0.00484910 | 0.00734462 | 0.66022 | .509 | [-0.00886963, 0.02005922] | B71:G71 / B83:F83 |
| total_indirect | RAC -> EFD | 0.00181432 | 0.00333592 | 0.54387 | .587 | [-0.00377944, 0.00973992] | B72:G72 / B84:F84 |
| total_indirect | RAC -> PC | 0.04778317 | 0.01378621 | 3.46601 | <.001 | [0.02147284, 0.07624789] | B73:G73 / B85:F85 |
| total_indirect | TAC -> EFD | 0.00193120 | 0.00332578 | 0.58067 | .561 | [-0.00420476, 0.00936055] | B74:G74 / B86:F86 |
| total_indirect | TAC -> PC | 0.06348628 | 0.01526228 | 4.15969 | <.001 | [0.03552419, 0.09535238] | B75:G75 / B87:F87 |
| total_indirect | TRC -> EFD | 0.01056910 | 0.01566979 | 0.67449 | .500 | [-0.01964876, 0.04189941] | B76:G76 / B88:F88 |
| total_indirect | TRC -> PC | 0.13709434 | 0.02064966 | 6.63906 | <.001 | [0.09964337, 0.17972124] | B77:G77 / B89:F89 |
| specific_indirect | EEX -> EFD -> PC | 0.00484910 | 0.00734462 | 0.66022 | .509 | [-0.00886963, 0.02005922] | B109:G109 / B127:F127 |
| specific_indirect | RAC -> EEX -> EFD | 0.00181432 | 0.00333592 | 0.54387 | .587 | [-0.00377944, 0.00973992] | B110:G110 / B128:F128 |
| specific_indirect | RAC -> EFD -> PC | 0.03087794 | 0.00980638 | 3.14876 | .002 | [0.01369470, 0.05172908] | B111:G111 / B129:F129 |
| specific_indirect | RAC -> EEX -> PC | 0.01656272 | 0.01018788 | 1.62573 | .104 | [-0.00352666, 0.03705960] | B112:G112 / B130:F130 |
| specific_indirect | TAC -> EEX -> EFD | 0.00193120 | 0.00332578 | 0.58067 | .561 | [-0.00420476, 0.00936055] | B113:G113 / B131:F131 |
| specific_indirect | TAC -> EFD -> PC | 0.04549198 | 0.01147933 | 3.96295 | <.001 | [0.02516338, 0.06956665] | B114:G114 / B132:F132 |
| specific_indirect | TAC -> EEX -> PC | 0.01762971 | 0.01001604 | 1.76015 | .078 | [-0.00047095, 0.03904704] | B115:G115 / B133:F133 |
| specific_indirect | TRC -> EEX -> EFD | 0.01056910 | 0.01566979 | 0.67449 | .500 | [-0.01964876, 0.04189941] | B116:G116 / B134:F134 |
| specific_indirect | TRC -> EFD -> PC | 0.03861483 | 0.01210225 | 3.19071 | .001 | [0.01813912, 0.06483044] | B117:G117 / B135:F135 |
| specific_indirect | TRC -> EEX -> PC | 0.09648420 | 0.01751470 | 5.50876 | <.001 | [0.06467059, 0.13294547] | B118:G118 / B136:F136 |
| specific_indirect | TRC -> EEX -> EFD -> PC | 0.00199531 | 0.00306528 | 0.65094 | .515 | [-0.00368074, 0.00845354] | B119:G119 / B137:F137 |
| specific_indirect | RAC -> EEX -> EFD -> PC | 0.00034252 | 0.00064400 | 0.53186 | .595 | [-0.00070099, 0.00192436] | B120:G120 / B138:F138 |
| specific_indirect | TAC -> EEX -> EFD -> PC | 0.00036458 | 0.00064941 | 0.56141 | .575 | [-0.00077430, 0.00185887] | B121:G121 / B139:F139 |
| total_effect | EEX -> EFD | 0.02568555 | 0.03753825 | 0.68425 | .494 | [-0.04758282, 0.09991646] | B165:G165 / B182:F182 |
| total_effect | EEX -> PC | 0.23932984 | 0.03461093 | 6.91486 | <.001 | [0.17140490, 0.30760774] | B166:G166 / B183:F183 |
| total_effect | EFD -> PC | 0.18878694 | 0.03724659 | 5.06857 | <.001 | [0.11518296, 0.26094382] | B167:G167 / B184:F184 |
| total_effect | RAC -> EEX | 0.07063572 | 0.04250060 | 1.66199 | .097 | [-0.01468105, 0.15205526] | B168:G168 / B185:F185 |
| total_effect | RAC -> EFD | 0.16537403 | 0.04246147 | 3.89468 | <.001 | [0.08172029, 0.24811219] | B169:G169 / B186:F186 |
| total_effect | RAC -> PC | 0.29008950 | 0.03889736 | 7.45782 | <.001 | [0.21345786, 0.36530067] | B170:G170 / B187:F187 |
| total_effect | TAC -> EEX | 0.07518617 | 0.03995010 | 1.88200 | .060 | [-0.00214075, 0.15589432] | B171:G171 / B188:F188 |
| total_effect | TAC -> EFD | 0.24290116 | 0.03981639 | 6.10053 | <.001 | [0.16498255, 0.31912417] | B172:G172 / B189:F189 |
| total_effect | TAC -> PC | 0.40266903 | 0.03648916 | 11.03531 | <.001 | [0.33077731, 0.47427377] | B173:G173 / B190:F190 |
| total_effect | TRC -> EEX | 0.41148028 | 0.04060295 | 10.13425 | <.001 | [0.33144576, 0.48897189] | B174:G174 / B191:F191 |
| total_effect | TRC -> EFD | 0.21511094 | 0.04140060 | 5.19584 | <.001 | [0.13474942, 0.29752252] | B175:G175 / B192:F192 |
| total_effect | TRC -> PC | -0.04627231 | 0.03945746 | 1.17271 | .241 | [-0.12533162, 0.03200470] | B176:G176 / B193:F193 |

O is the original-sample standardized estimate. All cell references in this table refer to the sheet 完整 in the archived complete bootstrap workbook. Software total_effect labels are retained in the source data but are interpreted here as model-specific total associations. The 13 product identities, 12 total-indirect sums (including five near-zero rounding-residual columns), and 12 direct-plus-indirect identities were checked at each saved draw: 370,000 comparisons agreed within 10⁻¹². Only seven origin–outcome pairs have substantive indirect paths. The five rounding-residual columns are not additional paths.

# **S12.3 Measurement statistics, explained variance, and fit**

## **Table 1. Measurement items and reflective measurement statistics**

### **Panel A. Administered item reporting translations and native PLS loadings**

| **Construct** | **Item** | **English reporting translation** | **Loading** |
| --- | --- | --- | --- |
| TAC | TAC1 | The platform uses time limits and reminders to set the pace of delivery work. | 0.7841 |
| TAC | TAC2 | While making deliveries, I speed up to keep pace with the system’s timing. | 0.7952 |
| TAC | TAC3 | While making deliveries, I speed up when orders are assigned continuously. | 0.8111 |
| TAC | TAC4 | While making deliveries, I adjust my pace according to the countdown. | 0.7786 |
| RAC | RAC1 | The system uses order-assignment notifications and messages to prompt delivery workers to respond promptly. | 0.8055 |
| RAC | RAC2 | While making deliveries, I quickly check order-assignment notifications. | 0.7845 |
| RAC | RAC3 | While making deliveries, I reply to customer messages as soon as possible. | 0.7983 |
| RAC | RAC4 | While making deliveries, I act on system prompts immediately. | 0.7446 |
| TRC | TRC1 | The system uses location and route information to record the delivery process. | 0.8142 |
| TRC | TRC2 | While making deliveries, I feel that my location is continuously recorded. | 0.8146 |
| TRC | TRC3 | While making deliveries, I know that changes in my route are recorded. | 0.8179 |
| TRC | TRC4 | While making deliveries, I know that stops or detours are recorded. | 0.7901 |
| EEX | EEX1 | When the platform maintains a tight work pace, delivery workers are likely to feel emotionally tired. | 0.7926 |
| EEX | EEX2 | When working at the platform’s pace, I feel emotionally tired. | 0.7442 |
| EEX | EEX3 | When working at the platform’s pace, I feel drained. | 0.7885 |
| EEX | EEX4 | When working at the platform’s pace, I feel emotionally worn out. | 0.7757 |
| EFD | EFD1 | In platform work, delivery workers sometimes need to suppress their emotions and continue making deliveries. | 0.8015 |
| EFD | EFD2 | When facing work demands, I suppress my dissatisfaction. | 0.8252 |
| EFD | EFD3 | When facing work demands, I hold back my true emotions. | 0.8089 |
| EFD | EFD4 | When facing work demands, I remain calm and continue making deliveries. | 0.8381 |
| PC | PC1 | Within platform rules, delivery workers sometimes perform only the prescribed actions. | 0.7555 |
| PC | PC2 | When carrying out platform requirements, I sometimes perform only the prescribed actions. | 0.7451 |
| PC | PC3 | When carrying out platform requirements, I sometimes do only what is basically required. | 0.7884 |
| PC | PC4 | When carrying out platform requirements, I sometimes simply complete the required procedure. | 0.7628 |

### **Panel B. Construct statistics**

| **Construct** | **Standardized alpha** | **rho_A** | **rho_C** | **AVE** |
| --- | --- | --- | --- | --- |
| TAC | 0.8024 | 0.8038 | 0.8709 | 0.6278 |
| RAC | 0.7901 | 0.7915 | 0.8641 | 0.6140 |
| TRC | 0.8244 | 0.8248 | 0.8836 | 0.6549 |
| EEX | 0.7798 | 0.7845 | 0.8578 | 0.6014 |
| EFD | 0.8359 | 0.8374 | 0.8903 | 0.6700 |
| PC | 0.7607 | 0.7610 | 0.8479 | 0.5824 |

Note. N = 650; four reflective indicators per construct. Item wording is preserved from the English reporting translations of the administered Chinese questionnaire in S1. Values refer to the joint dimension model. Construct definitions and measurement scope are described in Section 3.2. rho_C is composite reliability.

## **Table 2. HTMT ratios among the six measured constructs**

| **Construct pair** | **HTMT** | **95% percentile CI** |
| --- | --- | --- |
| EFD – EEX | 0.3124 | [0.2290, 0.3952] |
| PC – EEX | 0.4885 | [0.4061, 0.5656] |
| PC – EFD | 0.5145 | [0.4369, 0.5903] |
| RAC – EEX | 0.4200 | [0.3343, 0.5029] |
| RAC – EFD | 0.4863 | [0.4063, 0.5622] |
| RAC – PC | 0.5808 | [0.5079, 0.6515] |
| TAC – EEX | 0.3888 | [0.2998, 0.4752] |
| TAC – EFD | 0.5193 | [0.4435, 0.5920] |
| TAC – PC | 0.6572 | [0.5900, 0.7205] |
| TAC – RAC | 0.5795 | [0.5095, 0.6457] |
| TRC – EEX | 0.6058 | [0.5359, 0.6721] |
| TRC – EFD | 0.5135 | [0.4417, 0.5820] |
| TRC – PC | 0.3958 | [0.3128, 0.4747] |
| TRC – RAC | 0.6827 | [0.6192, 0.7417] |
| TRC – TAC | 0.6127 | [0.5425, 0.6773] |

Note. The upper limit was below .85 for every pair. This diagnostic concerns the measured constructs and does not establish distinctiveness from unmeasured adjacent constructs.

## **Table 3. Revised structural model and fit assessment**

### **Panel A. Explained variance**

| **Outcome** | **R²** | **Adjusted R²** | **95% percentile CI for R²** |
| --- | --- | --- | --- |
| EEX | 0.2478 | 0.2443 | [0.1970, 0.3108] |
| EFD | 0.2616 | 0.2570 | [0.2124, 0.3236] |
| PC | 0.3924 | 0.3876 | [0.3424, 0.4540] |

### **Panel B. Conditional direct associations**

| **Association** | **Beta** | **Bootstrap SD** | **p** | **95% percentile CI** | **Inner VIF** |
| --- | --- | --- | --- | --- | --- |
| EEX -> EFD | 0.0257 | 0.0375 | .494 | [-0.0476, 0.0999] | 1.3294 |
| EEX -> PC | 0.2345 | 0.0344 | <.001 | [0.1681, 0.3024] | 1.3303 |
| EFD -> PC | 0.1888 | 0.0372 | <.001 | [0.1152, 0.2609] | 1.3542 |
| RAC -> EEX | 0.0706 | 0.0425 | .097 | [-0.0147, 0.1521] | 1.5401 |
| RAC -> EFD | 0.1636 | 0.0424 | <.001 | [0.0797, 0.2464] | 1.5467 |
| RAC -> PC | 0.2423 | 0.0387 | <.001 | [0.1671, 0.3184] | 1.5829 |
| TAC -> EEX | 0.0752 | 0.0400 | .060 | [-0.0021, 0.1559] | 1.4260 |
| TAC -> EFD | 0.2410 | 0.0402 | <.001 | [0.1621, 0.3175] | 1.4336 |
| TAC -> PC | 0.3392 | 0.0372 | <.001 | [0.2652, 0.4128] | 1.5122 |
| TRC -> EEX | 0.4115 | 0.0406 | <.001 | [0.3314, 0.4890] | 1.6122 |
| TRC -> EFD | 0.2045 | 0.0452 | <.001 | [0.1172, 0.2943] | 1.8373 |
| TRC -> PC | -0.1834 | 0.0429 | <.001 | [-0.2686, -0.1003] | 1.8939 |

### **Panel C. Approximate and bootstrap fit evidence**

| **Model** | **Criterion** | **Original value** | **HI95** | **HI99** | **Comparison** |
| --- | --- | --- | --- | --- | --- |
| Saturated | SRMR | 0.049243 | 0.041823 | 0.042769 | Above HI99 |
| Estimated | SRMR | 0.049243 | 0.041823 | 0.042769 | Above HI99 |
| Saturated | d_ULS | 0.727447 | 0.524749 | 0.548747 | Above HI99 |
| Estimated | d_ULS | 0.727447 | 0.524749 | 0.548747 | Above HI99 |
| Saturated | d_G | 0.219479 | 0.219742 | 0.225452 | Below HI95 |
| Estimated | d_G | 0.219479 | 0.219742 | 0.225452 | Below HI95 |

Note. Native ordinary PLS, N = 650, 10,000 saved bootstrap parameter draws. Parameter intervals are two-tailed 95% percentile intervals. HI95 and HI99 are one-sided reference quantiles from the adapted Bollen–Stine model-fit bootstrap, not parameter confidence intervals. Approximate SRMR below .08 coexists with SRMR and d_ULS values above HI99; the fit diagnostics do not uniformly support the specification. No causal interpretation is assigned to the software path directions.

## **Table 4. Specific indirect associations in the revised model**

| **Specified product** | **Estimate** | **Bootstrap SD** | **p** | **95% percentile CI** |
| --- | --- | --- | --- | --- |
| EEX -> EFD -> PC | 0.0048 | 0.0073 | .509 | [-0.0089, 0.0201] |
| RAC -> EEX -> EFD | 0.0018 | 0.0033 | .587 | [-0.0038, 0.0097] |
| RAC -> EFD -> PC | 0.0309 | 0.0098 | .002 | [0.0137, 0.0517] |
| RAC -> EEX -> PC | 0.0166 | 0.0102 | .104 | [-0.0035, 0.0371] |
| TAC -> EEX -> EFD | 0.0019 | 0.0033 | .561 | [-0.0042, 0.0094] |
| TAC -> EFD -> PC | 0.0455 | 0.0115 | <.001 | [0.0252, 0.0696] |
| TAC -> EEX -> PC | 0.0176 | 0.0100 | .078 | [-0.0005, 0.0390] |
| TRC -> EEX -> EFD | 0.0106 | 0.0157 | .500 | [-0.0196, 0.0419] |
| TRC -> EFD -> PC | 0.0386 | 0.0121 | .001 | [0.0181, 0.0648] |
| TRC -> EEX -> PC | 0.0965 | 0.0175 | <.001 | [0.0647, 0.1329] |
| TRC -> EEX -> EFD -> PC | 0.0020 | 0.0031 | .515 | [-0.0037, 0.0085] |
| RAC -> EEX -> EFD -> PC | 0.0003 | 0.0006 | .595 | [-0.0007, 0.0019] |
| TAC -> EEX -> EFD -> PC | 0.0004 | 0.0006 | .575 | [-0.0008, 0.0019] |

Note. All 13 coefficient products implied by the 12 specified paths are reported, including nine dimension-to-PC products; four intervals exclude zero. Inference uses the prespecified percentile intervals. For TAC–EEX–PC, p = .078 and the unrounded lower endpoint is −.00047095; the exported bias-corrected interval is not used for inference. All three dimension–EEX–EFD–PC intervals include zero. These estimates describe associations within one survey wave, leaving temporal ordering unresolved.

## **Table 5. Direct, total indirect, and total associations with PC**

| **Dimension** | **Component** | **Estimate** | **p** | **95% percentile CI** |
| --- | --- | --- | --- | --- |
| TAC | Direct | 0.3392 | <.001 | [0.2652, 0.4128] |
| TAC | Total indirect | 0.0635 | <.001 | [0.0355, 0.0954] |
| TAC | Total | 0.4027 | <.001 | [0.3308, 0.4743] |
| RAC | Direct | 0.2423 | <.001 | [0.1671, 0.3184] |
| RAC | Total indirect | 0.0478 | <.001 | [0.0215, 0.0762] |
| RAC | Total | 0.2901 | <.001 | [0.2135, 0.3653] |
| TRC | Direct | -0.1834 | <.001 | [-0.2686, -0.1003] |
| TRC | Total indirect | 0.1371 | <.001 | [0.0996, 0.1797] |
| TRC | Total | -0.0463 | .241 | [-0.1253, 0.0320] |

Note. Total indirect is the sum of the three dimension-to-PC products; total is direct plus total indirect. The total quantity is conditional on the specified joint-dimension structure and is not the zero-order correlation. The native TRC–PC construct-score correlation is .31446121. TRC has opposite-signed direct and total indirect components, but its total interval includes zero.

# **S12.4 Native construct correlations and cross-loadings**

The following correlations are from the ordinary PLS export, rather than the earlier PCA reconstruction. The diagonal square roots of AVE from the Fornell–Larcker display are excluded.

| **Construct** | **Construct** | **Score correlation** | **Source cell** |
| --- | --- | --- | --- |
| EFD | EEX | 0.25524808 | 完整!C2401 |
| PC | EEX | 0.37986572 | 完整!C2402 |
| PC | EFD | 0.41093770 | 完整!D2402 |
| RAC | EEX | 0.33230178 | 完整!C2403 |
| RAC | EFD | 0.39620480 | 完整!D2403 |
| RAC | PC | 0.45055803 | 完整!E2403 |
| TAC | EEX | 0.31294898 | 完整!C2404 |
| TAC | EFD | 0.42652577 | 完整!D2404 |
| TAC | PC | 0.51358993 | 完整!E2404 |
| TAC | RAC | 0.46188923 | 完整!F2404 |
| TRC | EEX | 0.48791995 | 完整!C2405 |
| TRC | EFD | 0.42741202 | 完整!D2405 |
| TRC | PC | 0.31446121 | 完整!E2405 |
| TRC | RAC | 0.55151701 | 完整!F2405 |
| TRC | TAC | 0.49853404 | 完整!G2405 |

| **Item** | **EEX** | **EFD** | **PC** | **RAC** | **TAC** | **TRC** |
| --- | --- | --- | --- | --- | --- | --- |
| EEX1 | 0.792575 | 0.224543 | 0.313928 | 0.283341 | 0.300495 | 0.392080 |
| EEX2 | 0.744242 | 0.162918 | 0.245088 | 0.215268 | 0.182107 | 0.334810 |
| EEX3 | 0.788463 | 0.206653 | 0.330194 | 0.264464 | 0.276332 | 0.390691 |
| EEX4 | 0.775667 | 0.191244 | 0.279856 | 0.260992 | 0.197355 | 0.390574 |
| EFD1 | 0.181896 | 0.801459 | 0.326469 | 0.280915 | 0.330927 | 0.309291 |
| EFD2 | 0.219190 | 0.825236 | 0.372352 | 0.349157 | 0.352636 | 0.354437 |
| EFD3 | 0.241699 | 0.808916 | 0.332368 | 0.319495 | 0.361877 | 0.356619 |
| EFD4 | 0.190318 | 0.838056 | 0.311746 | 0.343675 | 0.349888 | 0.376444 |
| PC1 | 0.314728 | 0.314781 | 0.755465 | 0.335146 | 0.374527 | 0.224323 |
| PC2 | 0.253275 | 0.336675 | 0.745146 | 0.347262 | 0.385408 | 0.222431 |
| PC3 | 0.332822 | 0.300255 | 0.788434 | 0.354837 | 0.403571 | 0.291389 |
| PC4 | 0.257257 | 0.302905 | 0.762831 | 0.337850 | 0.404132 | 0.220591 |
| RAC1 | 0.286059 | 0.310575 | 0.363718 | 0.805549 | 0.372913 | 0.431928 |
| RAC2 | 0.255944 | 0.295364 | 0.380342 | 0.784485 | 0.362292 | 0.435297 |
| RAC3 | 0.253276 | 0.335078 | 0.338061 | 0.798274 | 0.384000 | 0.450950 |
| RAC4 | 0.245069 | 0.301050 | 0.328714 | 0.744566 | 0.326863 | 0.410019 |
| TAC1 | 0.247218 | 0.330679 | 0.390870 | 0.343664 | 0.784069 | 0.394155 |
| TAC2 | 0.244440 | 0.327508 | 0.410820 | 0.366898 | 0.795180 | 0.376580 |
| TAC3 | 0.270155 | 0.372963 | 0.419986 | 0.377644 | 0.811069 | 0.398653 |
| TAC4 | 0.228287 | 0.317996 | 0.405542 | 0.375360 | 0.778635 | 0.411579 |
| TRC1 | 0.380916 | 0.347222 | 0.239855 | 0.429748 | 0.413075 | 0.814198 |
| TRC2 | 0.399764 | 0.364827 | 0.283072 | 0.464987 | 0.433266 | 0.814607 |
| TRC3 | 0.400925 | 0.332242 | 0.235766 | 0.424368 | 0.390880 | 0.817894 |
| TRC4 | 0.397260 | 0.338021 | 0.257085 | 0.464379 | 0.374966 | 0.790137 |

# **S12.5 Source identification and reporting boundary**

| **File** | **SHA256** |
| --- | --- |
| 05_PLS_algorithm_full.xlsx | 198B856ABCD635164F1DFC30BBB50DE6BB79D527687ACBCC99D8B358929994A8 |
| 07_PAC三维联合模型_Bootstrap10000_完整结果_20260911.xlsx | 43D0EA87C5C4015C59CC3AE89875C428D0695E11A3F001B4798E7773FA0FAD08 |
| 02_PAC_dimensions_N650_24items.csv | ADED1419FFBC2F3B9DC7150687A9D50D18FE44853F67B9067E5B6C6CEFA22DF0 |

The ordinary and bootstrap exports are preserved without rewriting. The new estimated and saturated SRMR values both round to .0492, but SRMR and d_ULS exceed their HI99 reference bounds. The d_G value is below HI95. These results are reported together and do not establish uniform exact fit. The .089 SRMR of the original higher-order model remains attributed to that original model in S11. The interpretation of the adapted Bollen–Stine reference bounds follows the [SmartPLS model-fit documentation](https://www.smartpls.com/documentation/algorithms-and-techniques/validity-and-model-fit/model-fit/), accessed September 11, 2026.
